# Cyclometalated iridium complexes-catalyzed acceptorless dehydrogenative coupling reaction: construction of quinoline derivatives and evaluation of their antimicrobial activities

**DOI:** 10.3762/bjoc.18.159

**Published:** 2022-10-27

**Authors:** Hongling Shui, Yuhong Zhong, Renshi Luo, Zhanyi Zhang, Jiuzhong Huang, Ping Yang, Nianhua Luo

**Affiliations:** 1 School of Pharmacy, Gannan Medical University, Ganzhou, 341000, Jiangxi Province, P. R. China; 2 Institute of Microbiology, Guangdong Academy of Sciences, Guangzhou 510070, P. R. China

**Keywords:** acceptorless dehydrogenative coupling reaction, antibacterial, cyclometalated iridium complexes, quinolines

## Abstract

The acceptorless dehydrogenative coupling (ADC) reaction is an efficient method for synthesizing quinoline and its derivatives. In this paper, various substituted quinolines were synthesized from 2-aminobenzyl alcohols and aryl/heteroaryl/alkyl secondary alcohols in one pot via a cyclometalated iridium-catalyzed ADC reaction. This method has some advantages, such as easy availability of raw materials, mild reaction conditions, wide range of substrates, and environmental friendliness which conforms to the principles of green chemistry. Furthermore, a gram-scale experiment with low catalyst loading offers the potential to access the aryl/heteroaryl quinolones in suitable amounts. In addition, the antibacterial and antifungal activities of the synthesized quinolines were evaluated in vitro, and the experimental results showed that the antibacterial activities of compounds **3ab**, **3ad**, and **3ah** against Gram-positive bacteria and compound **3ck** against *C. albicans* were better than the reference drug norfloxacin.

## Introduction

**Figure 1:**
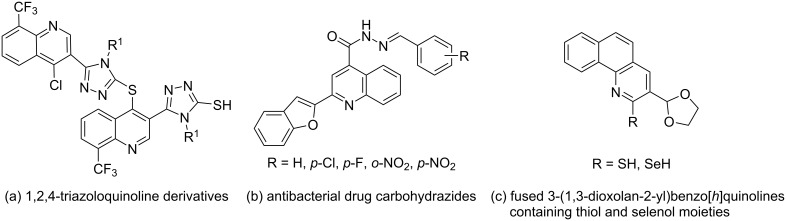
Some new quinoline antibacterial drugs.

As an important class of heterocyclic compounds, quinoline and its derivatives widely exist in natural products. They have a wide range of biological activities, such as antibacterial [1], anti-inflammatory [2], antitumor [3], antihepatitis C (HCV) [4], antituberculosis (TB) [5], antimalarial [6], and anti-Alzheimer's disease (AD) [7]. Among these biological activities, their antibacterial effect is more prominent. As we know, antimicrobial agents are a significant source to overcome bacterial infections, but overuse will lead to drug resistance [8], so it is necessary to synthesize new antibacterial compounds to overcome this problem. Quinolines whose physical and chemical properties and pharmacological activities could be improved by structural modifications are used as important antibacterial agents. The compounds are characterized by high efficiency, low toxicity, and low residue, and play an important role in pharmacy and medicine. Therefore, it is still of great significance to develop new and broad-spectrum quinoline antibacterial agents, and the research on antibacterial quinolines is one of the most promising and dynamic research fields in contemporary anti-infective therapy. For example, Eswaran's group [9] synthesized some 1,2,4-triazoquinoline derivatives, and the biological activity evaluation showed that most of the compounds had a higher antibacterial activity (the optimal MIC value was 6.25 mg/mL) against Gram-positive bacteria, Gram-negative bacteria and all tested fungi than the standard ciprofloxacin (Figure 1a). Bodke's group [10] synthesized a series of carbohydrazide derivatives through reaction of 2-methylbenzofuran-2-quinoline-4-carboxylate with hydrazine hydrate in refluxing ethanol (Figure 1b). All compounds showed higher activity against *Staphylococcus aureus* than ampicillin and the optimal MIC value was 0.064 mg/mL. In addition, Aravinda's group [11] prepared 3-(1,3-dioxolan-2-yl)benzo[*h*]quinolines containing thiol and selenol groups in one pot by microwave irradiation, and tested the antibacterial activity of the compounds. The results showed that the antibacterial effect of some compounds was better than ciprofloxacin (Figure 1c).

In recent years, the synthesis of quinolines has received great attention and remarkable achievements have been made to produce quinolones by various methods: Skraup reaction [12], Doebner–Miller reaction [13], Combes synthesis method [14], Conrad–Limpach reaction [15], Pfitzinger reaction [16], and Friedländer reaction [17]. Among these syntheses, the Friedländer reaction [17] is one of the most commonly used methods for the synthesis of quinolines. However, it has the disadvantages of harsh reaction conditions and low yields owing to the reactivity of *o*-aminobenzaldehyde when used as raw material. In order to solve such problems, chemists have developed ADC reactions catalyzed by metal complexes (such as Ir [18–19], Ru [20–24], Re [25], Mn [26–27], Pd [28], Ni [29], Cu [30], etc.) to synthesize quinolines using *o*-aminobenzyl alcohol as starting material.

ADC reactions have the advantages of high atom economy, simple operation, clean and green, and have become a research hotspot [31–35]. Cyclometalated iridium complexes with good catalytic efficiency and selectivity are very effective catalysts in ADC reactions. Moreover, these catalysts are easy to synthesize and stable to air [36], and have good operability and reproducibility [37–38]. In recent years, our research group has carried out relevant research on ADC reactions catalyzed by cyclometalated iridium complexes and obtained some interesting research results [39].

**Figure 2:**
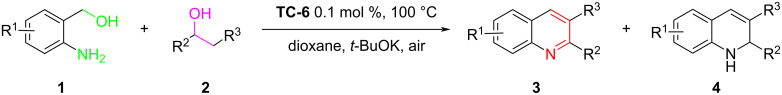
Cyclometalated iridium-catalyzed ADC reaction of *o*-aminobenzyl alcohols and secondary alcohols.

In previous studies [39–42], we found that cyclometalated iridium catalysts can effectively catalyze the dehydrogenation of alcohols to produce carbonyl compounds and hydrogen gas. Therefore, we used cyclometalated iridium complex (**TC-6**) to catalyze the ADC reaction of *o*-aminobenzyl alcohols **1** and aryl/heteroaryl/alkyl secondary alcohols **2** that allowed for the efficient synthesis of a series of quinolines **3** (up to 95% yield and >99:1 selectivity) (Figure 2). A preliminary evaluation of the compounds’ potential antibacterial activity was also performed.

## Results and Discussion

We started our research with the ADC reaction of 2-aminobenzyl alcohol (**1a**) with 1-phenylethanol (**2a**) as model reaction in the presence of various cyclometalated iridium complexes **TC-1**–**TC-6** (Table 1). Encouragingly, employing **TC-1** as the catalyst, toluene as the solvent and *t*-BuOK as the base at 100 °C, quinoline **3aa** was obtained in 73% yield accompanied by 27% yield of 1,2-dihydroquinoline **4aa** (Table 1, entry 1). Then, several other cyclometalated iridium complexes were studied. The catalysts **TC-2** and **TC-4** containing electron-donating ligands provided quinoline **3aa** in higher chemoselectivity and yield (Table 1, entries 2 and 4). On the contrary, the catalysts **TC-3** and **TC-5** containing electron-withdrawing ligands offered lower chemoselectivity and yield (Table 1, entries 3 and 5). Further catalyst screening revealed that **TC-6** (6-methoxy) is the best catalyst for the ADC reaction affording the product in a yield of 95% (Table 1, entry 6). On the other hand, when no catalyst was added to the reaction system under the above conditions, the reaction also proceeded, but the chemical selectivity and yield were significantly lower (Table 1, entry 7).

**Table 1:** Optimization of catalyst for ADC reaction of 2-aminobenzyl alcohol and 1-phenylethanol.^a^

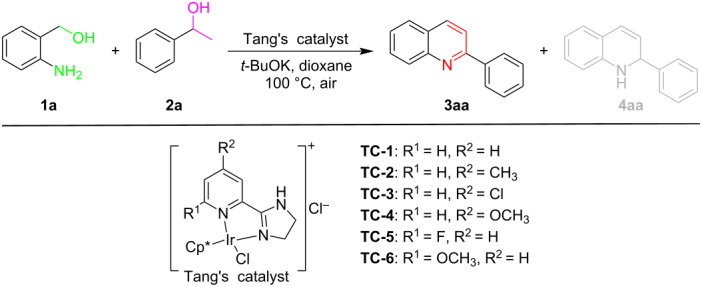				

Entry	Tang’s catalyst	Time (h)	**3aa**:**4aa**^b^	Yield of **3aa**^b^ (%)

1	**TC-1**	24	73:27	73
2	**TC-2**	24	79:21	79
3	**TC-3**	24	56:44	56
4	**TC-4**	24	82:18	82
5	**TC-5**	24	59:41	59
**6**	**TC-6**	**24**	**95:5**	**95 (93)** **^c^**
7^d^	–	48	51:49	51

^a^Reaction conditions: **1a** (1.1 mmol), **2a** (1.0 mmol), *t*-BuOK (1.0 mmol), dioxane (3 mL) and Tang’s catalyst (0.1 mol %) at 100 °C for 24 h. ^b^Determined by GC–MS. ^c^Yield of isolated product **3aa**. ^d^Reaction performed without Tang’s catalyst.

In order to obtain optimal conditions, the bases, reaction medium, and temperature were also surveyed (Table 2). First, several bases were examined and the results showed that different bases have different effects on the chemoselectivity and yield of the reaction. The weak bases including HCO_2_Na, CH_3_CO_2_K, and Na_2_CO_3_, resulted in decreased yields of quinoline **3aa** (Table 2, entries 1–3). Interestingly, the chemoselectivity of the reaction and product yield were significantly improved with strong bases, such as NaOH, KOH, or *t*-BuOK (Table 2, entries 4–6). To our excitement, a loading of 1.1 equiv of *t*-BuOK delivered the product **3aa** in the yield of 96% with perfect selectivity (Table 2, entries 6–8).

**Table 2:** Studies of reaction parameters in the iridium-catalyzed ADC reaction.^a^

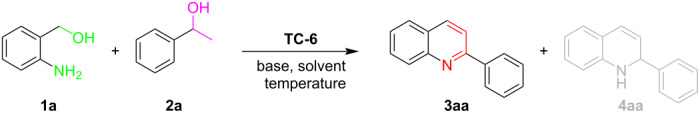						

Entry	Base	Solvent	Temperature (°C)	Time (h)	**3aa**:**4aa**^b^	Yield of **3aa**^b^ (%)

1	CH_3_CO_2_K	1,4-dioxane	100	24	74:26	74
2	HCO_2_Na	1,4-dioxane	100	24	69:31	69
3	Na_2_CO_3_	1,4-dioxane	100	24	76:24	76
4	NaOH	1,4-dioxane	100	24	82:18	82
5	KOH	1,4-dioxane	100	24	93:7	93
6	*t*-BuOK	1,4-dioxane	100	24	95:5	95
**7** **^c^**	***t*** **-BuOK**	**1,4-dioxane**	**100**	**24**	**>99:1**	**>99 (96)** **^d^**
8^e^	*t*-BuOK	1,4-dioxane	100	24	94:6	94
9^c^	*t*-BuOK	toluene	100	24	90:10	90
10^c^	*t*-BuOK	THF	80	24	81:19	81
11^c^	*t*-BuOK	DMF	100	24	69:31	69
12^c^	*t*-BuOK	H_2_O	100	24	83:17	83
13^c^	*t*-BuOK	1,4-dioxane	80	36	87:13	87
14^c,f^	*t*-BuOK	1,4-dioxane	100	48	>99:1	>99

^a^Reaction conditions: **1a** (1.1 mmol), **2a** (1.0 mmol), base, solvent (3 mL), and **TC-6** (0.1 mol %) at 100 °C for 24 h. ^b^Determined by GC–MS. ^c^1.1 mmol *t*-BuOK was used. ^d^Yield of isolated product **3aa**. ^e^0.8 mmol *t*-BuOK was used. ^f^0.01 mol % **TC-6** was used.

Afterward, we further screened the solvent and catalyst loading (Table 2, entries 7, 9–12, and 14) and the results showed that 1,4-dioxane was the most favorable solvent for the outcome of product **3aa**, even when the catalyst loading was decreased to 0.01 mol % (Table 2, entry 14). All other solvents screened resulted in lower product yield (Table 2, entries 9–12). Finally, we examined the effect of temperature on the reaction and found that decreasing the reaction temperature hindered the production of compound **3aa** (Table 2, entry 13).

Based on the screening of above reaction conditions, we obtained the optimal catalytic system, with 0.1 mol % **TC-6** as the catalyst, 1.1 equiv of *t*-BuOK as the base, and 1,4-dioxane as reaction solvent. Under the optimal reaction conditions, we investigated the universality of the cyclometalated iridium-catalyzed ADC reaction by expanding the range of substrates (Table 3). It can be seen that quinoline compounds **3** were obtained with excellent yield and chemoselectivity through the cyclometalated iridium-catalyzed ADC reaction of 2-aminobenzyl alcohol and different substituted aromatic secondary alcohols including electron-donating (Me, OMe) and electron-withdrawing substituents (F, Cl, Br) as the substrate (Table 3, entries 1–24). Aromatic secondary alcohols substituted with electron-donating groups led to higher chemoselectivities and yields of the products (Table 3, entries 2–5) than the aryl secondary alcohols and aminobenzyl alcohol with electron-withdrawing groups (Table 3, entries 8, 11, 12, 15, 16, 19, 20, 23, and 24). Meanwhile, the heteroaromatic secondary alcohols **2i–n** could also be employed in the cyclometalated iridium-catalyzed system obtaining the products **3ai–an** with excellent yield and chemoselectivity (Table 3, entries 26–42). The results showed that the yield and chemoselectivity was higher when the heteroaromatic secondary alcohols and aminobenzyl alcohols have electron-donating groups (Table 3, entries 27, 30, 31, 34, 35, 39, and 42). On the contrary, with the electron-withdrawing group, the yield and chemoselectivity of the reaction were relatively lower (Table 3, entries 28, 29, 32, 33, 36, 37, and 40). It is worth noting that high conversions were also accomplished when 1-cyclohexylethanol and pentan-1-ol were employed in this catalytic system (Table 3, entries 43 and 44).

**Table 3:** Cyclometalated iridium-catalyzed ADC reaction of various 2-aminobenzyl alcohols and secondary alcohols.^a^

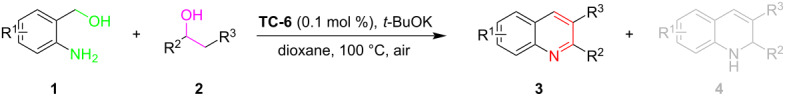					

Entry	**1**	**2**	Time (h)	**3**:**4**^b^	Yield of **3**^c^ (%)

1	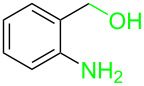	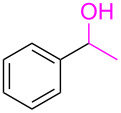	16	>99:1	(**3aa**) 96
2	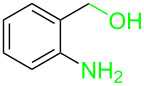	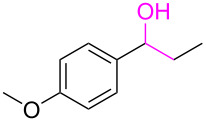	14	97:3	(**3ab**) 95
3	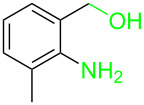	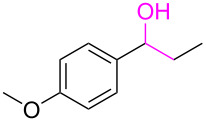	18	92:8	(**3bb**) 92
4	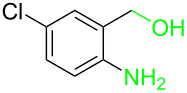	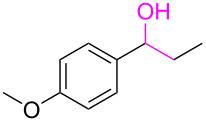	20	93:7	(**3cb**) 93
5	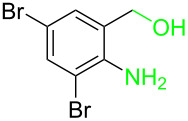	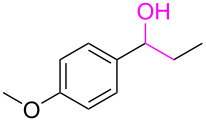	18	91:9	(**3db**) 91
6	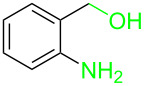	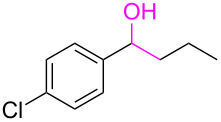	10	94:6	(**3ac**) 94
7	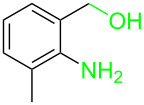	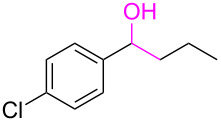	8	95:5	(**3bc**) 95
8	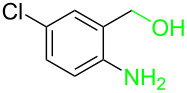	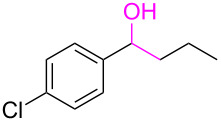	18	91:9	(**3cc**) 91
9	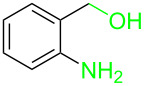	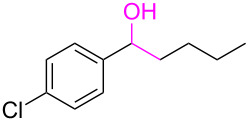	16	93:7	(**3ad**) 93
10	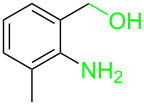	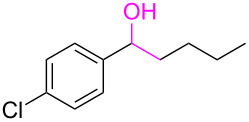	18	95:5	(**3bd**) 95
11	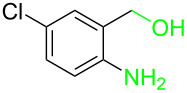	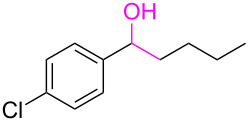	20	91:9	(**3cd**) 91
12	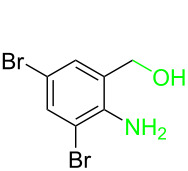	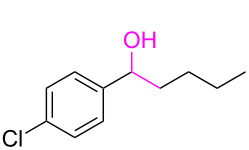	16	92:8	(**3dd**) 92
13	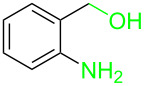	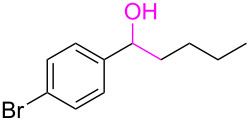	10	93:7	(**3ae**) 93
14	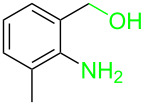	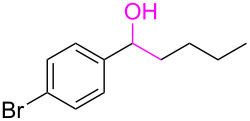	14	95:5	(**3be**) 95
15	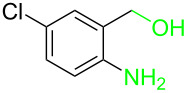	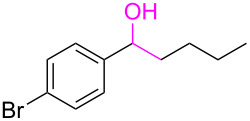	16	90:10	(**3ce**) 90
16	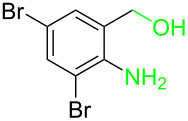	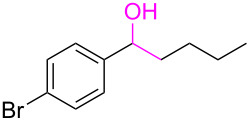	20	91:9	(**3de**) 91
17	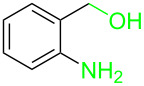	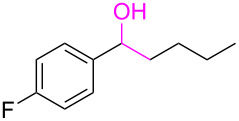	8	88:12	(**3af**) 88
18	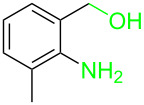	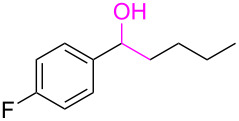	17	93:7	(**3bf**) 93
19	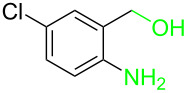	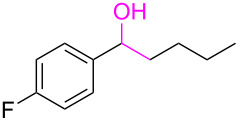	20	95:5	(**3cf**) 95
20	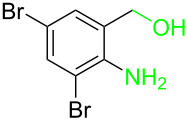	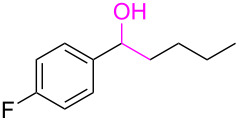	18	88:12	(**3df**) 88
21	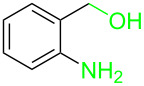	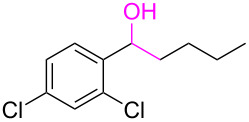	18	90:10	(**3ag**) 90
22	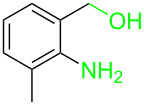	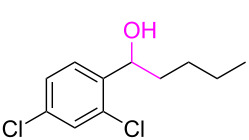	20	93:7	(**3bg**) 93
23	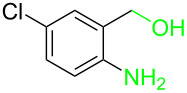	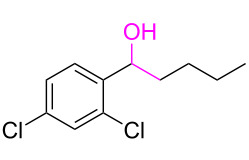	22	89:11	(**3cg**) 89
24	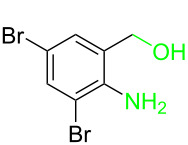	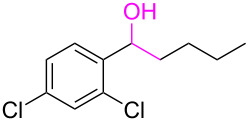	20	91:9	(**3dg**) 91
25	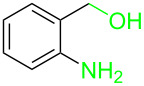	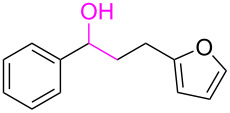	16	89:11	(**3ah**) 89
26	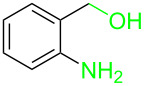	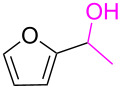	16	94:6	(**3ai**) 94
27	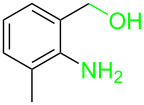	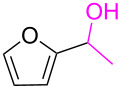	18	94:6	(**3bi**) 94
28	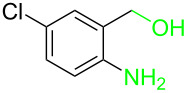	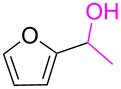	12	92:8	(**3ci**) 92
29	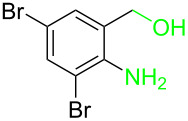	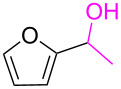	20	90:10	(**3di**) 90
30	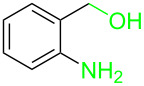	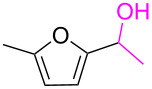	8	96:4	(**3aj**) 96
31	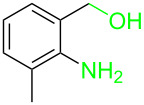	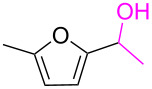	12	93:7	(**3bj**) 93
32	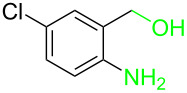	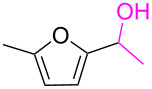	18	92:8	(**3cj**) 92
33	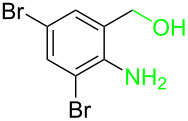	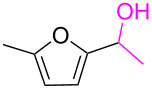	18	91:9	(**3dj**) 91
34	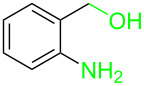	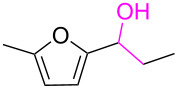	16	97:3	(**3ak**) 97
35	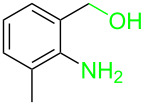	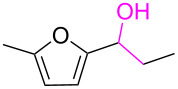	18	95:5	(**3bk**) 95
36	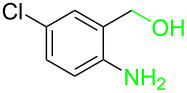	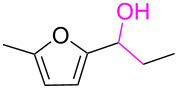	16	93:7	(**3ck**) 93
37	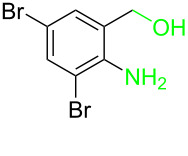	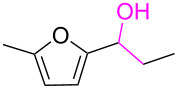	20	91:9	(**3dk**) 91
38	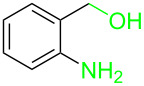	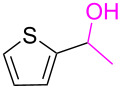	18	92:8	(**3al**) 92
39	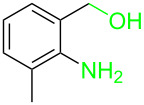	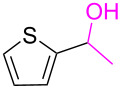	16	95:5	(**3bl**) 95
40	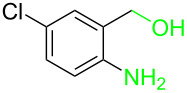	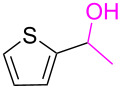	20	92:8	(**3cl**) 92
41	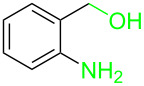	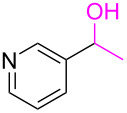	22	94:6	(**3am**) 94
42	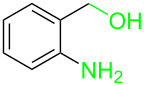	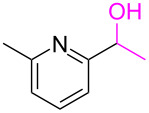	22	96:4	(**3an**) 96
43	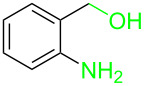	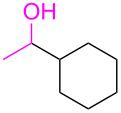	20	97:3	(**3ao**) 88
44	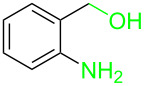	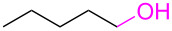	21	98:2	(**3ap**) 86

^a^Reaction conditions: a mixture of **1** (1.1 mmol), **2** (1.0 mmol), *t*-BuOK (1.0 mmol), dioxane (3 mL), and **TC-6** (0.1 mol %) at 100 °C. ^b^Determined by GC–MS. ^c^Yield of isolated product **3**.

**Figure 3:**
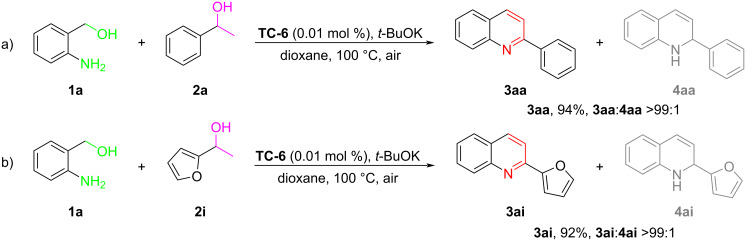
Gram-scale transformations.

The excellent developed methodology prompted us to further extend the practicality of the catalytic system. Firstly, we carried out a gram-scale reaction with the template reaction under the optimal catalytic system, which delivered quinoline **3aa** in 94% isolated yield (Figure 3a). Additionally, the 2-furanquoline product **3ai** was also obtained up to a gram-scale with excellent yield (92%) by iridium-catalyzed ADC reaction of 2-aminobenzyl alcohol **1a** and 2-furanol **2i** (Figure 3b).

**Figure 4:**
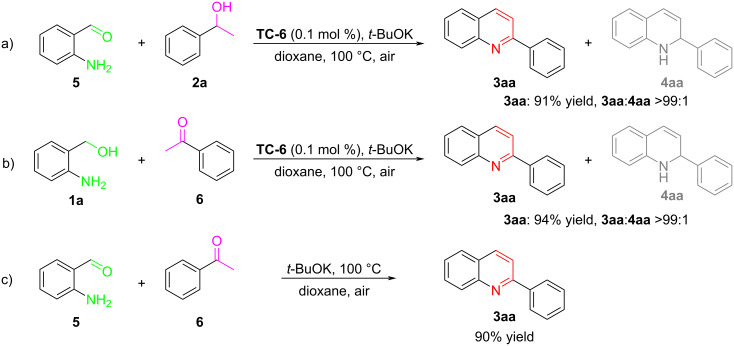
Mechanistic investigation.

To further stretch out the process of this cyclometalated iridium-catalyzed ADC reaction, comparative experiments were carried out. Quinoline **3aa** was obtained in 91% yield by ADC reaction between 2-aminobenzaldehyde (**5**) and 1-phenylethanol (**2a**) catalyzed by cyclometalated iridium **TC-6** (Figure 4a). In the same way, quinoline **3aa** could also be synthesized from 2-aminobenzyl alcohol (**1a**) and acetophenone (**6**) with **TC-6** as the catalyst (Figure 4b). Further study found that quinoline **3aa** could be obtained by the condensation reaction of 2-aminobenzaldehyde (**5**) with acetophenone (**6**) in the absence of cyclometalated iridium (Figure 4c).

**Figure 5:**
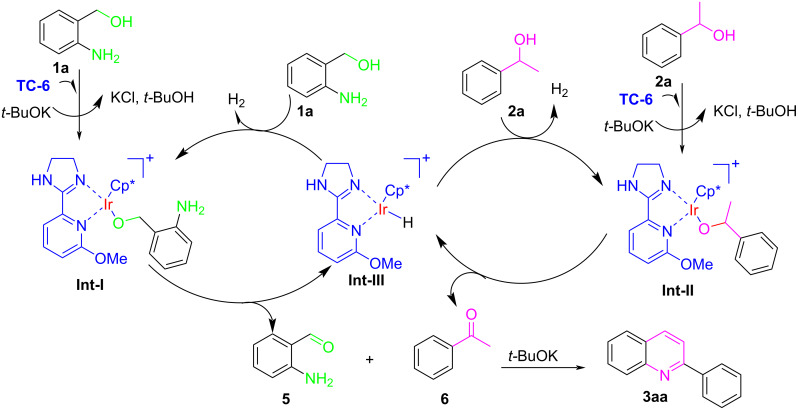
A speculated possible mechanism.

According to experimental results and literature findings [19,28–29,43–44], a possible mechanism of cyclometalated iridium-catalyzed ADC reaction was proposed (Figure 5). Firstly, by the interaction of **TC-6** with **1a**/**2a** under the “dehydrogenative” process, the **Int-I/Int-II** were formed [28–29]. Then, **Int-III** and 2-aminobenzaldehyde (**5**)/acetophenone (**6**) were formed by β-H elimination of **Int-I/Int-II**. In this process, an amount of liberated H_2_ would be released from the dehydrogenation of 2-aminobenzyl alcohol/1-phenylethanol according to the previous literature [28]. Lastly, the desired product **3aa** was obtained by the condensation and cyclization of the aldehyde **5** with acetophenone (**6**) under base conditions.

The potential antimicrobial activity of the compounds was evaluated against *Staphylococcus aureus* (Gram-positive), *Escherichia coli* (Gram-negative), and *Candida albicans* (fungi) mainly by examining the minimum inhibitory concentration (MIC) (Table 4). As shown in Table 4, the compounds **3ab**, **3ah**, and **3ad** showed high antibacterial activities against Gram-positive bacteria. In particular, the antibacterial activity of compound **3ad** against *Staphylococcus aureus* (MIC = 2 μg/mL) was much higher than that of the positive control norfloxacin. Meanwhile, the antifungal activity of compound **3ck** (MIC = 64 μg/mL) was stronger than norfloxacin. However, **3an** and other compounds showed similar or lower antifungal activity than norfloxacin. Unfortunately, all compounds were less effective against Gram-negative bacteria (MIC > 128 μg/mL) than the parent drug norfloxacin. To sum up, the synthesized compounds exhibited enhanced antibacterial activity against Gram-positive bacteria and *Candida albicans*.

**Table 4:** Results of antimicrobial activity of synthetic quinoline compounds.

Compounds	Minimum inhibitory concentration (μg/mL)					

	*C. albicans*		*S. aureus*		*E. coli*	

	predicted	experimental	predicted	experimental	predicted	experimental

**3ab**	>128	>128	**128**	**16**	128	>128
**3ad**	>128	>128	**128**	**2**	128	>128
**3ah**	>128	>128	**128**	**64**	128	>128
**3ai**	>128	128	128	>128	128	>128
**3aj**	>128	128	128	>128	128	>128
**3bj**	>128	128	128	>128	128	>128
**3ak**	>128	128	128	>128	128	>128
**3ck**	**>128**	**64**	128	>128	128	>128
**3an**	>128	128	128	>128	128	>128
norfloxacin	128		128		128	

## Conclusion

In summary, we have developed a new route for the efficient synthesis of quinoxaline and its derivatives with high yield and good chemoselectivity via the ADC reaction of 2-aminobenzyl alcohol and aryl aryl/heteroaryl/alkyl secondary alcohols including electron-donating (Me, OMe) and electron-withdrawing substituents (F, Cl, Br) catalyzed by cyclometalated iridium complexes. Besides, this reaction could also be used on a gram-scale, by which the aryl/heteroaryl quinolines were synthesized. In the evaluation of antimicrobial activity, the antimicrobial effects of compounds **3ab**, **3ad**, **3ah**, **3ck**, **3an** and other compounds were better than the parent drug norfloxacin. This method could be used to further synthesis of quinoline derivatives and provide theoretical support for the synthesis of new antibacterial drugs.

## Supporting Information

File 1 Experimental procedures, characterization data, copies of ^1^H and ^13^C NMR spectra, HRMS of new compounds.
