# Supplementary material for: Cyclometalated iridium complexes-catalyzed acceptorless dehydrogenative coupling reaction: construction of quinoline derivatives and evaluation of their antimicrobial activities
Source: Beilstein J Org Chem. 2022 Oct 27;18:1507–17. doi: 10.3762/bjoc.18.159 (PMC9623133; doi:10.3762/bjoc.18.159)

BEILSTEIN JOURNAL OF ORGANIC CHEMISTRY

Supporting Information

for

Cyclometalated iridium complexes-catalyzed acceptorless dehydrogenative coupling reaction: construction of quinoline derivatives and evaluation of their antimicrobial activities

Hongling Shui, Yuhong Zhong, Renshi Luo, Zhanyi Zhang, Jiuzhong Huang, Ping Yang and Nianhua Luo

Beilstein J. Org. Chem. **2022,** *18,* 1507–1517. doi:10.3762/bjoc.18.159

Experimental procedures, characterization data, copies of ^{1}H and ^{13}C NMR spectra, HRMS of new compounds

License and Terms: This is a supporting information file under the terms of the Creative Commons Attribution License (https://creativecommons.org/licenses/by/4.0). Please note that the reuse, redistribution and reproduction in particular requires that the author(s) and source are credited and that individual graphics may be subject to special legal provisions.
The license is subject to the *Beilstein Journal of Organic Chemistry* terms and conditions: (https://www.beilstein-journals.org/bjoc/terms)

Table of contents

1. General information S2

2. General procedure for the synthesis of **3** S2

3. Procedure for gram-scale ADC reaction S2

4. General procedure for antimicrobial activity S2

5. Characterization data S3

6. The ^{1}H and ^{13}C NMR spectra of compounds S10

7. HRMS of new compounds S54

1. General information

If not specified, all reagents were purchased from the reagent company and used directly in the reaction. The progress of the reaction was monitored by thin layer chromatography (TLC) or using an Agilent GC-7900. The reaction products were purified by column chromatography (200–300 mesh) or thin-plate chromatography and characterized by ^{1}H and ^{13}C nuclear magnetic resonance (NMR) spectra which were recorded on a Bruker AVANCE NEO 400 MHz NMR spectrometer. The melting points were determined using a WRR melting point apparatus. Chemical shifts (δ) are reported in ppm from internal tetramethylsilane or the central $CDCl_3$ resonance (δ = 7.26 ppm) for ^{1}H NMR and relative to the central $CDCl_3$ resonance (δ = 77.16 ppm) for ^{13}C NMR spectroscopy. Optical density (OD) of the bacterial suspension was recorded at 600 and 530 nm using a microplate reader (Multiskan GO, Thermo Scientific, Waltham, MA, USA).

2. General procedure for the synthesis of compounds 3

The synthesis process of **3** is similar to reference[1].

3. Procedure for gram-scale ADC reaction

The procedure for gram-scale ADC reaction is similar to reference[1].

4. General procedure for antibacterial and antifungal activity

The MIC values of the synthetic compounds against *S. aureus* (ATCC 6538P), *E. coli* (ATCC 8739), and *C. albicans* (ATCC 10231) were determined by the standard broth microdilution method. First, a 256 μg/mL solution of the test compound in 1% DMSO was prepared as the mother solution. This was added into the first column of a 96-well plate (100 μL per well), and then serially diluted in the subsequent columns. Then, 100 μL bacterial/fungal suspension (10^5 CFU/mL) was added to each well. The final concentrations of the test compounds obtained were 128, 64, 32, 16, 8, 4, 2, 1, 0.5, 0.25, and 0.125 μg/mL. After culturing at 37 ºC for 24 h, the OD_{600} (OD_{530} for fungi) was measured using the microplate reader. The control group included the blank contrast and negative contrast, which contained 200 μL of media and 200 μL of bacterial solution, separately. Norfloxacin was used as the positive control.

5. Characterization data

2-Phenylquinoline (3aa): [2] colorless oil. Yield: 196.9 mg, 96%. ^{1}H NMR (400 MHz, Chloroform-*d*) δ 8.20 – 8.11 (m, 4H), 7.82 – 7.66 (m, 3H), 7.53 – 7.41 (m, 4H). ^{13}C NMR (101 MHz, Chloroform-*d*) δ 157.39, 148.33, 139.73, 136.83, 129.79, 129.72, 129.39, 128.91, 127.64, 127.54, 127.23, 126.34, 119.05.

2-(4-Methoxyphenyl)-3-methylquinoline (3ab): [2] colorless oil. Yield: 223.3 mg, 95%. ^{1}H NMR (400 MHz, Chloroform-*d*) δ 8.11 (d, *J* = 8.4 Hz, 1H), 7.98 (s, 1H), 7.75 (d, *J* = 8.1 Hz, 1H), 7.64 (t, *J* = 7.5 Hz, 1H), 7.56 (d, *J* = 8.3 Hz, 2H), 7.48 (t, *J* = 7.3 Hz, 1H), 7.01 (d, *J* = 6.8 Hz, 2H), 3.86 (s, 3H), 2.47 (s, 3H).^{13}C NMR (101 MHz, Chloroform-*d*) δ 160.14, 159.68, 146.70, 136.77, 133.41, 130.33, 129.31, 129.21, 128.69, 127.46, 126.70, 126.24, 113.73, 55.40, 20.86.

2-(4-Methoxyphenyl)-3,8-dimethylquinoline (3bb): yellow solid, mp 102-103 ℃. Yield: 241.9 mg, 92%. ^{1}H NMR (400 MHz, Chloroform-*d*) δ 7.86 (s, 1H), 7.64 (d, *J* = 8.7 Hz, 2H), 7.53 (d, *J* = 8.1 Hz, 1H), 7.45 (d, *J* = 6.9 Hz, 1H), 7.33 (t, *J* = 7.6 Hz, 1H), 6.98 (d, *J* = 8.7 Hz, 2H), 3.82 (s, 3H), 2.80 (s, 3H), 2.47 (s, 3H). ^{13}C NMR (101 MHz, Chloroform-*d*) δ 159.72, 158.31, 145.92, 137.24, 137.18, 133.95, 130.89, 128.74, 128.72, 127.33, 125.98, 124.71, 113.57, 55.38, 21.01, 18.06. HRMS-ESI (m/z) calcd for $C_{18}H_{18}NO$, [M +H]$^+$: 264.1388; found: 264.1397.

6-Chloro-2-(4-methoxyphenyl)-3-methylquinoline (3cb): [3] yellow solid, mp 132-134 ℃. Yield: 263.1 mg, 93%. ^{1}H NMR (400 MHz, Chloroform-*d*) δ 8.01 (d, *J* = 8.9 Hz, 1H), 7.82 (s, 1H), 7.67 (s, 1H), 7.54 (d, *J* = 8.4 Hz, 3H), 7.00 (d, *J* = 8.6 Hz, 2H), 3.84 (s, 3H), 2.44 (s, 3H). ^{13}C NMR (101 MHz, Chloroform-*d*) δ 160.34, 159.84, 145.04, 135.79, 132.95, 131.76, 130.84, 130.39, 130.33, 129.54, 127.98, 125.33, 113.78, 55.38, 20.89.

6,8-Dibromo-2-(4-methoxyphenyl)-3-methylquinoline (3db): [4] yellow solid, mp 139-141 ℃. Yield: 367.6mg, 91%. ^{1}H NMR (400 MHz, Chloroform-*d*) δ 8.07 (d, *J* = 2.0 Hz, 1H), 7.87 (d, *J* = 2.0 Hz, 2H), 7.72 (d, *J* = 8.5 Hz, 2H), 7.03 (d, *J* = 8.6 Hz, 2H), 3.89 (s, 3H), 2.58 (s, 3H). ^{13}C NMR (101 MHz, Chloroform-*d*) δ 160.58, 160.18, 142.55, 136.41, 134.90, 132.49, 131.27, 131.02, 128.95, 128.52, 126.01, 119.09, 113.69, 55.40, 21.03.

2-(4-Chlorophenyl)-3-ethylquinoline (3ac): [4] yellow solid, mp 44-46 ℃. Yield: 259.8 mg, 94%. ^{1}H NMR (400 MHz, Chloroform-*d*) δ 8.11 (d, *J* = 8.5 Hz, 1H), 8.00 (s, 1H), 7.77 (d, *J* = 8.1 Hz, 1H), 7.64 (t, *J* = 7.6 Hz, 1H), 7.52 – 7.40 (m, 5H), 2.75 (q, *J* = 7.5 Hz, 2H), 1.16 (t, *J* = 7.5 Hz, 3H). ^{13}C NMR (101 MHz, Chloroform-*d*) δ 159.30, 146.40, 139.38, 135.20, 135.09, 134.25, 130.28, 129.25, 129.02, 128.55, 127.82, 127.04, 126.63, 26.01, 14.79.

2-(4-Chlorophenyl)-3-ethyl-8-methylquinoline (3bc): yellow solid, mp 59-61 ℃. Yield: 266.5 mg, 95%. ^{1}H NMR (400 MHz, Chloroform-*d*) δ 7.96 (s, 1H), 7.57 (dd, *J* = 20.9, 8.2 Hz, 3H), 7.50 – 7.34 (m, 4H), 2.79 (d, *J* = 8.5 Hz, 5H), 1.16 (t, *J* = 7.5 Hz, 3H). ^{13}C NMR (101 MHz, Chloroform-*d*) δ 157.65, 145.64, 139.89, 137.34, 135.49, 134.61, 134.15, 130.73, 129.01, 128.38, 127.70, 126.39, 124.99, 26.03, 18.01, 14.94. HRMS-ESI (m/z) calcd for $C_{18}H_{17}ClN$ [M +H]$^+$: 282.1050; found: 282.1056.

6-Chloro-2-(4-chlorophenyl)-3-ethylquinoline (3cc): yellow solid, mp 82-84 ℃. Yield: 274.8 mg, 91%. ^{1}H NMR (400 MHz, Chloroform-*d*) δ 8.02 (d, *J* = 8.9 Hz, 1H), 7.94 (s, 1H), 7.77 (s, 1H), 7.58 (d, *J* = 8.6 Hz, 1H), 7.51 – 7.42 (m, 4H), 2.77 (q, *J* = 7.5 Hz, 2H), 1.19 (t, *J* = 7.5 Hz, 3H). ^{13}C NMR (101 MHz, Chloroform-*d*) δ 159.58, 144.71, 138.93, 136.17, 134.47, 134.22, 132.26, 130.86, 130.18, 129.90, 128.61, 128.36, 125.64, 26.00, 14.63. HRMS-ESI (m/z) calcd for $C_{17}H_{14}Cl_2N$ [M +H]$^+$: 302.0503; found: 302.0516.

2-(4-Chlorophenyl)-3-propylquinoline (3ad): colorless oil. Yield: 257.3 mg, 93%. ^{1}H NMR (400 MHz, Chloroform-*d*) δ 8.10 (d, *J* = 8.5 Hz, 1H), 8.04 (s, 1H), 7.80 (d, *J* = 8.5 Hz, 1H), 7.67 (t, *J* = 7.5 Hz, 1H), 7.50 (dq, *J* = 15.7, 8.5, 8.0 Hz, 5H), 2.79 – 2.69 (m, 2H), 1.57 (q, *J* = 7.6 Hz, 2H), 0.87 (t, *J* = 7.4 Hz, 3H). ^{13}C NMR (101 MHz, Chloroform-*d*) δ 159.45, 146.39, 139.42, 136.01, 134.20, 133.64, 130.25, 129.23, 129.03, 128.54, 127.69, 126.98, 126.62, 34.88, 23.75, 13.93. HRMS-ESI (m/z) calcd for $C_{18}H_{17}ClN$ [M +H]$^+$: 282.1050; found: 282.1056.

2-(4-Chlorophenyl)-8-methyl-3-propylquinoline (3bd): colorless oil. Yield: 277.3 mg, 95%. ^{1}H NMR (400 MHz, Chloroform-*d*) δ 7.97 (s, 1H), 7.61 (d, *J* = 8.1 Hz, 1H), 7.56 (d, *J* = 8.3 Hz, 2H), 7.51 – 7.35 (m, 4H), 2.77 (d, *J* = 5.0 Hz, 5H), 1.56 (q, *J* = 7.6 Hz, 2H), 0.86 (t, *J* = 7.3 Hz, 3H). ^{13}C NMR (101 MHz, Chloroform-*d*) δ 157.77, 145.64, 139.95, 137.31, 136.25, 134.08, 133.12, 130.70, 128.97, 128.34, 127.55, 126.35, 124.92, 34.93, 23.86, 17.93, 13.97. HRMS-ESI (m/z) calcd for $C_{19}H_{19}ClN$ [M +H]$^+$: 296.1206; found: 296.1202

6-Chloro-2-(4-chlorophenyl)-3-propylquinoline (3cd): yellow solid, mp 121-123 ℃. Yield: 286.6 mg, 91%. ^{1}H NMR (400 MHz, Chloroform-*d*) δ 8.01 (d, *J* = 9.0 Hz, 1H), 7.92 (s, 1H), 7.76 (d, *J* = 2.1 Hz, 1H), 7.57 (dd, *J* = 9.0, 2.3 Hz, 1H), 7.52 – 7.40 (m, 4H), 2.85 – 2.58 (m, 2H), 1.55 (q, *J* = 7.6 Hz, 2H), 0.87 (t, *J* = 7.3 Hz, 3H). ^{13}C NMR (101 MHz, Chloroform-*d*) δ 159.68, 144.76, 139.03, 134.99, 134.71, 134.43, 132.26, 130.87, 130.20, 129.91, 128.59, 128.24, 125.62, 34.86, 23.64, 13.89. HRMS-ESI (m/z) calcd for $C_{18}H_{16}Cl_2N$ [M +H]$^+$: 316.0662; found: 316.0669.

6,8-Dibromo-2-(4-chlorophenyl)-3-propylquinoline (3dd): yellow solid, mp 112-114 ℃. Yield:403.8mg,92%.^{1}H NMR (400 MHz, Chloroform-*d*) δ 8.00 (d, *J* = 2.1 Hz, 1H), 7.84 – 7.76 (m, 2H), 7.55 (d, *J* = 8.3 Hz, 2H), 7.41 (d, *J* = 8.5 Hz, 2H), 2.80 – 2.65 (m, 2H), 1.52 (q, *J* = 7.5 Hz, 2H), 0.84 (t, *J* = 7.3 Hz, 3H). ^{13}C NMR (101 MHz, Chloroform-*d*) δ 160.02, 142.20, 138.53, 135.67, 135.43, 135.07, 134.72, 130.72, 129.19, 128.85, 128.48, 126.08, 119.58, 34.81, 23.65, 13.99. HRMS-ESI (m/z) calcd for $C_{18}H_{15}Br_2ClN$ [M +H]$^+$: 437.9260; found: 437.9247.

2-(4-Bromophenyl)-3-propylquinoline (3ae): yellow solid, mp 82-84 ℃. Yield: 302.2 mg, 93%. ^{1}H NMR (400 MHz, Chloroform-*d*) δ 8.10 (d, *J* = 8.4 Hz, 1H), 8.02 (s, 1H), 7.79 (d, *J* = 8.1 Hz, 1H), 7.64 (dd, *J* = 23.8, 7.8 Hz, 3H), 7.55 – 7.48 (m, 1H), 7.43 (d, *J* = 8.1 Hz, 2H), 2.78 – 2.66 (m, 2H), 1.63 – 1.48 (m, 2H), 0.87 (t, *J* = 7.3 Hz, 3H). ^{13}C NMR (101 MHz, Chloroform-*d*) δ 159.45, 146.42, 139.91, 136.02, 133.58, 131.49, 130.57, 129.25, 129.05, 127.69, 127.01, 126.64, 122.46, 34.87, 23.77, 13.95. HRMS-ESI (m/z) calcd for $C_{18}H_{17}BrN$ [M +H]$^+$: 326.0544; found: 326.0548.

2-(4-Bromophenyl)-8-methyl-3-propylquinoline (3be): yellow oil. Yield: 322 mg, 95%. ^{1}H NMR (400 MHz, Chloroform-*d*) δ 7.95 (s, 1H), 7.59 (d, *J* = 8.2 Hz, 3H), 7.48 (d, *J* = 8.5 Hz, 3H), 7.38 (t, *J* = 7.5 Hz, 1H), 2.87 – 2.62 (m, 5H), 1.55 (dt, *J* = 15.3, 7.5 Hz, 2H), 0.85 (t, *J* = 7.3 Hz, 3H). ^{13}C NMR (101 MHz, Chloroform-*d*) δ 157.79, 145.67, 140.44, 137.34, 136.28, 133.09, 131.31, 131.05, 129.01, 127.57, 126.40, 124.97, 122.39, 34.94, 23.91, 18.00, 14.03. HRMS-ESI (m/z) calcd for $C_{19}H_{19}BrN$ [M +H]$^{+}$: 340.0701; found: 340.0703.

2-(4-Bromophenyl)-6-chloro-3-propylquinoline (3ce): yellow solid, mp 129-131 ℃. Yield: 323.1mg, 90%. ^{1}H NMR (400 MHz, Chloroform-*d*) δ 8.02 (d, *J* = 9.0 Hz, 1H), 7.93 (s, 1H), 7.76 (d, *J* = 2.3 Hz, 1H), 7.65 – 7.54 (m, 3H), 7.42 (d, *J* = 8.1 Hz, 2H), 2.79 – 2.65 (m, 2H), 1.56 (q, *J* = 7.6 Hz, 2H), 0.87 (t, *J* = 7.3 Hz, 3H). ^{13}C NMR (101 MHz, Chloroform-*d*) δ 159.70, 144.75, 139.48, 135.02, 134.66, 132.27, 131.55, 130.87, 130.49, 129.94, 128.24, 125.63, 122.68, 34.84, 23.65, 13.92. HRMS-ESI (m/z) calcd for $C_{18}H_{16}BrClN$ [M +H]$^{+}$: 360.0154; found: 360.0157.

6,8-Dibromo-2-(4-bromophenyl)-3-propylquinoline (3de): yellow solid, mp 116-118 ℃. Yield: 439.5 mg, 91%. ^{1}H NMR (400 MHz, Chloroform-*d*) δ 8.03 (d, *J* = 2.0 Hz, 1H), 7.84 (d, *J* = 7.7 Hz, 2H), 7.58 (d, *J* = 8.2 Hz, 2H), 7.49 (d, *J* = 8.2 Hz, 2H), 2.83 – 2.67 (m, 2H), 1.52 (dt, *J* = 14.9, 7.5 Hz, 2H), 0.85 (t, *J* = 7.3 Hz, 3H). ^{13}C NMR (101 MHz, Chloroform-*d*) δ 160.07, 142.23, 138.97, 135.63, 135.46, 135.12, 131.45, 131.00, 129.21, 128.86, 126.07, 123.07, 119.62, 34.79, 23.67, 13.99. HRMS-ESI (m/z) calcd for $C_{18}H_{15}Br_3N$ [M +H]$^{+}$: 481.8754; found: 481.8766.

2-(4-Fluorophenyl)-3-propylquinoline (3af): colorless oil. Yield: 233.2 mg, 88%. ^{1}H NMR (400 MHz, Chloroform-*d*) δ 8.11 (d, *J* = 8.4 Hz, 1H), 8.03 (s, 1H), 7.80 (d, *J* = 8.1 Hz, 1H), 7.67 (t, *J* = 7.6 Hz, 1H), 7.53 (dd, *J* = 8.4, 4.8 Hz, 3H), 7.17 (t, *J* = 8.6 Hz, 2H), 2.77 – 2.69 (m, 2H), 1.56 (h, *J* = 7.4 Hz, 2H), 0.87 (t, *J* = 7.3 Hz, 3H). ^{13}C NMR (101 MHz, Chloroform-*d*) δ163.95, 161.50, 159.66, 146.37, 137.03 (d, *J* = 3.3 Hz), 135.93, 133.75, 130.62 (d, *J* = 8.2 Hz), 129.20, 128.96, 127.65, 126.96, 126.52, 115.39, 115.17, 34.95, 23.72, 13.93. HRMS-ESI (m/z) calcd for $C_{18}H_{17}FN$ [M +H]$^{+}$: 266.1345; found: 266.1356.

2-(4-Fluorophenyl)-8-methyl-3-propylquinoline (3bf): colorless oil. Yield: 259.4 mg, 93%. ^{1}H NMR (400 MHz, Chloroform-*d*) δ 7.96 (s, 1H), 7.60 (t, *J* = 7.0 Hz, 3H), 7.49 (d, *J* = 7.0 Hz, 1H), 7.38 (t, *J* = 7.5 Hz, 1H), 7.15 (t, *J* = 8.6 Hz, 2H), 2.78 (d, *J* = 7.3 Hz, 5H), 1.55 (q, *J* = 7.6 Hz, 2H), 0.86 (t, *J* = 7.4 Hz, 3H). ^{13}C NMR (101 MHz, Chloroform-*d*) δ163.93, 161.48, 157.95, 145.57, 137.50 (d, *J* = 3.3 Hz), 137.24, 136.1, 133.19, 131.05 (d, *J* = 8.1 Hz), 128.90, 127.47, 126.22, 124.89, 115.15, 114.94, 34.99, 23.82, 17.94, 13.97. HRMS-ESI (m/z) calcd for $C_{19}H_{19}FN$ [M +H]$^{+}$: 280.1502; found: 280.1498.

6-Chloro-2-(4-fluorophenyl)-3-propylquinoline (3cf): yellow solid, mp 91-93 ℃. Yield: 284.1 mg, 95%. ^{1}H NMR (400 MHz, Chloroform-*d*) δ 8.04 (d, *J* = 8.9 Hz, 1H), 7.95 (s, 1H), 7.79 (s, 1H), 7.60 (d, *J* = 9.0 Hz, 1H), 7.53 (dd, *J* = 8.2, 5.5 Hz, 2H), 7.18 (t, *J* = 8.6 Hz, 2H), 2.79 – 2.70 (m, 2H), 1.56 (q, *J* = 7.6 Hz, 2H), 0.87 (t, *J* = 7.3 Hz, 3H). ^{13}C NMR (101 MHz, Chloroform-*d*) δ161.61, 159.90, 144.68, 134.93 (d, *J* = 16.5 Hz), 132.22, 130.99 – 130.46 (m), 129.92, 128.23, 125.60, 115.48, 115.27, 34.92, 23.60, 13.87. HRMS-ESI (m/z) calcd for $C_{18}H_{16}ClFN$ [M +H]$^{+}$: 300.0955; found: 300.0951.

6,8-Dibromo-2-(4-fluorophenyl)-3-propylquinoline (3df): yellow solid, mp 99-101 ℃. Yield: 372.2 mg, 88%. ^{1}H NMR (400 MHz, Chloroform-*d*) δ 8.06 (d, *J* = 2.0 Hz, 1H), 7.88 (d, *J* = 4.2 Hz, 2H), 7.63 (dd, *J* = 8.5, 5.5 Hz, 2H), 7.17 (t, *J* = 8.6 Hz, 2H), 2.84 – 2.76 (m, 2H), 1.55 (q, *J* = 7.6 Hz, 2H), 0.87 (t, *J* = 7.3 Hz, 3H). ^{13}C NMR (101 MHz, Chloroform-*d*) δ164.27, 161.80, 160.26, 142.24, 136.15 (d, *J* = 3.4 Hz), 135.74, 135.42, 135.09, 131.18 (d, *J* = 8.3 Hz), 129.19, 128.81, 126.00, 119.48, 115.38, 115.17, 34.86, 23.63, 13.92. HRMS-ESI (m/z) calcd for $C_{18}H_{15}Br_2FN$ [M +H]$^+$: 421.9555; found: 421.9582.

2-(2,4-Dichlorophenyl)-3-propylquinoline (3ag): yellow oil. Yield: 283.5 mg, 90%. ^{1}H NMR (400 MHz, Chloroform-*d*) δ 8.21 – 7.96 (m, 2H), 7.83 (d, *J* = 8.1 Hz, 1H), 7.69 (t, *J* = 7.6 Hz, 1H), 7.60 – 7.47 (m, 2H), 7.44 – 7.29 (m, 2H), 2.89 – 2.26 (m, 2H), 1.66 – 1.49 (m, 2H), 0.87 (t, *J* = 7.3 Hz, 3H). ^{13}C NMR (101 MHz, Chloroform-*d*) δ 157.64, 146.29, 138.23, 135.49, 134.73, 134.29, 133.79, 131.41, 129.45, 129.25, 129.06, 128.03, 127.30, 127.11, 126.91, 34.31, 23.20, 13.88. HRMS-ESI (m/z) calcd for $C_{18}H_{16}Cl_2N$ [M +H]$^+$: 316.0660; found: 316.0669.

2-(2,4-Dichlorophenyl)-8-methyl-3-propylquinoline (3bg): colorless oil. Yield: 305.9 mg, 93%. ^{1}H NMR (400 MHz, Chloroform-*d*) δ 7.98 (s, 1H), 7.64 (d, *J* = 8.0 Hz, 1H), 7.50 (d, *J* = 7.5 Hz, 2H), 7.42 (t, *J* = 7.6 Hz, 1H), 7.38 – 7.31 (m, 2H), 2.75 (s, 3H), 2.57 (d, *J* = 36.0 Hz, 2H), 1.54 (d, *J* = 9.3 Hz, 2H), 0.84 (t, *J* = 7.3 Hz, 3H). ^{13}C NMR (101 MHz, Chloroform-*d*) δ 157.64, 146.29, 138.23, 135.49, 134.73, 134.29, 133.79, 131.41, 129.45, 129.25, 129.06, 128.03, 127.30, 127.11, 126.91, 34.31, 23.20, 13.88. HRMS-ESI (m/z) calcd for $C_{19}H_{18}Cl_2N$ [M +H]$^+$: 330.0816; found: 330.0801.

6-Chloro-2-(2,4-dichlorophenyl)-3-propylquinoline (3cg): yellow solid, mp 92-94 ℃. Yield: 310.6 mg, 89%. ^{1}H NMR (400 MHz, Chloroform-*d*) δ 8.02 (d, *J* = 8.9 Hz, 1H), 7.94 (s, 1H), 7.77 (d, *J* = 2.3 Hz, 1H), 7.69 – 7.40 (m, 2H), 7.35 (q, *J* = 8.4 Hz, 2H), 2.56 (dt, *J* = 34.5, 7.8 Hz, 2H), 1.55 (dd, *J* = 7.6, 3.7 Hz, 2H), 0.85 (t, *J* = 7.3 Hz, 3H). ^{13}C NMR (101 MHz, Chloroform-*d*) δ 157.93, 144.64, 137.87, 135.38, 134.91, 134.47, 133.71, 132.61, 131.33, 130.88, 129.93, 129.48, 128.61, 127.36, 125.75, 34.28, 23.09, 13.88. HRMS-ESI (m/z) calcd for $C_{18}H_{15}Cl_3N$ [M +H]$^+$: 350.0270; found: 350.0256.

6,8-Dibromo-2-(2,4-dichlorophenyl)-3-propylquinoline (3dg): yellow oil. Yield: 430.4 mg, 91%. ^{1}H NMR (400 MHz, Chloroform-*d*) δ 8.05 (s, 1H), 7.90 (d, *J* = 7.3 Hz, 2H), 7.47 (s, 1H), 7.33 (d, *J* = 9.6 Hz, 2H), 2.57 (d, *J* = 40.0 Hz, 2H), 1.58 – 1.44 (m, 2H), 0.81 (t, *J* = 7.3 Hz, 3H). ^{13}C NMR (101 MHz, Chloroform-*d*) δ 158.75, 142.28, 137.63, 136.51, 135.19, 134.97, 134.77, 133.69, 131.68, 129.76, 129.43, 129.04, 127.34, 126.07, 119.99, 34.16, 23.12, 13.90. HRMS-ESI (m/z) calcd for $C_{18}H_{14}Br_2Cl_2N$ [M +H]$^+$: 471.8870; found: 471.8889.

3-Benzyl-2-(furan-2-yl)quinoline (3ah): brown solid, mp 74-75 ℃. Yield: 253.7 mg, 89%. ^{1}H NMR (400 MHz, Chloroform-*d*) δ 8.14 (d, *J* = 7.5 Hz, 1H), 7.82 (s, 1H), 7.70 – 7.61 (m, 3H), 7.51 – 7.44 (m, 1H), 7.34 – 7.13 (m, 6H), 7.01 (s, 1H), 6.53 (s, 1H), 4.45 (s, 2H). ^{13}C NMR (101 MHz, Chloroform-*d*) δ 153.20, 148.97, 146.55, 143.69, 139.60, 138.01, 131.34, 129.44, 129.03, 128.69, 127.22, 127.08, 126.61, 126.43, 112.61, 111.76, 39.13. HRMS-ESI (m/z) calcd for $C_{20}H_{16}NO$ [M +H]$^+$: 286.1232; found: 286.1241.

2-(Furan-2-yl)quinoline (3ai): [5]yellow solid, mp 113-115 ℃.Yield: 183 mg, 94%. ^{1}H NMR (400 MHz, Chloroform-*d*) δ 8.13 (dd, *J* = 8.6, 3.4 Hz, 2H), 7.80 – 7.66 (m, 3H), 7.61 (s, 1H), 7.47 (t, *J* = 7.5 Hz, 1H), 7.21 (d, *J* = 3.5 Hz, 1H), 6.57 (s, 1H). ^{13}C NMR (101 MHz, Chloroform-*d*) δ 153.63, 148.99, 148.05, 144.16, 136.72, 129.91, 129.30, 127.59, 127.15, 126.23, 117.48, 112.25, 110.22.

2-(Furan-2-yl)-8-methylquinoline (3bi): [6]yellow solid, mp 27-29 ℃.Yield: 196.4 mg, 94%.^{1}H NMR (400 MHz, Chloroform-*d*) δ 8.09 (d, *J* = 8.5 Hz, 1H), 7.81 (d, *J* = 8.5 Hz, 1H), 7.63 – 7.48 (m, 3H), 7.35 (t, *J* = 7.5 Hz, 1H), 7.26 (d, *J* = 3.4 Hz, 1H), 6.56 (s, 1H), 2.84 (s, 3H). ^{13}C NMR (101 MHz, Chloroform-*d*) δ 154.58, 148.01, 147.14, 143.66, 137.21, 136.82, 129.88, 127.12, 125.88, 125.55, 116.88, 112.27, 109.52, 17.80.

6-Chloro-2-(furan-2-yl)quinoline (3ci): [5]yellow solid, mp 81-83 ℃.Yield: 210.6 mg, 92%. ^{1}H NMR (400 MHz, Chloroform-*d*) δ 7.98 (d, *J* = 8.9 Hz, 1H), 7.87 (d, *J* = 8.5 Hz, 1H), 7.68 – 7.53 (m, 4H), 7.19 – 7.10 (m, 1H), 6.53 (s, 1H). ^{13}C NMR (101 MHz, Chloroform-*d*) δ 153.31, 149.07, 146.36, 144.25, 135.54, 131.68, 130.80, 130.62, 127.53, 126.19, 118.13, 112.31, 110.47.

6,8-Dibromo-2-(furan-2-yl)quinoline (3di): yellow solid, mp 136-138 ℃. Yield: 350.1 mg, 90%.^{1}H NMR (400 MHz, Chloroform-*d*) δ 8.06 (s, 1H), 7.95 (d, *J* = 8.4 Hz, 1H), 7.79 (d, *J* = 8.5 Hz, 2H), 7.60 (s, 1H), 7.33 (s, 1H), 6.58 (s, 1H).^{13}C NMR (101 MHz, Chloroform-*d*) δ 153.32, 149.88, 144.55, 143.86, 136.09, 136.05, 129.42, 128.68, 125.77, 118.99, 118.68, 112.60, 111.43. HRMS-ESI (m/z) calcd for $C_{13}H_8Br_2NO$ [M +H]$^+$: 351.8972; found: 351.8964.

2-(5-Methylfuran-2-yl)quinoline (3aj): [2]yellow solid, mp 113-115 ℃. Yield: 235.2 mg, 96%. ^{1}H NMR (400 MHz, Chloroform-*d*) δ 8.11 (d, *J* = 8.4 Hz, 1H), 8.01 (d, *J* = 8.6 Hz, 1H), 7.72 – 7.59 (m, 3H), 7.39 (t, *J* = 7.4 Hz, 1H), 7.08 (d, *J* = 3.3 Hz, 1H), 6.14 (d, *J* = 3.1 Hz, 1H), 2.40 (s, 3H). ^{13}C NMR (101 MHz, Chloroform-*d*) δ 154.51, 152.17, 149.19, 148.14, 136.46, 129.70, 129.25, 127.54, 126.94, 125.84, 117.29, 111.55, 108.69, 14.04.

8-Methyl-2-(5-methylfuran-2-yl)quinoline (3bj): colorless oil. Yield: 240.8 mg, 93%. ^{1}H NMR (400 MHz, Chloroform-*d*) δ 8.05 (d, *J* = 8.6 Hz, 1H), 7.76 (d, *J* = 8.6 Hz, 1H), 7.53 (dd, *J* = 22.4, 7.5 Hz, 2H), 7.35 – 7.28 (m, 1H), 7.15 (d, *J* = 3.2 Hz, 1H), 6.16 (d, *J* = 2.4 Hz, 1H), 2.83 (s, 3H), 2.42 (s, 3H). ^{13}C NMR (101 MHz, Chloroform-*d*) δ 153.94, 152.96, 148.20, 147.19, 137.05, 136.66, 129.80, 126.90, 125.55, 125.52, 116.70, 110.79, 108.64, 17.78, 14.02. HRMS-ESI (m/z) calcd for $C_{15}H_{14}NO$ [M +H]$^+$: 224.1075; found: 224.1070.

6-Chloro-2-(5-methylfuran-2-yl)quinoline (3cj): yellow oil. Yield: 256.6 mg, 92%. ^{1}H NMR (400 MHz, Chloroform-*d*) δ 8.00 (dd, *J* = 11.1, 9.0 Hz, 2H), 7.77 – 7.68 (m, 2H), 7.59 (dd, *J* = 9.0, 2.3 Hz, 1H), 7.10 (d, *J* = 3.2 Hz, 1H), 6.18 (d, *J* = 2.8 Hz, 1H), 2.45 (s, 3H). ^{13}C NMR (101 MHz, Chloroform-*d*) δ 154.86, 151.81, 149.36, 146.52, 135.52, 131.40, 130.79, 130.57, 127.46, 126.23, 118.13, 111.94, 108.80, 14.06. HRMS-ESI (m/z) calcd for $C_{14}H_{11}ClNO$ [M +H]$^+$: 244.0529; found: 244.0527.

6,8-Dibromo-2-(5-methylfuran-2-yl)quinoline (3dj): colorless oil. Yield: 366.7 mg, 91%. ^{1}H NMR (400 MHz, Chloroform-*d*) δ 8.07 (d, *J* = 1.9 Hz, 1H), 7.94 (d, *J* = 8.7 Hz, 1H), 7.83 – 7.73 (m, 2H), 7.25 (d, *J* = 3.3 Hz, 1H), 6.19 (d, *J* = 2.6 Hz, 1H), 2.43 (s, 3H). ^{13}C NMR (101 MHz, Chloroform-*d*) δ 155.08, 151.76, 150.06, 143.97, 135.96,

135.93, 129.40, 128.51, 125.60, 118.53, 112.86, 109.13, 14.09. HRMS-ESI (m/z) calcd for $C_{14}H_{10}Br_2NO$ [M +H]$^+$: 365.9129; found: 365.9145.

3-Methyl-2-(5-methylfuran-2-yl)quinoline (3ak): colorless oil. Yield: 251.2 mg, 97%. ^{1}H NMR (400 MHz, Chloroform-*d*) δ 8.10 (d, *J* = 8.5 Hz, 1H), 7.84 (s, 1H), 7.68 – 7.55 (m, 2H), 7.40 (t, *J* = 7.5 Hz, 1H), 6.98 (d, *J* = 3.3 Hz, 1H), 6.16 (d, *J* = 3.3 Hz, 1H), 2.61 (s, 3H), 2.44 (s, 3H). ^{13}C NMR (101 MHz, Chloroform-*d*) δ 154.01, 151.91, 149.04, 146.55, 137.52, 129.11, 128.79, 128.01, 126.99, 126.58, 126.08, 114.00, 108.16, 21.45, 14.06. HRMS-ESI (m/z) calcd for $C_{15}H_{14}NO$ [M +H]$^+$: 224.1075; found: 224.1070.

3,8-Dimethyl-2-(5-methylfuran-2-yl)quinoline (3bk): colorless oil. Yield: 259.3 mg, 95%. ^{1}H NMR (400 MHz, Chloroform-*d*) δ 7.78 (s, 1H), 7.50 – 7.38 (m, 2H), 7.32 – 7.23 (m, 1H), 7.09 (d, *J* = 3.3 Hz, 1H), 6.14 (d, *J* = 2.7 Hz, 1H), 2.82 (s, 3H), 2.65 (s, 3H), 2.41 (s, 3H). ^{13}C NMR (101 MHz, Chloroform-*d*) δ 153.52, 147.81, 145.64, 137.75, 136.86, 128.86, 127.66, 127.04, 125.80, 124.61, 113.18, 108.11, 21.17, 17.78, 14.05. HRMS-ESI (m/z) calcd for $C_{16}H_{16}NO$ [M +H]$^+$: 238.1232; found: 238.1237.

6-Chloro-3-methyl-2-(5-methylfuran-2-yl)quinoline (3ck): yellow solid, mp 100-102 ℃. Yield: 272.4 mg, 93%. ^{1}H NMR (400 MHz, Chloroform-*d*) δ 8.01 (d, *J* = 9.0 Hz, 1H), 7.79 (s, 1H), 7.64 (s, 1H), 7.54 (dd, *J* = 9.0, 2.3 Hz, 1H), 7.01 (d, *J* = 3.4 Hz, 1H), 6.19 (d, *J* = 3.3 Hz, 1H), 2.65 (s, 3H), 2.46 (s, 3H). ^{13}C NMR (101 MHz, Chloroform-*d*) δ 154.34, 151.61, 149.21, 144.88, 136.53, 131.60, 130.67, 129.71, 129.05, 127.49, 125.24, 114.40, 108.29, 21.50, 14.06. HRMS-ESI (m/z) calcd for $C_{15}H_{13}ClNO$ [M +H]$^+$: 258.0685; found: 258.0686.

6,8-Dibromo-3-methyl-2-(5-methylfuran-2-yl)quinoline (3dk): yellow solid, mp 140-142 ℃. Yield: 379.4 mg, 91%. ^{1}H NMR (400 MHz, Chloroform-*d*) δ 8.00 (d, *J* = 2.1 Hz, 1H), 7.76 – 7.68 (m, 2H), 7.24 (d, *J* = 3.4 Hz, 1H), 6.19 (d, *J* = 3.3 Hz, 1H), 2.69 (s, 3H), 2.44 (s, 3H). ^{13}C NMR (101 MHz, Chloroform-*d*) δ 154.64, 152.49, 149.63, 142.27, 136.89, 134.98, 129.91, 128.45, 128.42, 125.45, 118.67, 115.14, 108.58, 21.12, 14.11. HRMS-ESI (m/z) calcd for $C_{15}H_{12}Br_2NO$ [M +H]$^+$: 379.9285; found: 379.9270.

2-(Thiophen-2-yl)quinoline (3al): [2] yellow solid, mp 127-129 ℃. Yield: 194.1 mg, 92%. ^{1}H NMR (400 MHz, Chloroform-*d*) δ 8.06 (d, *J* = 8.2 Hz, 1H), 7.94 (d, *J* = 8.3 Hz, 1H), 7.62 (d, *J* = 8.2 Hz, 4H), 7.37 (t, *J* = 7.4 Hz, 2H), 7.05 (s, 1H). ^{13}C NMR (101 MHz, Chloroform-*d*) δ 152.37, 148.16, 145.47, 136.64, 129.88, 129.27, 128.67, 128.19, 127.59, 126.14, 126.00, 117.67.

8-Methyl-2-(thiophen-2-yl)quinoline (3bl): colorless oil. Yield: 213.5 mg, 95%. ^{1}H NMR (400 MHz, Chloroform-*d*) δ 7.92 (d, *J* = 8.4 Hz, 1H), 7.61 (d, *J* = 8.9 Hz, 2H), 7.46 (t, *J* = 8.9 Hz, 2H), 7.35 (d, *J* = 4.5 Hz, 1H), 7.27 (t, *J* = 7.4 Hz, 1H), 7.05 (s, 1H), 2.84 – 2.79 (m, 3H). ^{13}C NMR (101 MHz, Chloroform-*d*) δ 151.04, 147.05, 146.39, 137.24, 136.81, 129.98, 128.52, 128.09, 127.15, 125.91, 125.49, 125.44, 117.06, 17.81. HRMS-ESI (m/z) calcd for $C_{14}H_{12}NS$ [M +H]$^+$: 226.0690; found: 226.0689.

6-Chloro-2-(thiophen-2-yl)quinoline (3cl): [5] yellow solid, mp 104-106 ℃. Yield: 225.4 mg, 92%. ^{1}H NMR (400 MHz, Chloroform-*d*) δ 7.89 (d, *J* = 8.6 Hz, 1H), 7.74 (d, *J* = 8.3 Hz, 1H), 7.52 (d, *J* = 8.4 Hz, 4H), 7.39 (d, *J* = 4.3 Hz, 1H), 7.03 (s, 1H). ^{13}C NMR (101 MHz, Chloroform-*d*) δ 152.45, 146.38, 144.95, 135.52, 131.60, 130.75, 130.59, 128.98, 128.22, 127.61, 126.21, 118.31.

2-(Pyridin-3-yl)quinoline (3am): [7] yellow solid, mp 57-59 ℃. Yield: 206.8 mg, 94%. ^{1}H NMR (400 MHz, Chloroform-*d*) δ 9.33 (d, *J* = 2.3 Hz, 1H), 8.67 (dd, *J* = 4.8, 1.7 Hz, 1H), 8.45 (d, *J* = 7.9 Hz, 1H), 8.16 (t, *J* = 9.0 Hz, 2H), 7.83 – 7.66 (m, 3H), 7.51 (t, *J* = 7.5 Hz, 1H), 7.40 (dd, *J* = 8.0, 4.8 Hz, 1H). ^{13}C NMR (101 MHz, Chloroform-*d*) δ 154.50, 150.16, 148.77, 148.30, 137.13, 135.04, 134.91, 129.98, 129.71, 127.56, 127.32, 126.77, 123.67, 118.45.

2-(6-Methylpyridin-2-yl)quinoline (3an): [8] yellow solid, mp 68-70 ℃. Yield: 197.7 mg, 96%. ^{1}H NMR (400 MHz, Chloroform-*d*) δ 8.58 (d, *J* = 8.6 Hz, 1H), 8.43 (d, *J* = 7.8 Hz, 1H), 8.20 (dd, *J* = 25.7, 8.5 Hz, 2H), 7.81 (d, *J* = 8.1 Hz, 1H), 7.71 (q, *J* = 7.5 Hz, 2H), 7.51 (t, *J* = 7.2 Hz, 1H), 7.18 (d, *J* = 7.6 Hz, 1H), 2.66 (s, 3H). ^{13}C NMR (101 MHz, Chloroform-*d*) δ 157.93, 156.56, 155.76, 147.97, 137.12, 136.69, 129.84, 129.47, 128.23, 127.63, 126.62, 123.60, 119.17, 118.85, 24.73.

2-Cyclohexyl-quinoline (3ao): [2] colorless oil, Yield: 200.6 mg, 88%. ^{1}H NMR (400 MHz, $CDCl_3$-*d*) δ 8.09 (t, *J* = 7.8 Hz, 2H), 7.79 (d, *J* = 8.0 Hz, 1H), 7.69 (t, *J* = 7.4 Hz, 1H), 7.49 (t, *J* = 7.2 Hz, 1H), 7.35 (d, *J* = 8.4 Hz, 1H), 2.95 (dd, *J* = 16.7, 7.0 Hz, 1H), 2.05 (d, *J* = 12.5 Hz, 2H), 1.92 (d, *J* = 12.4 Hz, 2H), 1.82 (d, *J* = 12.4 Hz, 1H), 1.66 (q, *J* = 12.4 Hz, 2H), 1.50 (dt, *J* = 25.5, 12.7 Hz, 2H), 1.36 (dd, *J* = 17.5, 7.4 Hz, 1H); ^{13}C NMR (101 MHz, $CDCl_3$-*d*) δ 166.8, 147.7, 136.4, 129.3, 128.9, 127.4, 126.9), 125.6, 119.6, 47.6, 32.9, 26.6, 26.1.

3-Propylquinoline (3ap): [7] colorless oil, Yield: 147.1 mg, 86%. ^{1}H NMR (400 MHz, $CDCl_3$-*d*) δ 8.74 (d, *J* = 1.7 Hz, 1H), 8.09 (d, *J* = 8.4 Hz, 1H), 7.83 (s, 1H), 7.69 (d, *J* = 8.1 Hz, 1H), 7.63 – 7.55 (m, 1H), 7.48 – 7.41 (m, 1H), 2.72 – 2.66 (m, 2H), 1.71 – 1.64 (m, 2H), 0.96 – 0.91 (m, 3H); ^{13}C NMR (101 MHz, $CDCl_3$-*d*) δ 151.90 (s), 146.55, 135.05, 134.28, 128.92, 128.50, 128.15, 127.25, 126.49, 35.13, 24.17, 13.64.

References

[1] Shui, H.-L.; Zhong, Y.-H.; Ouyang, L.; Luo, N.-H.; Luo, R.-S. *Synthesis*. **2022**, *54*, 2876.

[2] Luo, N.-H.; Shui, H.-L.; Zhong, Y.-H.; Huang, J.-Z.; Luo, R.-S. *Synthesis,* **2021**, *23*, 4516.

[3] Hu, X.; Chen, Y.-Q.; Huang, B.-B.; Liu, Y.-C.; Huang, H.-T.; Xie. Z.-L. *ACS Sustain. Chem. Eng.* **2019**, *7*, 11369.

[4] Togo, H.; Naruto, H. *Org. Biomol. Chem*. **2019**, ***17*,** 5760.

[5] Wang, P.; Wang, X.-W.; Niu, X.-Y.; Zhu, L.; Yao, X.-Q. *Chem. Comm*. **2020**, *56*, 4840.

[6] Bering, L.; Antonchick, A. P. *Org. Lett*. **2015**, *17*, 3134.

[7] Chakraborty, G.; Sikari, R.; Das, S.; Mondal, R.; Sinha, S.; Banerjee, S.; Paul, N. D. *J. Org. Chem*. **2019**, *84*, 2626.

[8 Liu, Y.-R.; Chen., F.; He, Y.-M.; Li, C.-H.; Fan, Q.-H. *Org. Biomol. Chem*. **2019**, *17*, 5099.

6. The ^{1}H and ^{13}C NMR spectra of compounds

^{1}H NMR spectrum of 2-phenylquinoline (3aa)

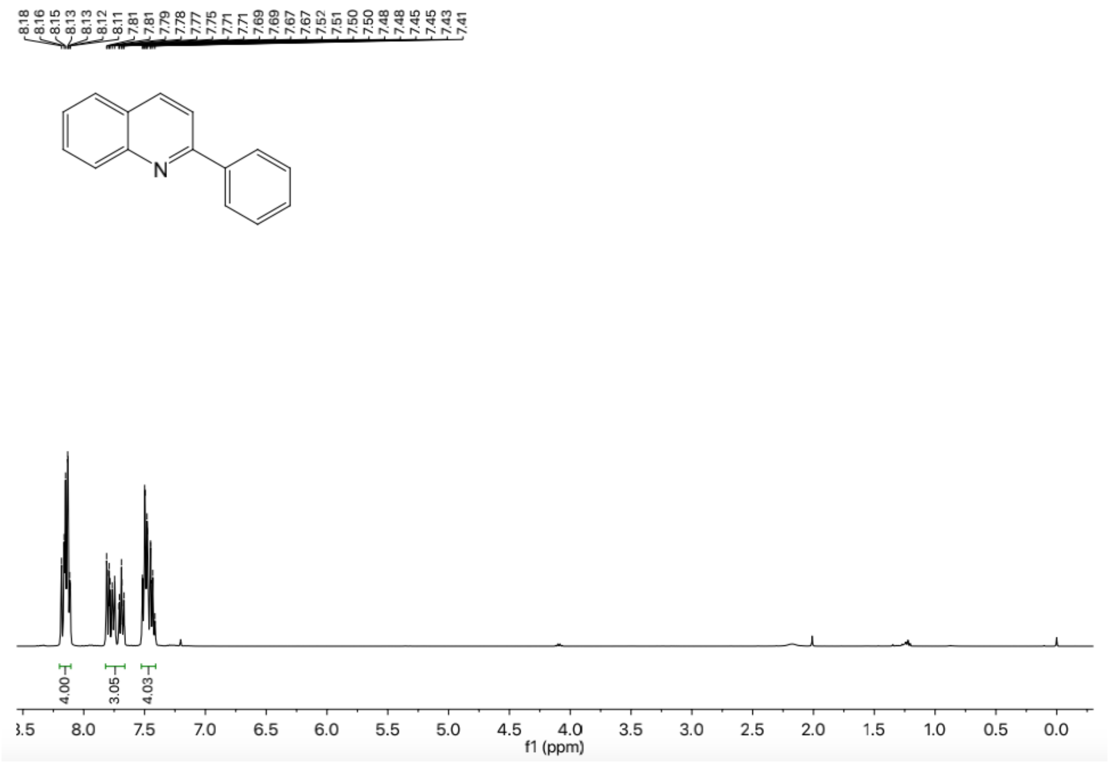

^{13}C NMR spectrum of 2-phenylquinoline (3aa)

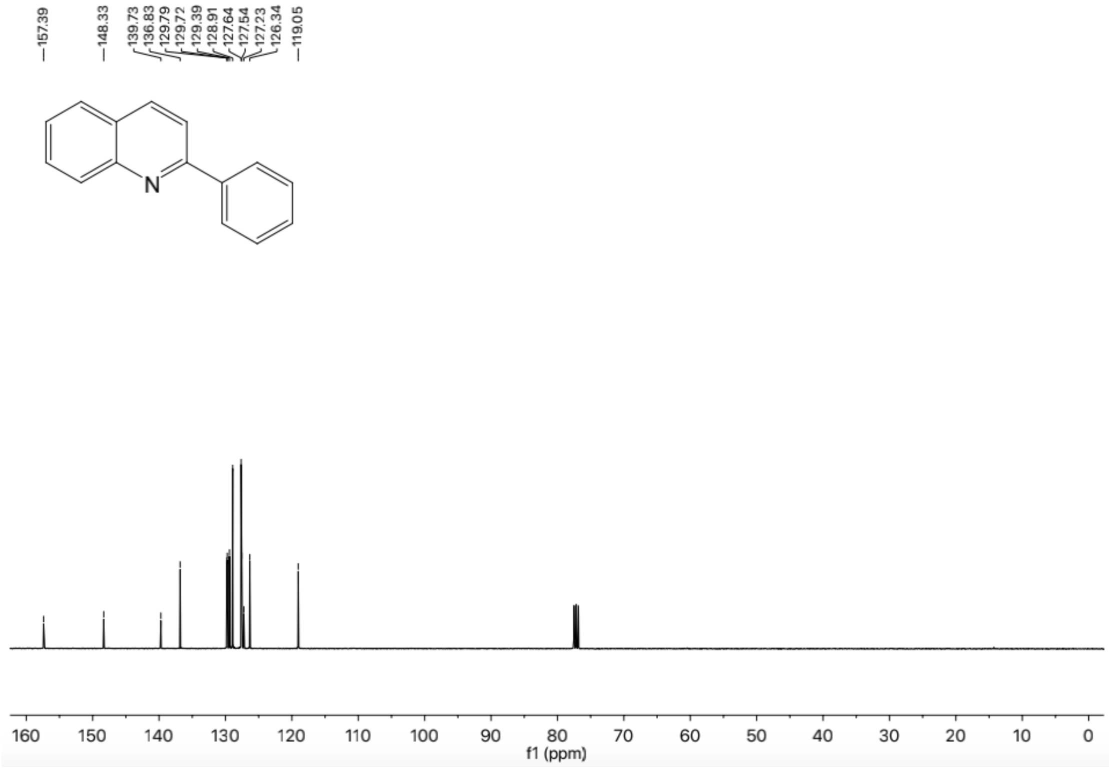

^{1}H NMR spectrum of 2-(4-methoxyphenyl)-3-methylquinoline (3ab)

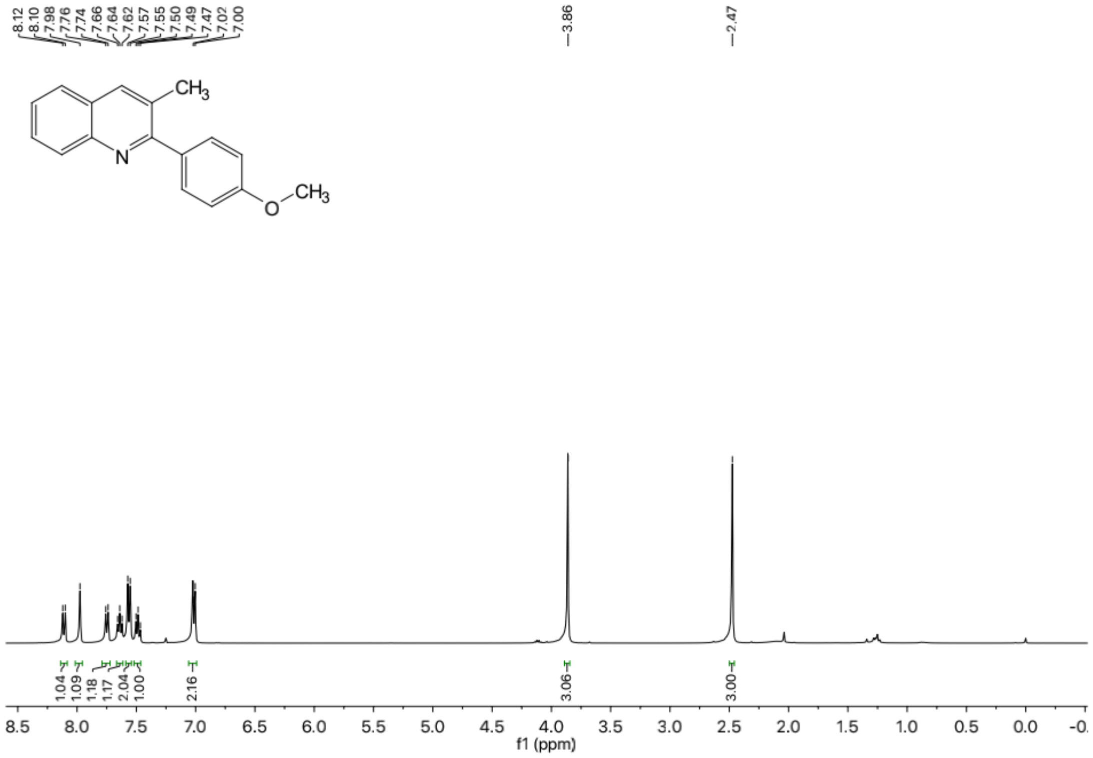

^{13}C NMR spectrum of 2-(4-methoxyphenyl)-3-methyl quinoline (3ab)

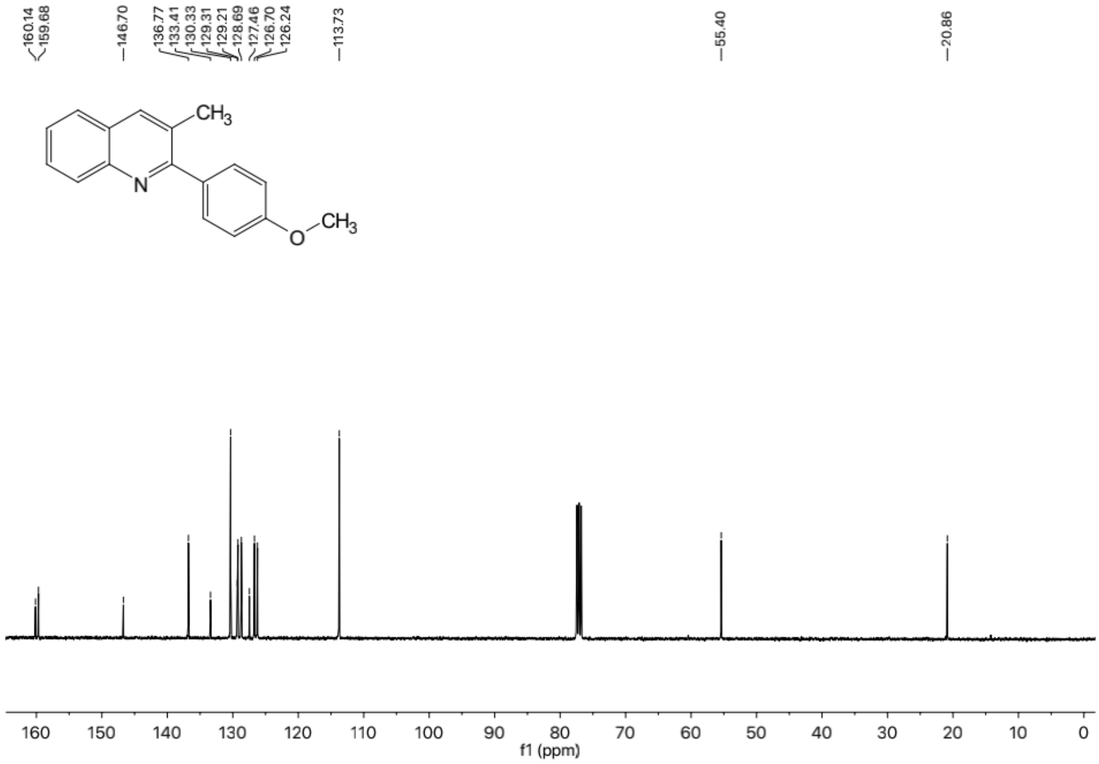

^{1}H NMR spectrum of 2-(4-methoxyphenyl)-3,8-dimethylquinoline（3bb）

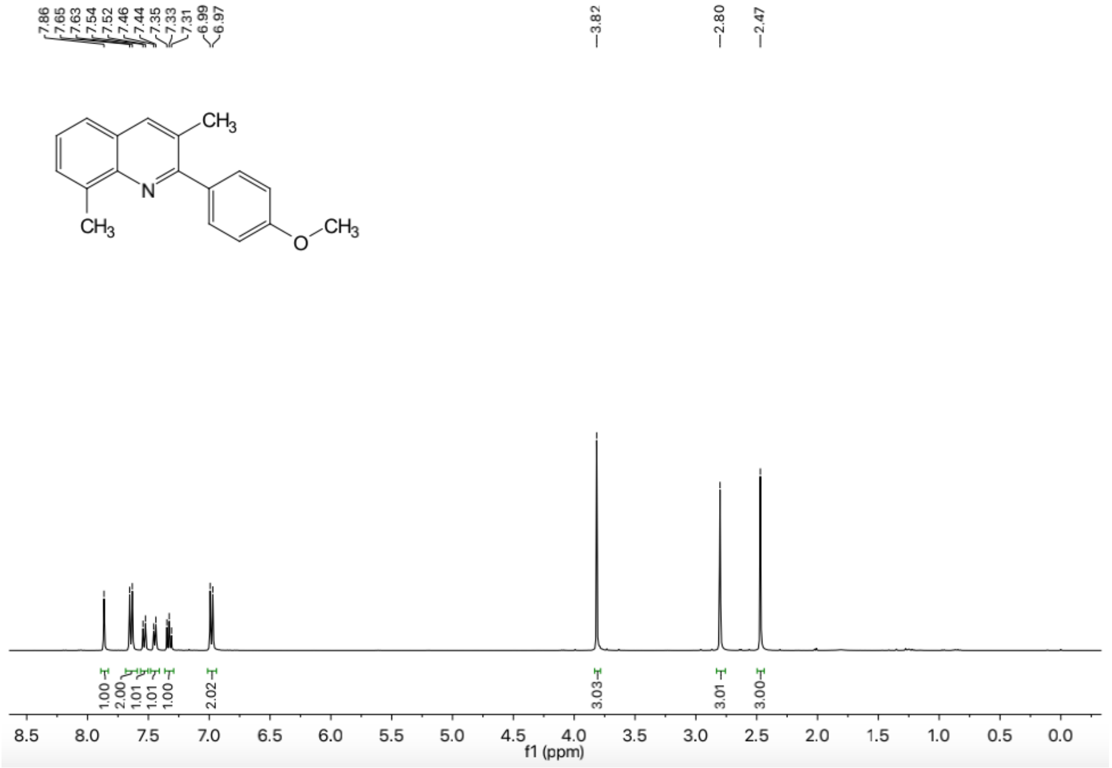

^{13}C NMR spectrum of 2-(4-methoxyphenyl)-3,8-dimethylquinoline（3bb）

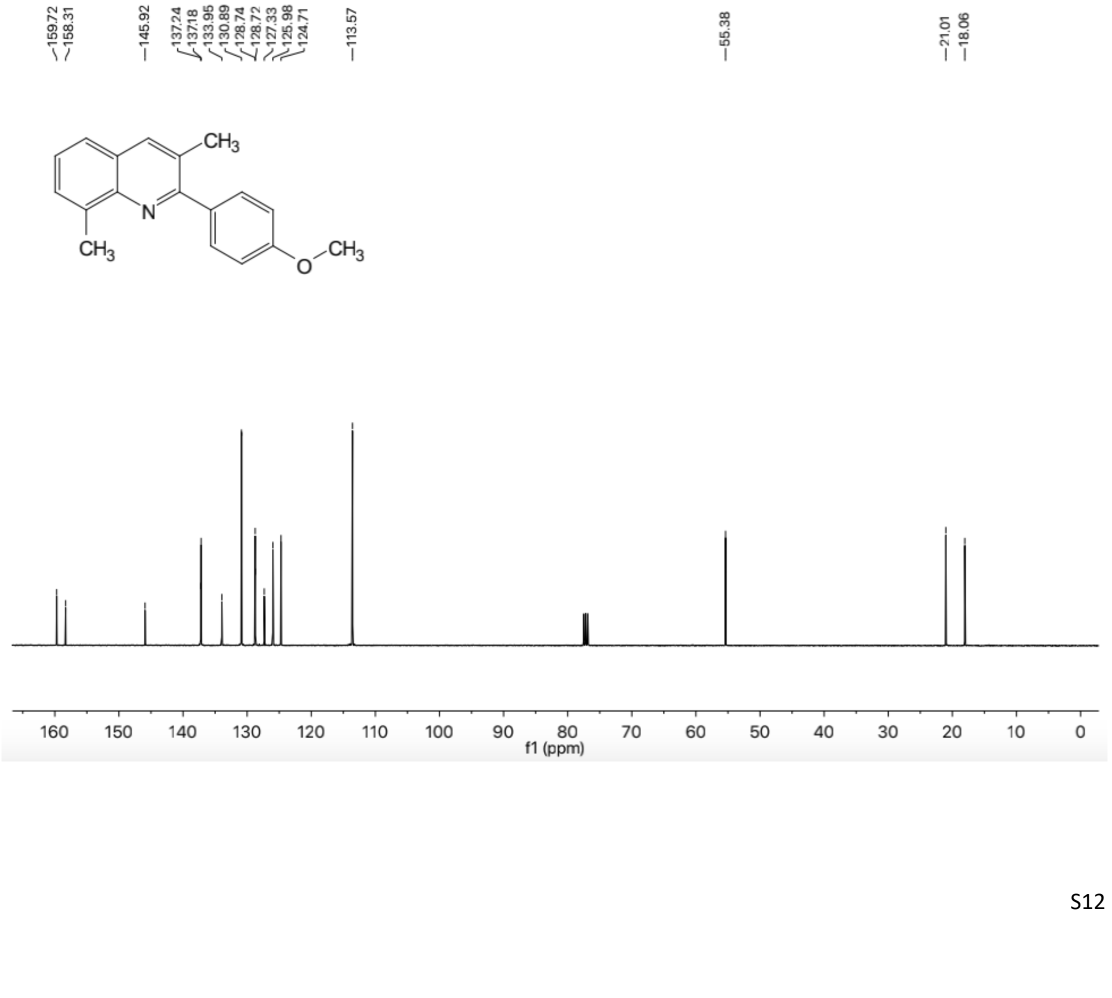

^{1}H NMR spectrum of 6-chloro-2-(4-methoxyphenyl)-3-methylquinoline（3cb）

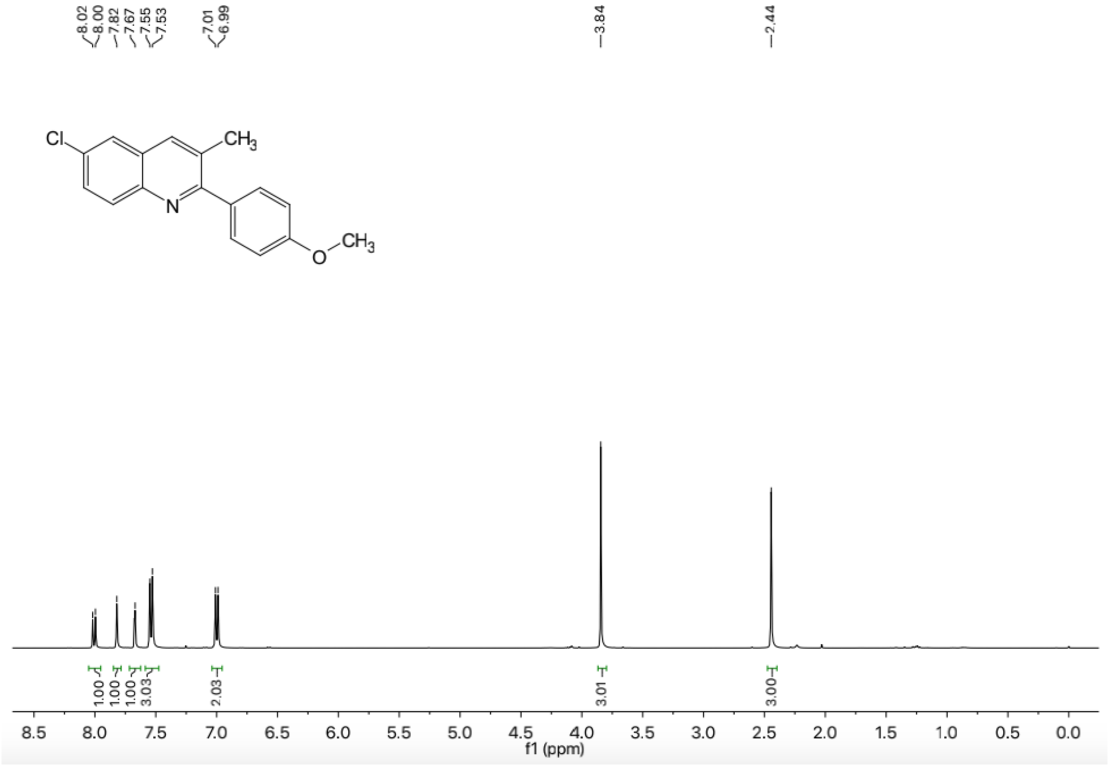

^{13}C NMR spectrum of 6-chloro-2-(4-methoxyphenyl)-3-methylquinoline（3cb）

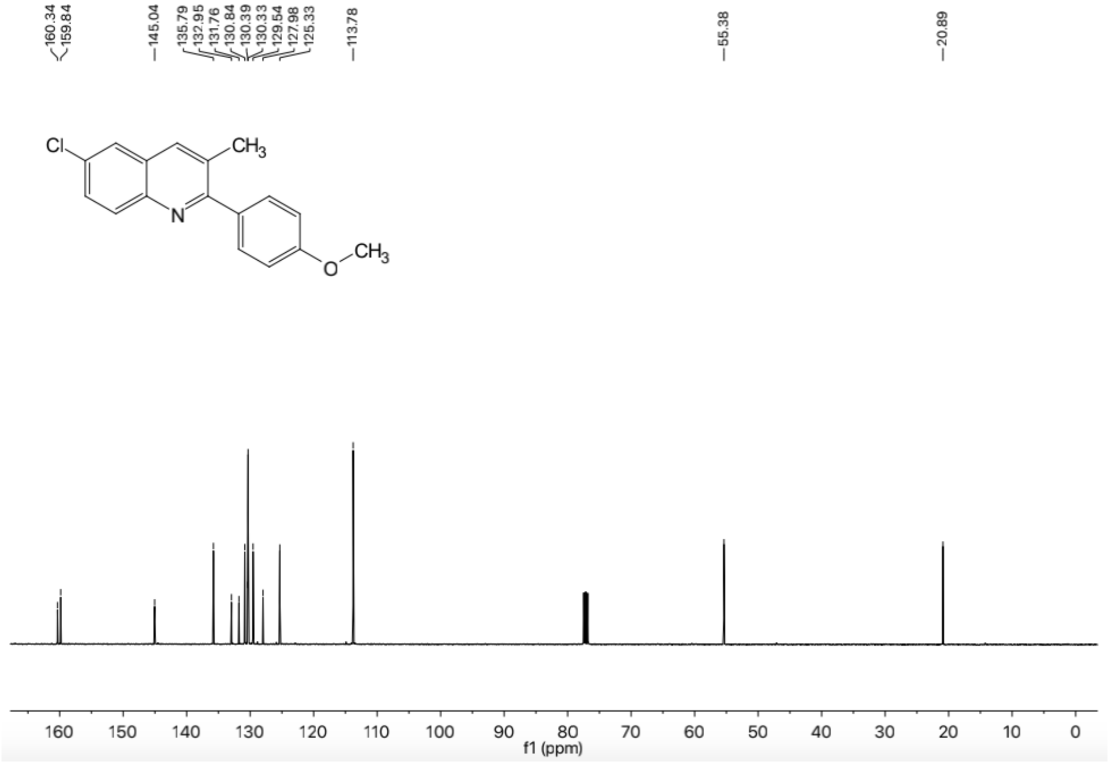

^{1}H NMR spectrum of 6,8-dibromo-2-(4-methoxyphenyl)-3-methylquinoline（3db）

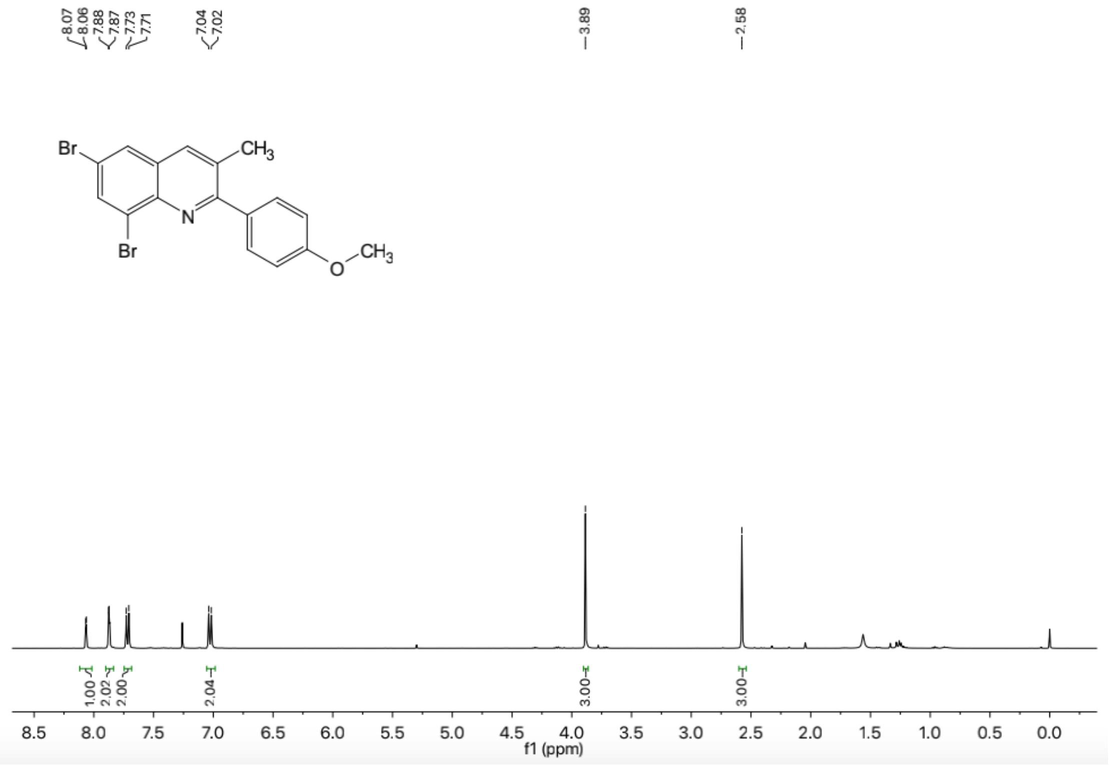

^{13}C NMR spectrum of 6,8-dibromo-2-(4-methoxyphenyl)-3-methylquinoline（3db）

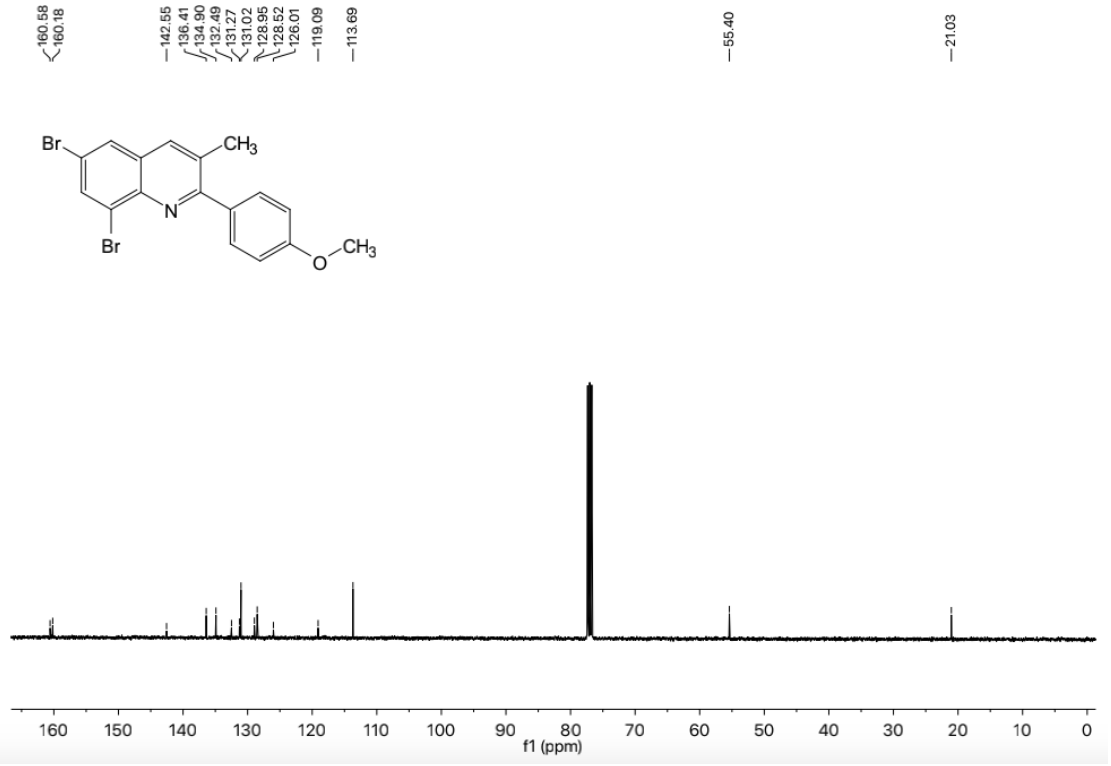

^{1}H NMR spectrum of 2-(4-chlorophenyl)-3-ethylquinoline（3ac）

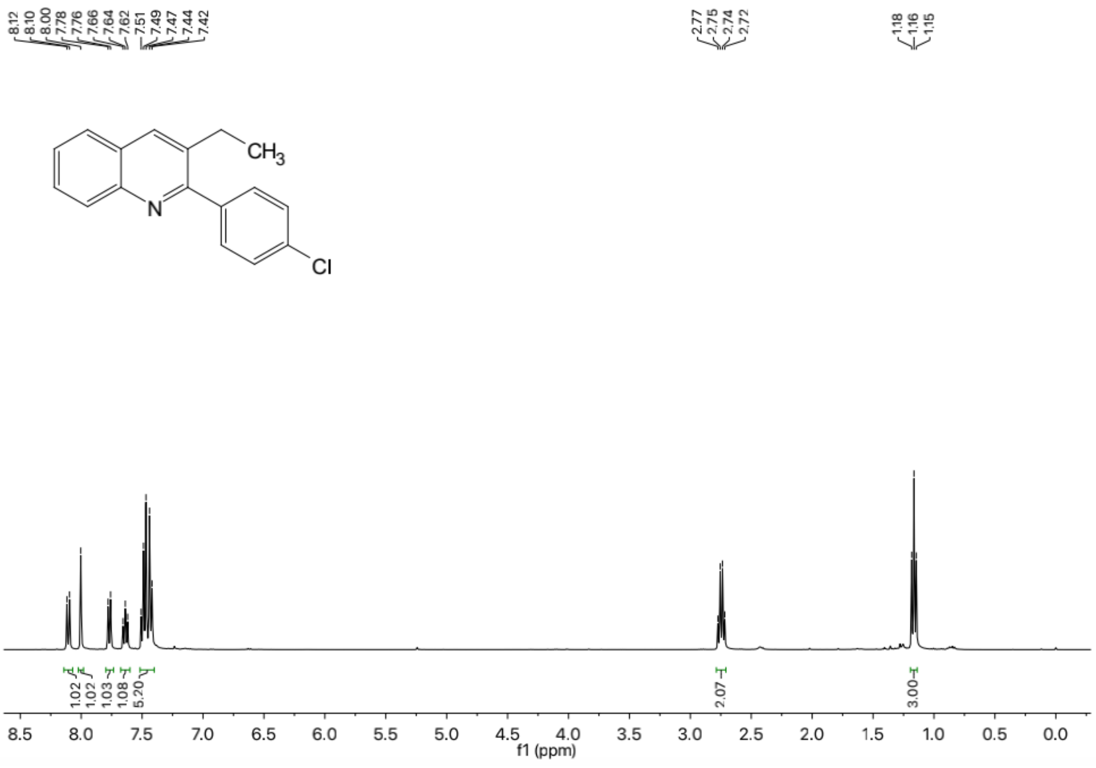

^{13}C NMR spectrum of 2-(4-chlorophenyl)-3-ethylquinoline（3ac）

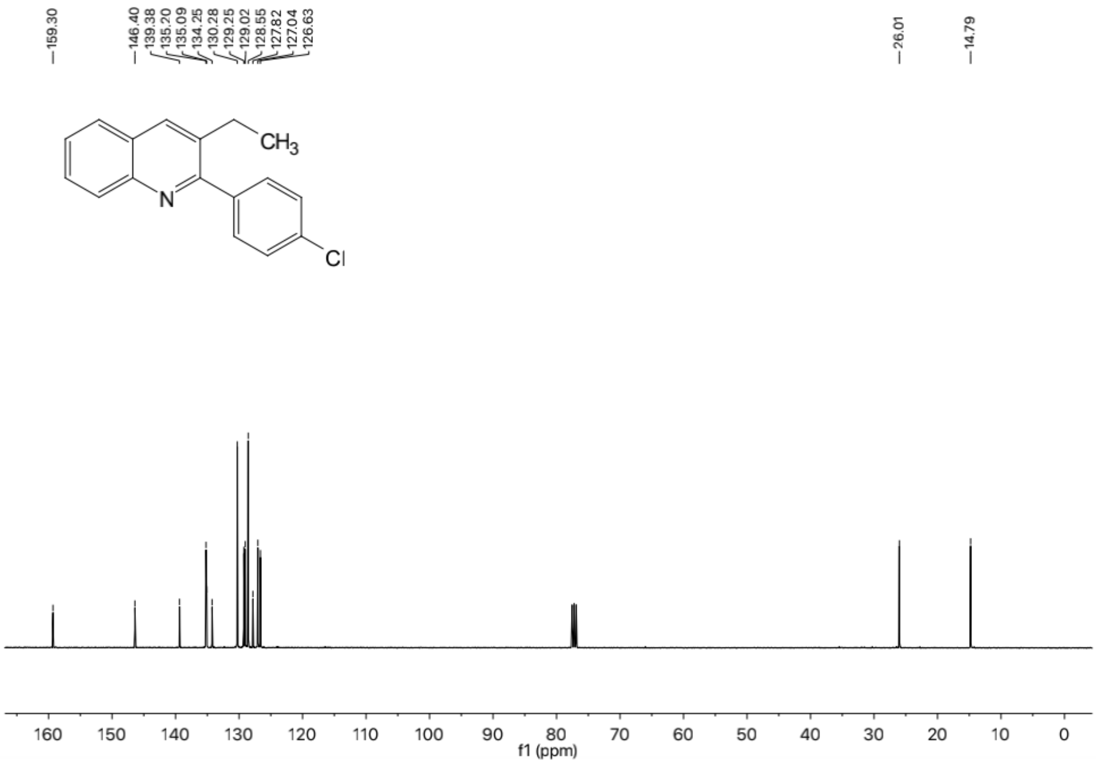

^{1}H **NMR spectrum of 2-(4-chlorophenyl)-3-ethyl-8-methylquinoline（3bc）**

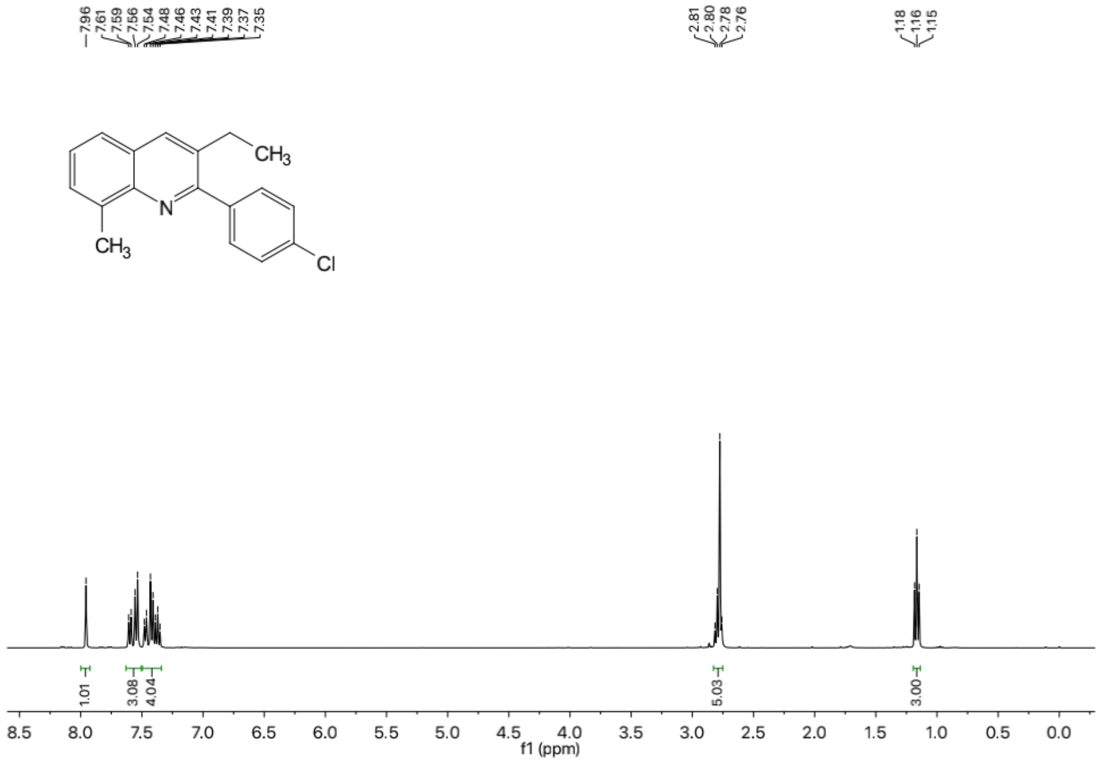

^{13}C **NMR spectrum of 2-(4-chlorophenyl)-3-ethyl-8-methylquinoline（3bc）**

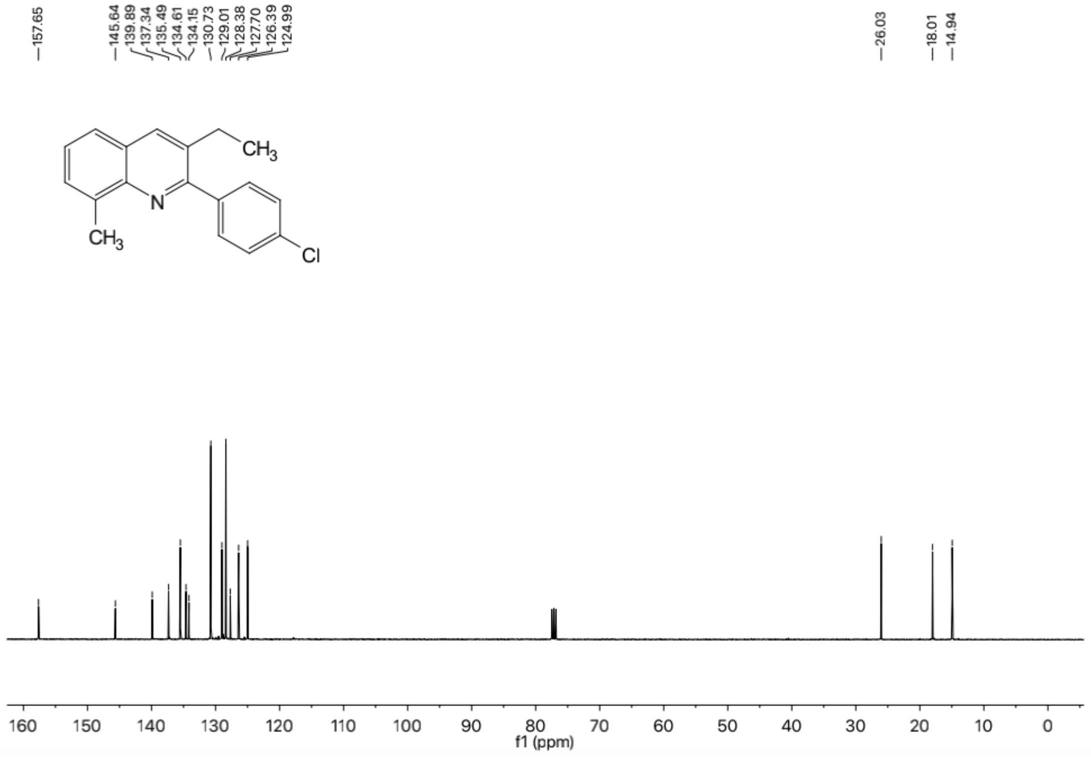

^{1}H NMR spectrum of 6-chloro-2-(4-chlorophenyl)-3-ethylquinoline（3cc）

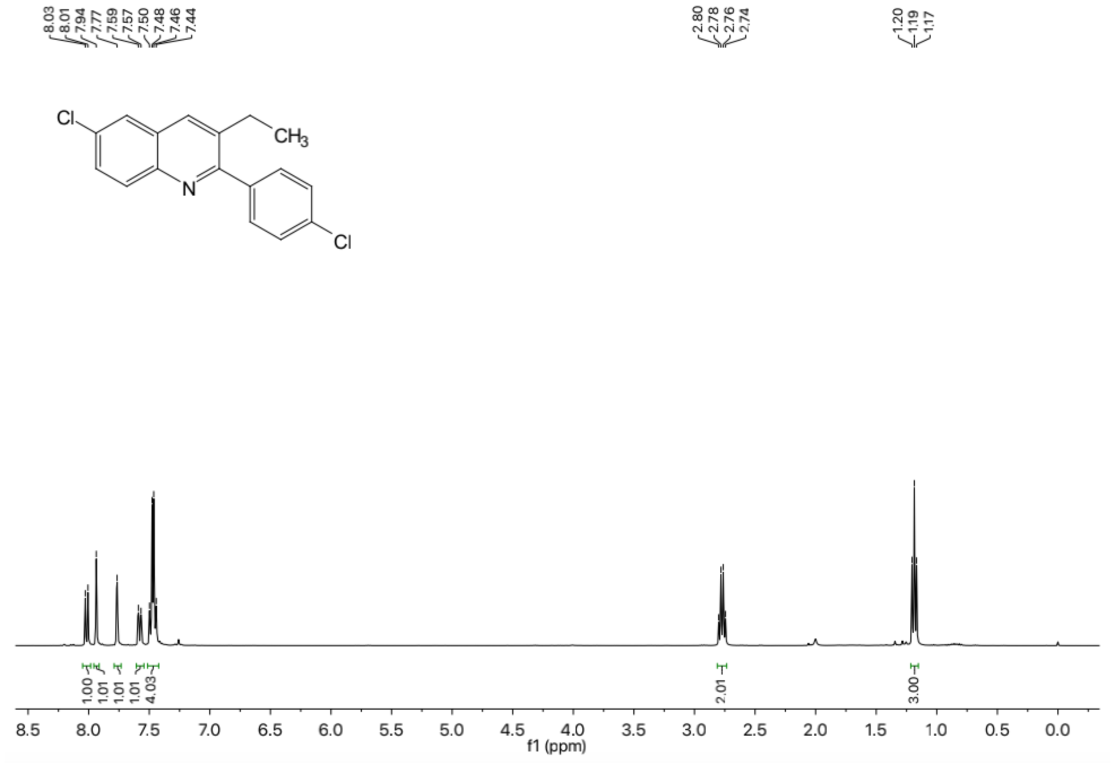

^{13}C NMR spectrum of 6-chloro-2-(4-chlorophenyl)-3-ethylquinoline （3cc）

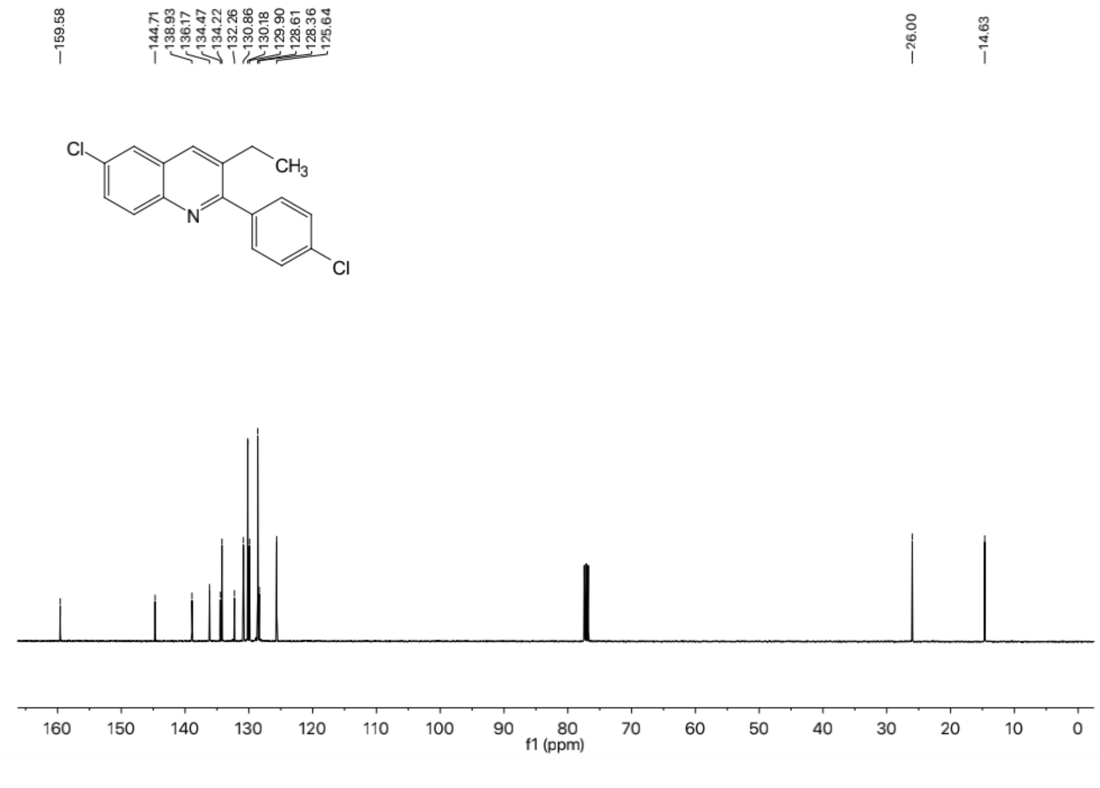

^{1}H NMR spectrum of 2-(4-chlorophenyl)-3-propylquinoline (3ad)

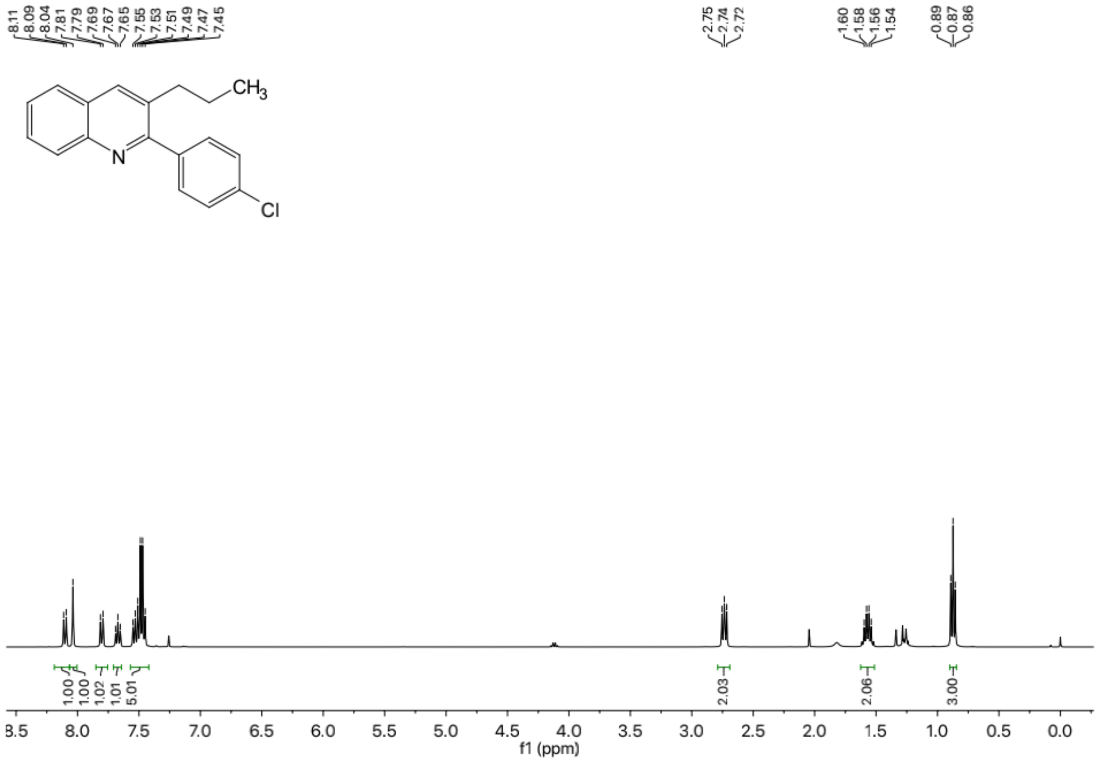

^{13}C NMR spectrum of 2-(4-chlorophenyl)-3-propylquinoline (3ad)

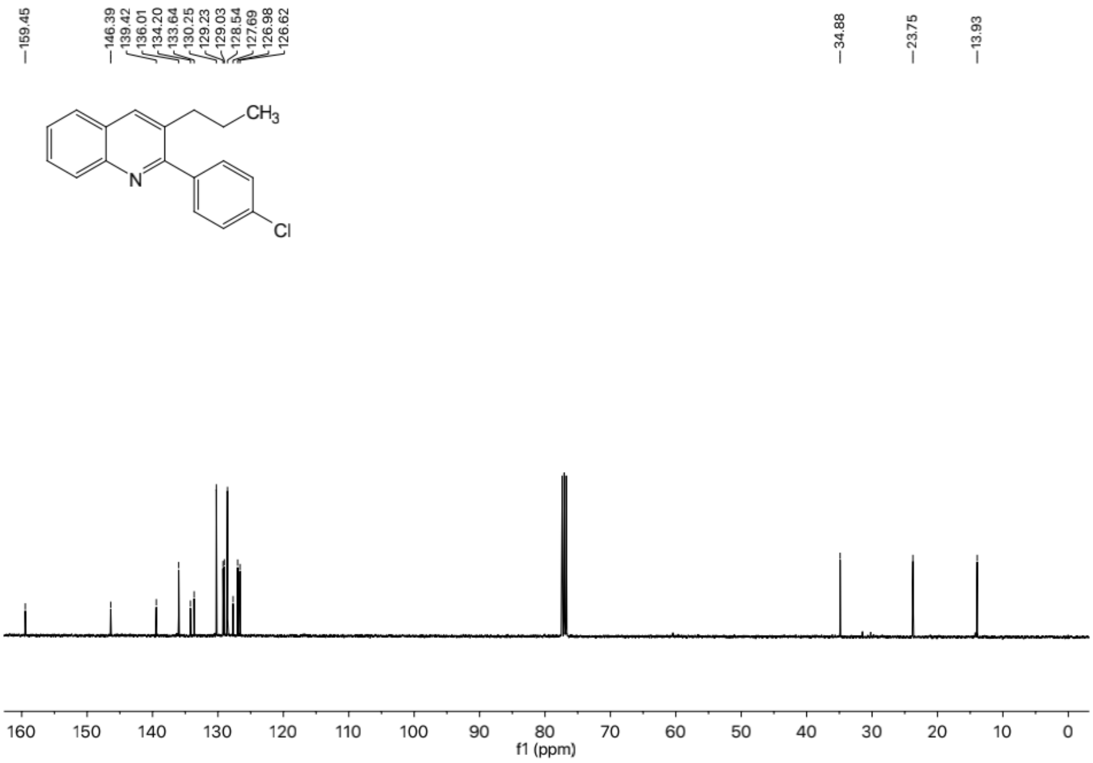

^{1}H NMR spectrum of 2-(4-chlorophenyl)-8-methyl-3-propylquinoline（3bd）

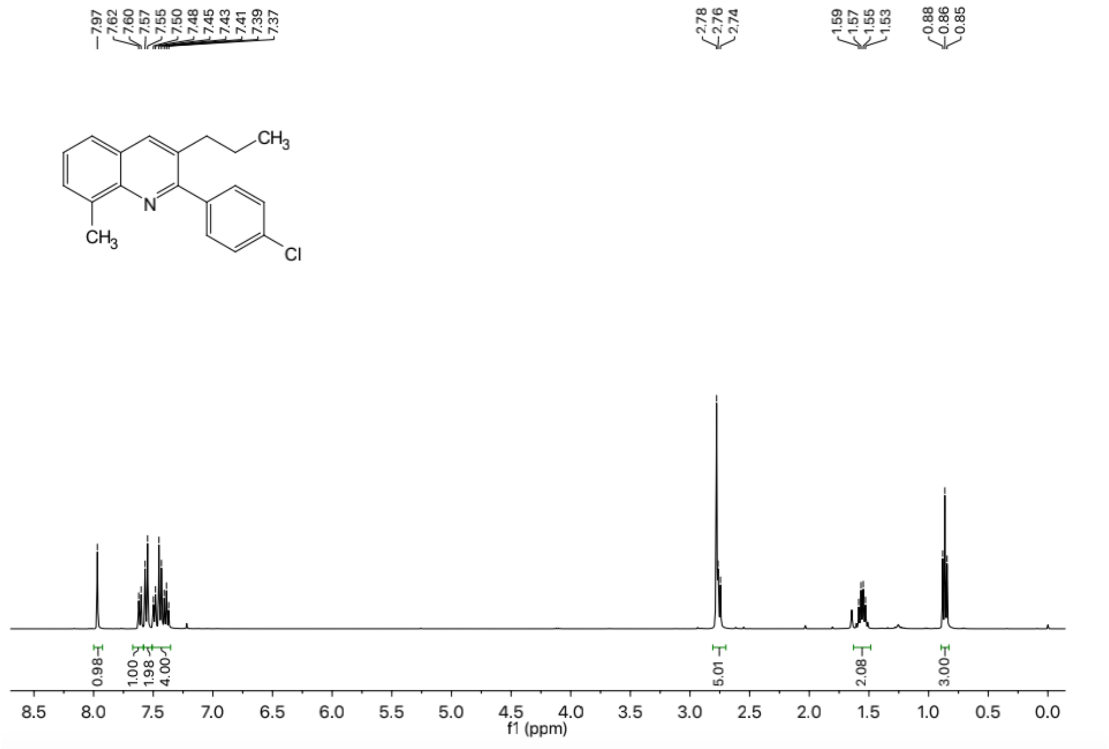

^{13}C NMR spectrum of 2-(4-chlorophenyl)-8-methyl-3-propylquinoline（3bd）

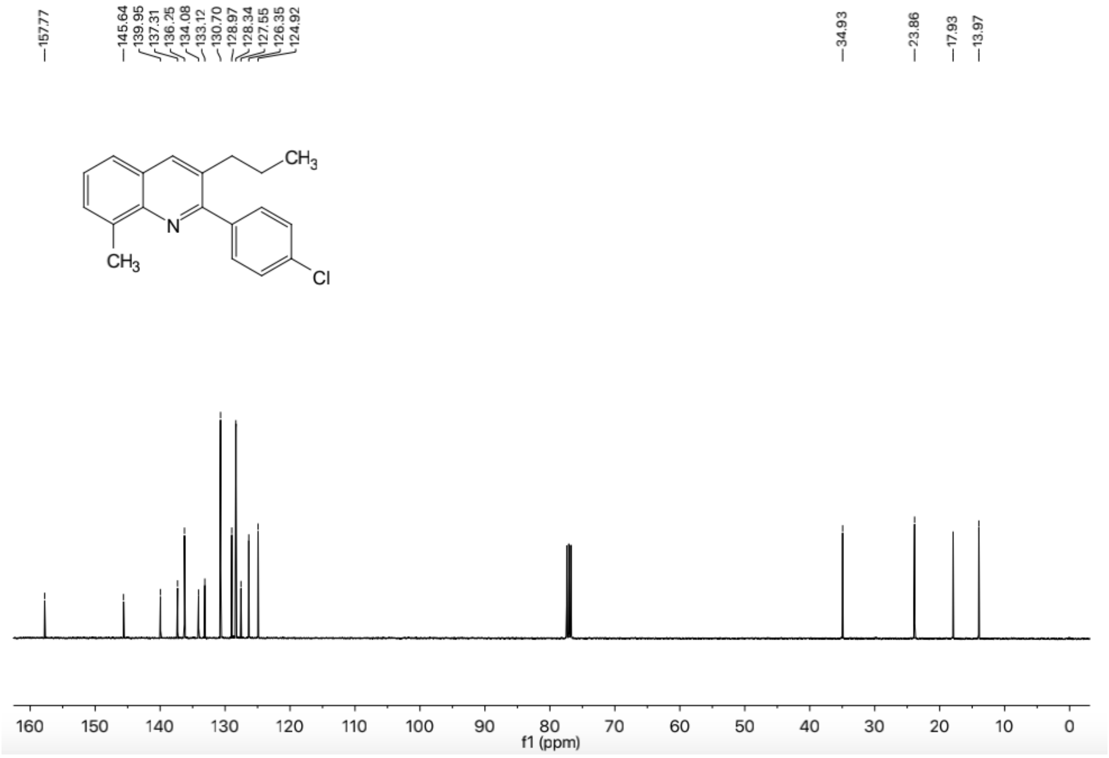

^{1}H NMR spectrum of 6-chloro-2-(4-chlorophenyl)-3-propylquinoline（3cd）

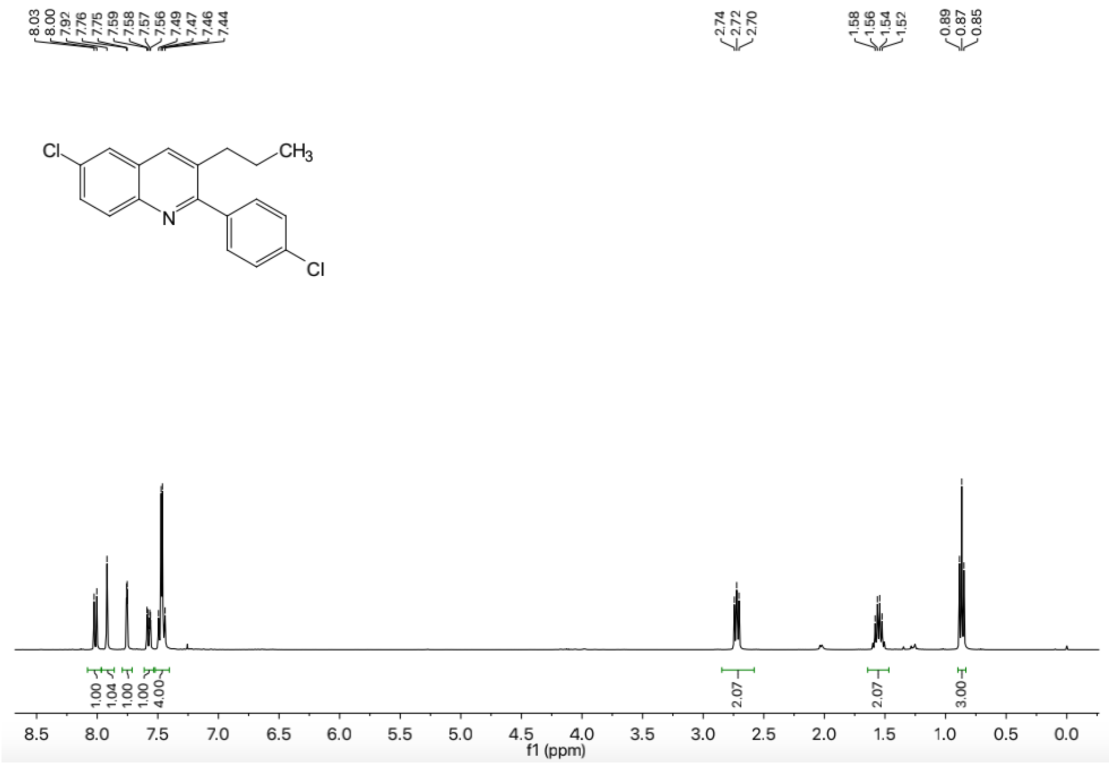

^{13}C NMR spectrum of 6-chloro-2-(4-chlorophenyl)-3-propylquinoline（3cd）

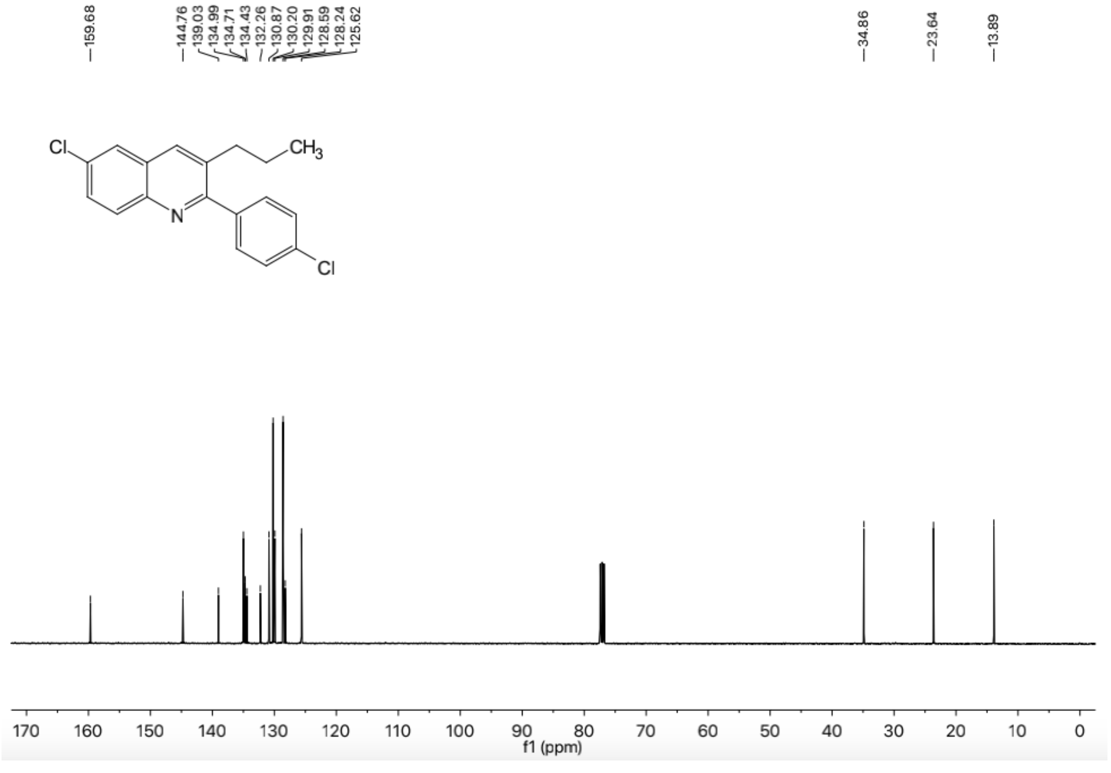

^{1}H NMR spectrum of 6,8-dibromo-2-(4-chlorophenyl)-3-propylquinoline（3dd）

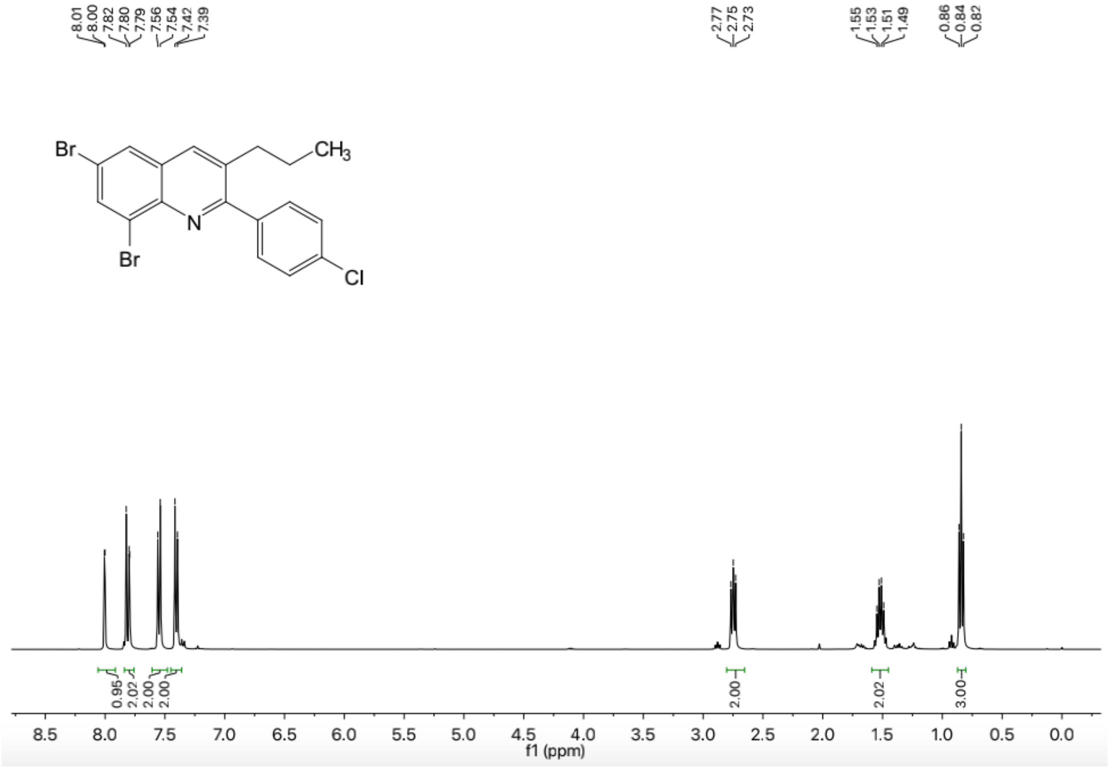

^{13}C NMR spectrum of 6,8-dibromo-2-(4-chlorophenyl)-3-propylquinoline（3dd）

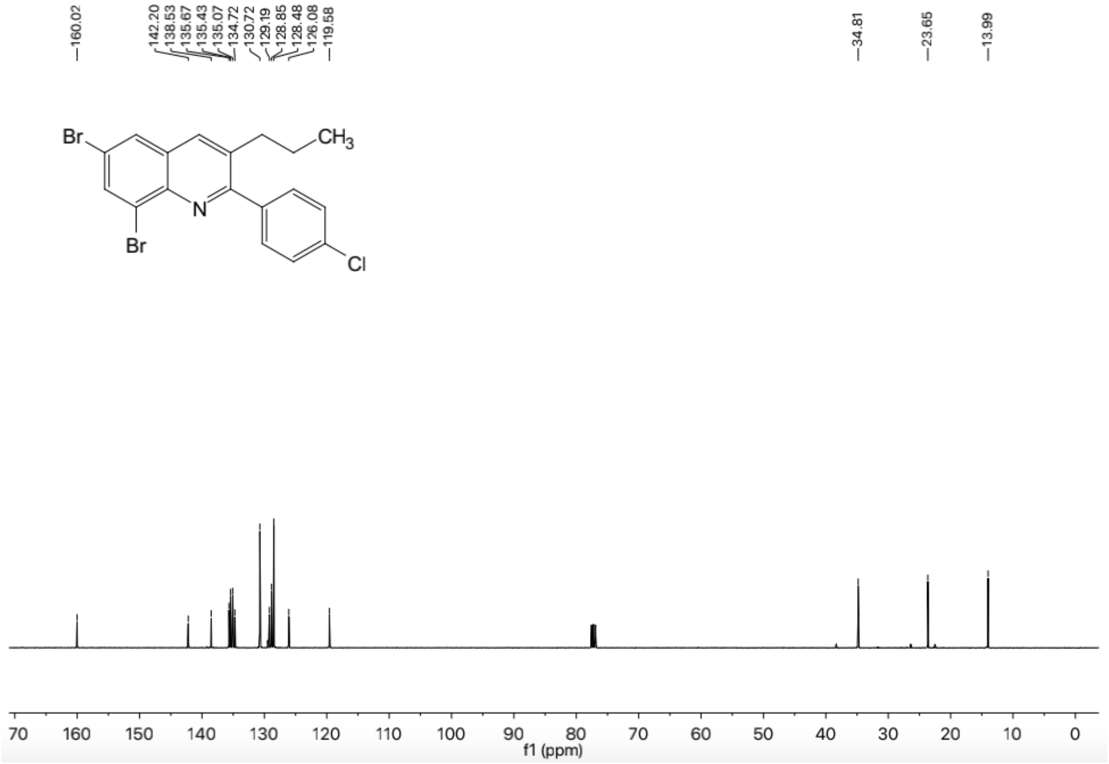

^{1}H NMR spectrum of 2-(4-bromophenyl)-3-propylquinoline（3ae）

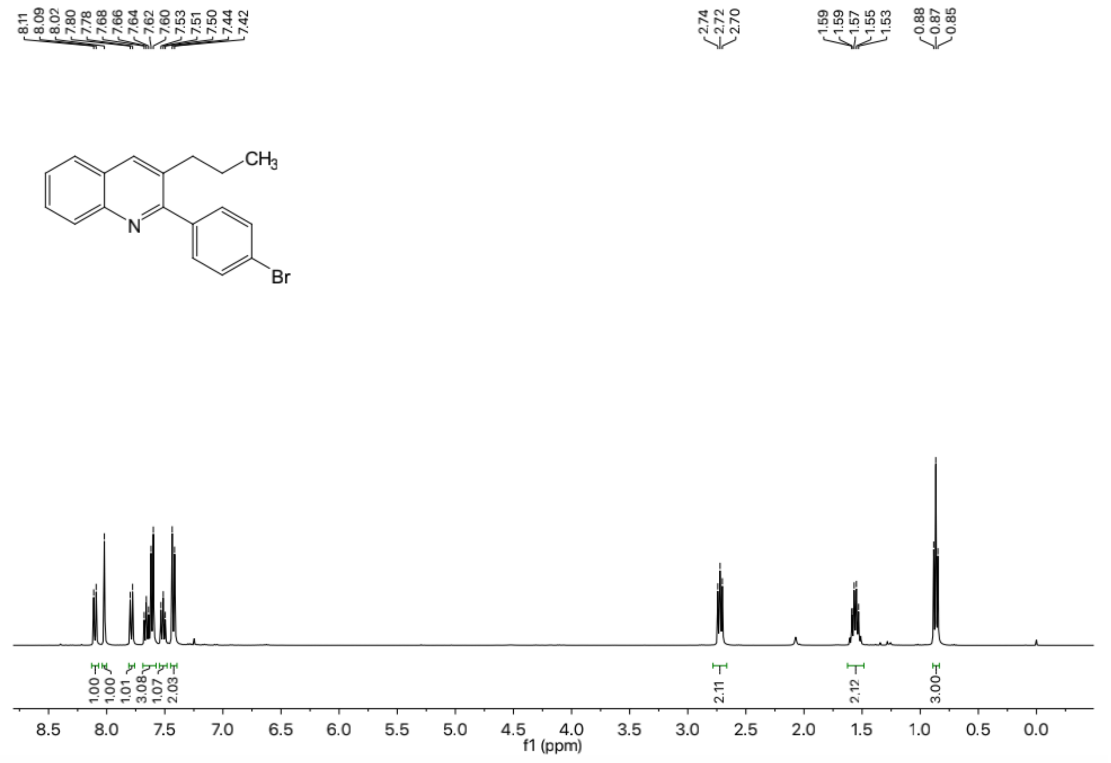

^{13}C NMR spectrum of 2-(4-bromophenyl)-3-propylquinoline（3ae）

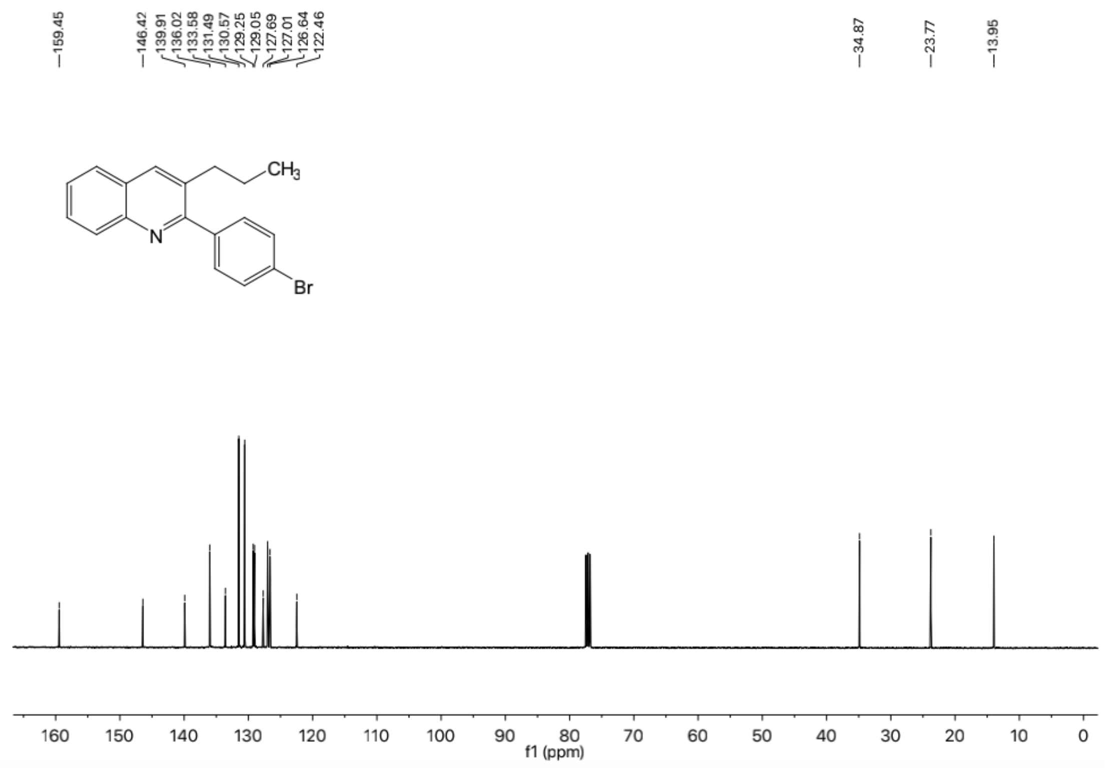

^{1}H NMR spectrum of 2-(4-bromophenyl)-8-methyl-3-propylquinoline（3be）

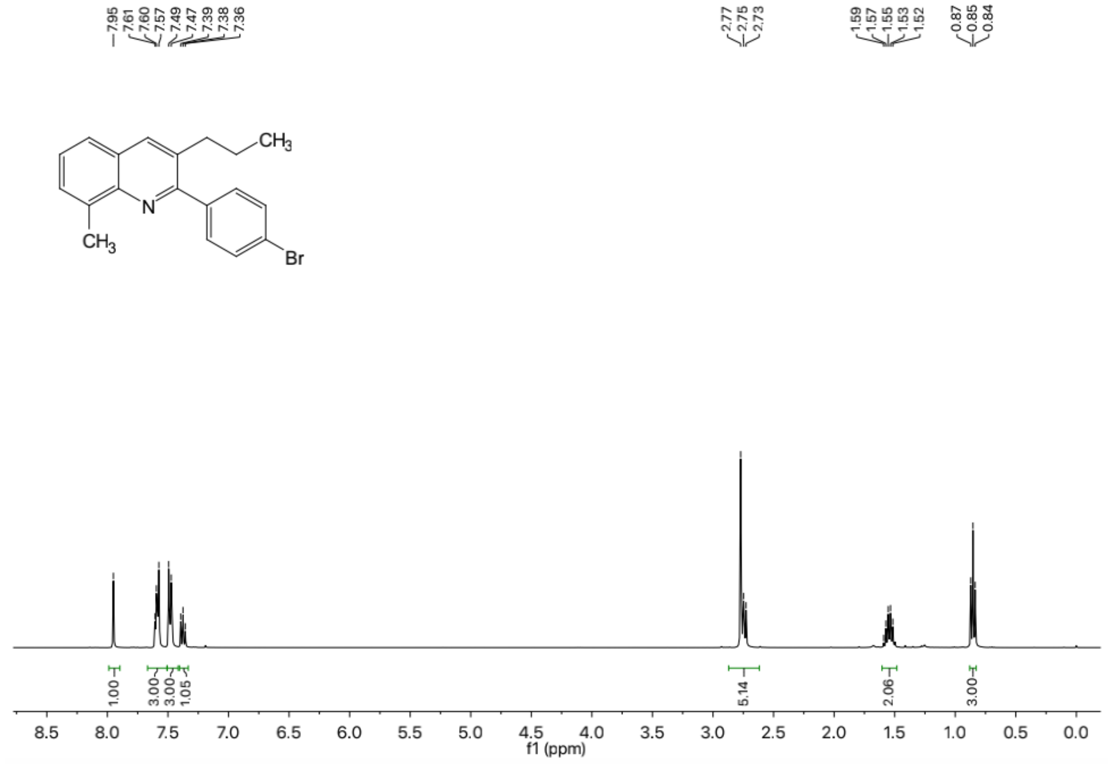

^{13}C NMR spectrum of 2-(4-bromophenyl)-8-methyl-3-propylquinoline（3be）

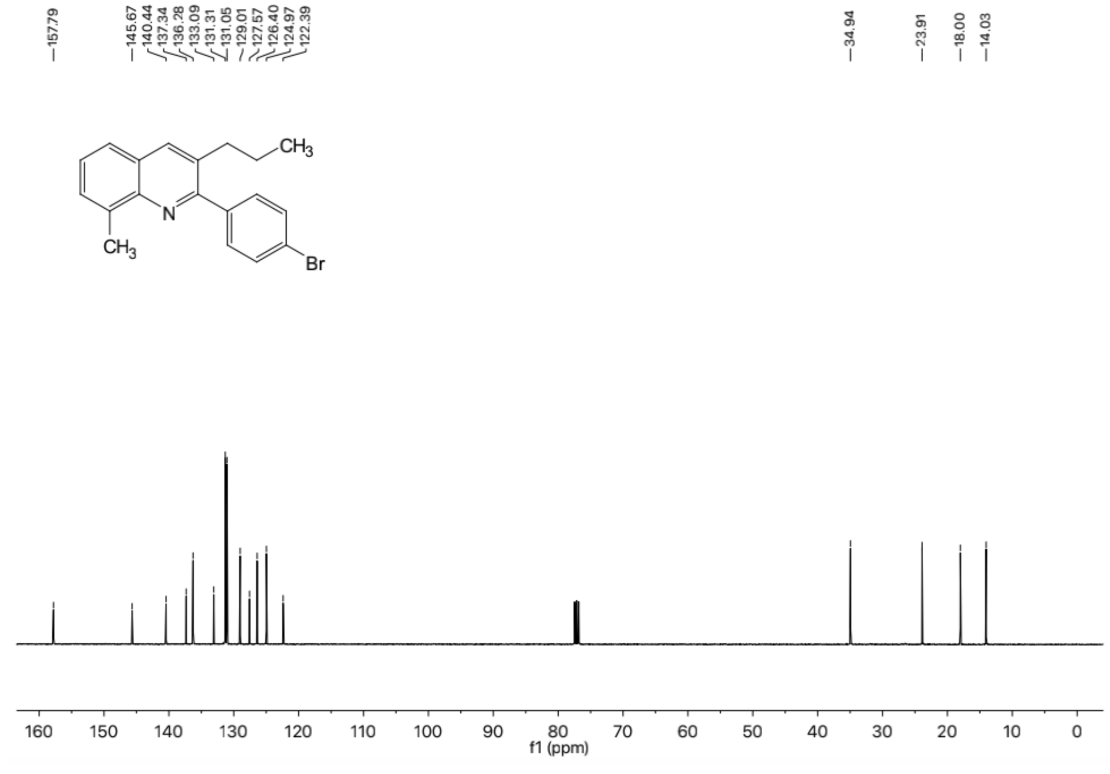

^{1}H NMR spectrum of 2-(4-bromophenyl)-6-chloro-3-propylquinoline（3ce）

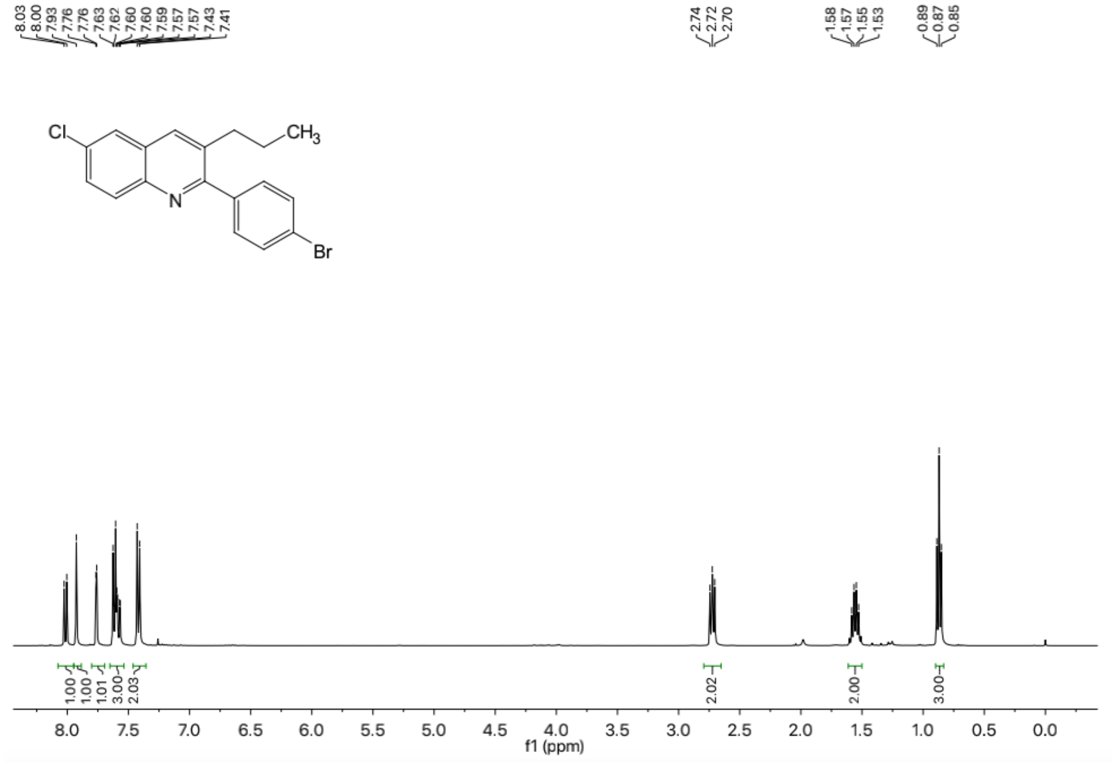

^{13}C NMR spectrum of 2-(4-bromophenyl)-6-chloro-3-propylquinoline（3ce）

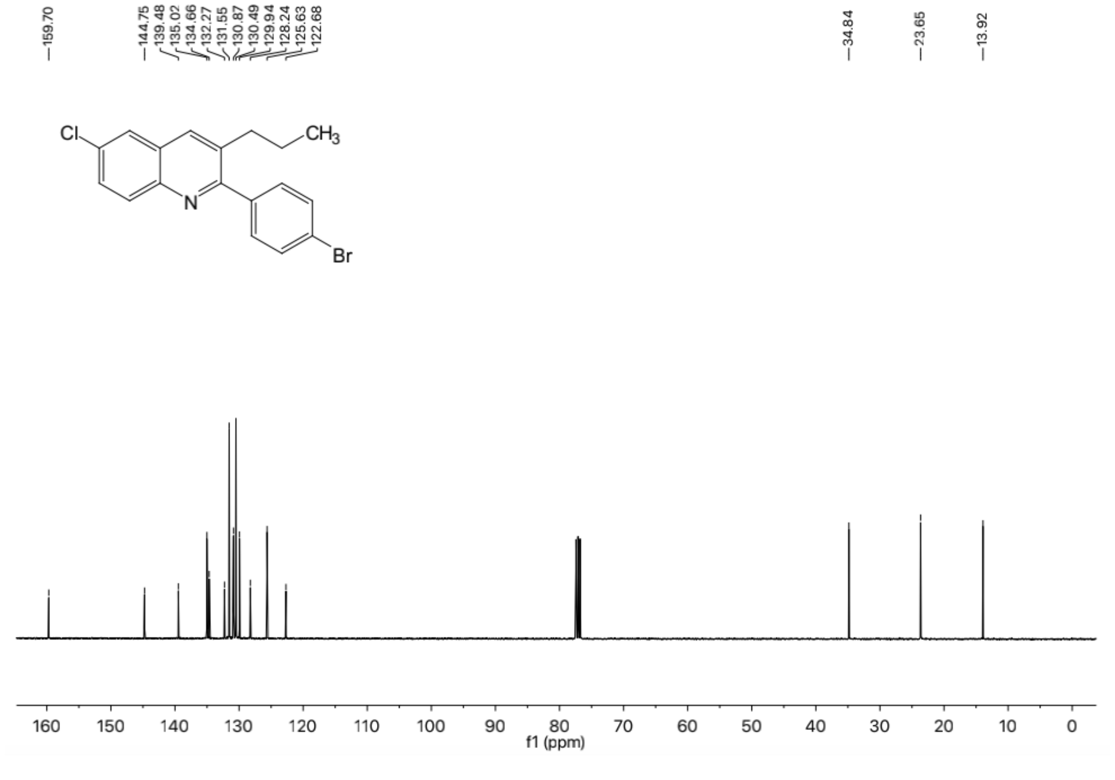

^{1}H NMR spectrum of 6,8-dibromo-2-(4-bromophenyl)-3-propylquinoline（3de）

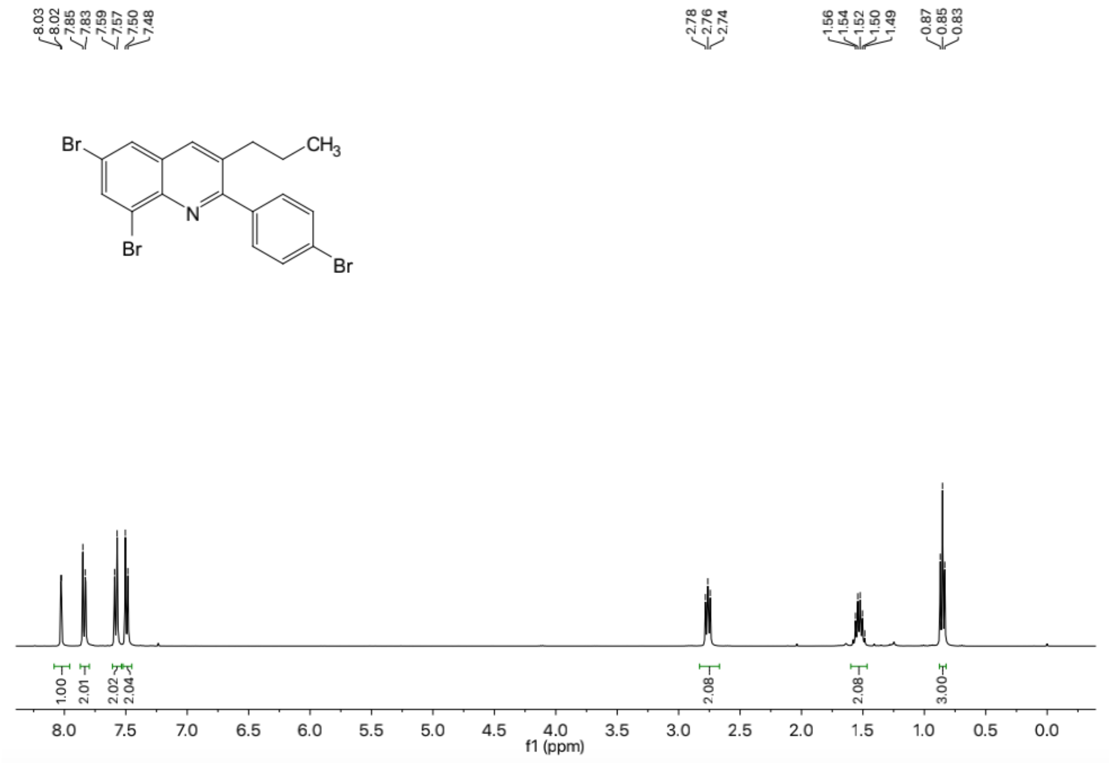

^{13}C NMR spectrum of 6,8-dibromo-2-(4-bromophenyl)-3-propylquinoline （3de）

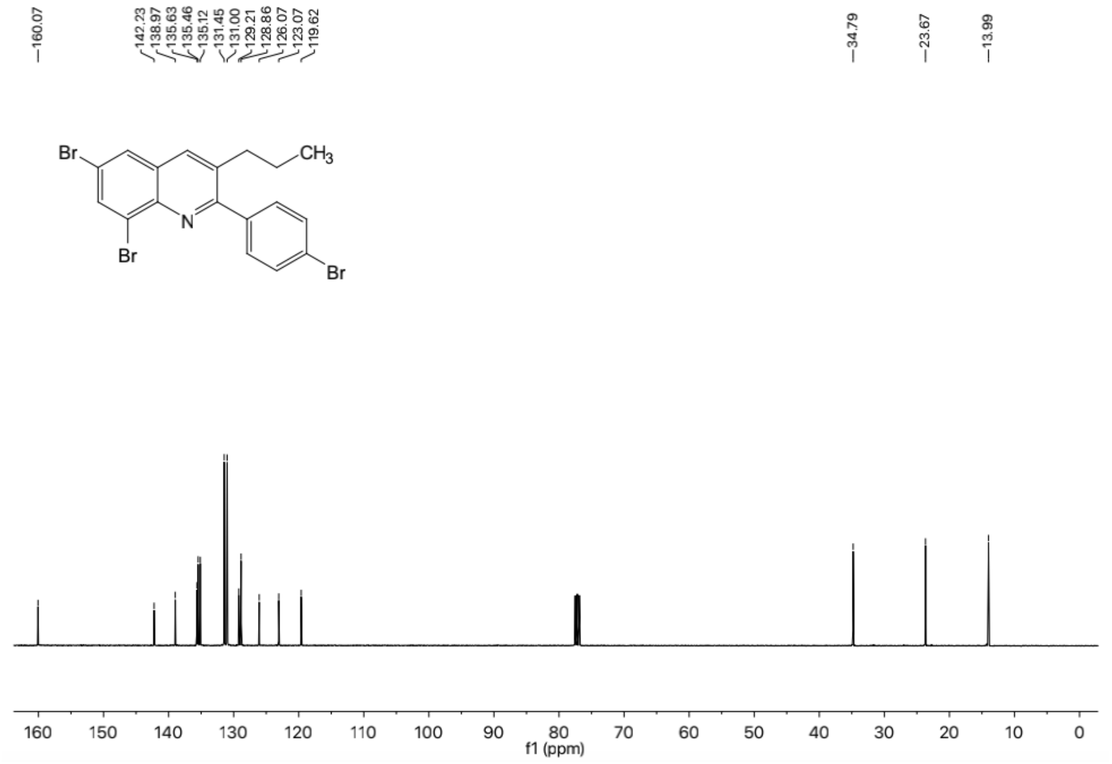

^{1}H NMR spectrum of 2-(4-fluorophenyl)-3-propylquinoline（3af）

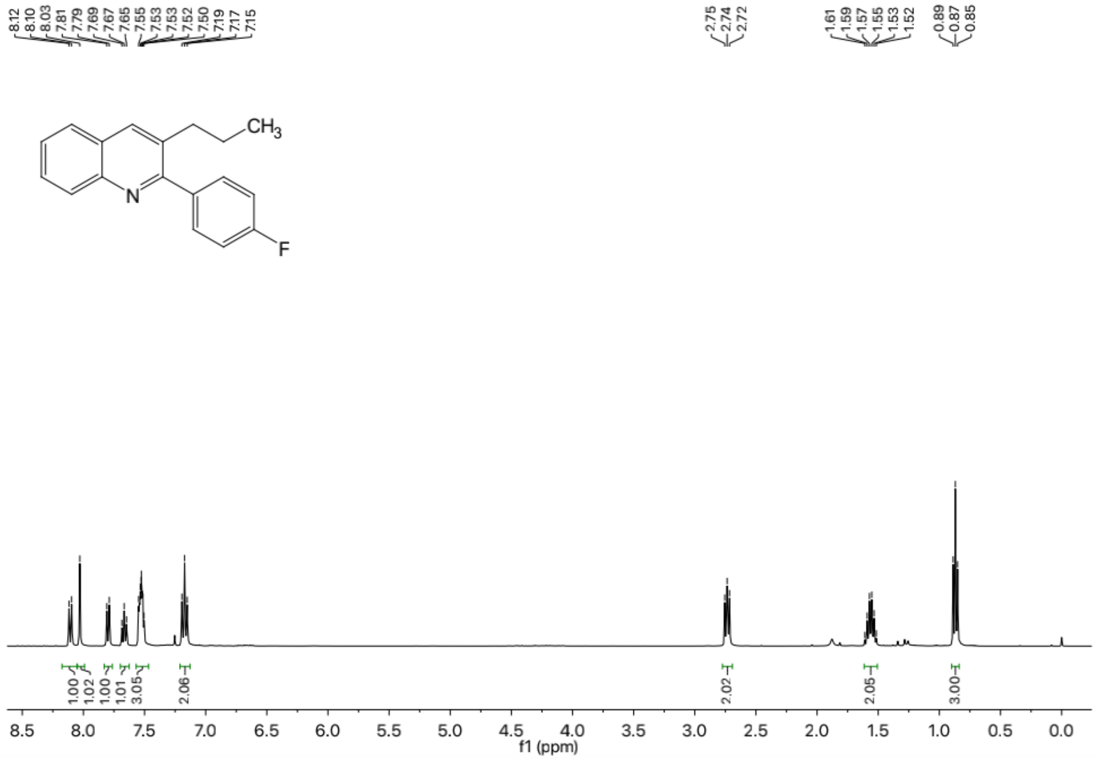

^{13}C NMR spectrum of 2-(4-fluorophenyl)-3-propylquinoline（3af）

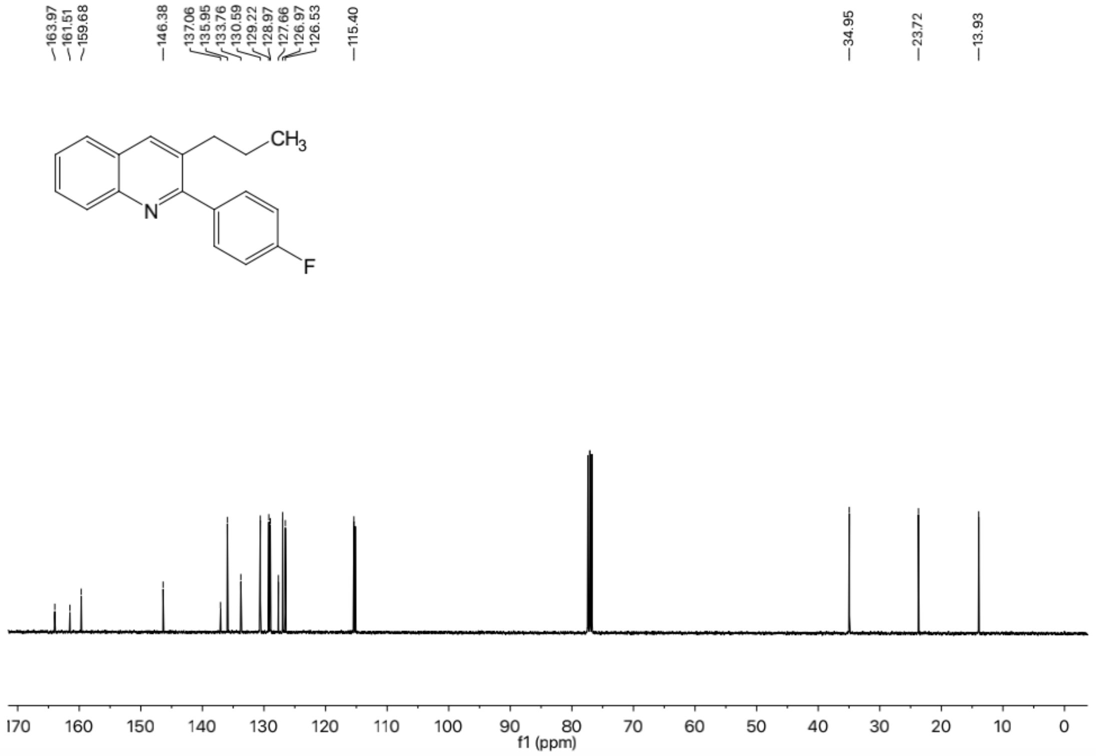

^{1}H NMR spectrum of 2-(4-fluorophenyl)-8-methyl-3-propylquinoline（3bf）

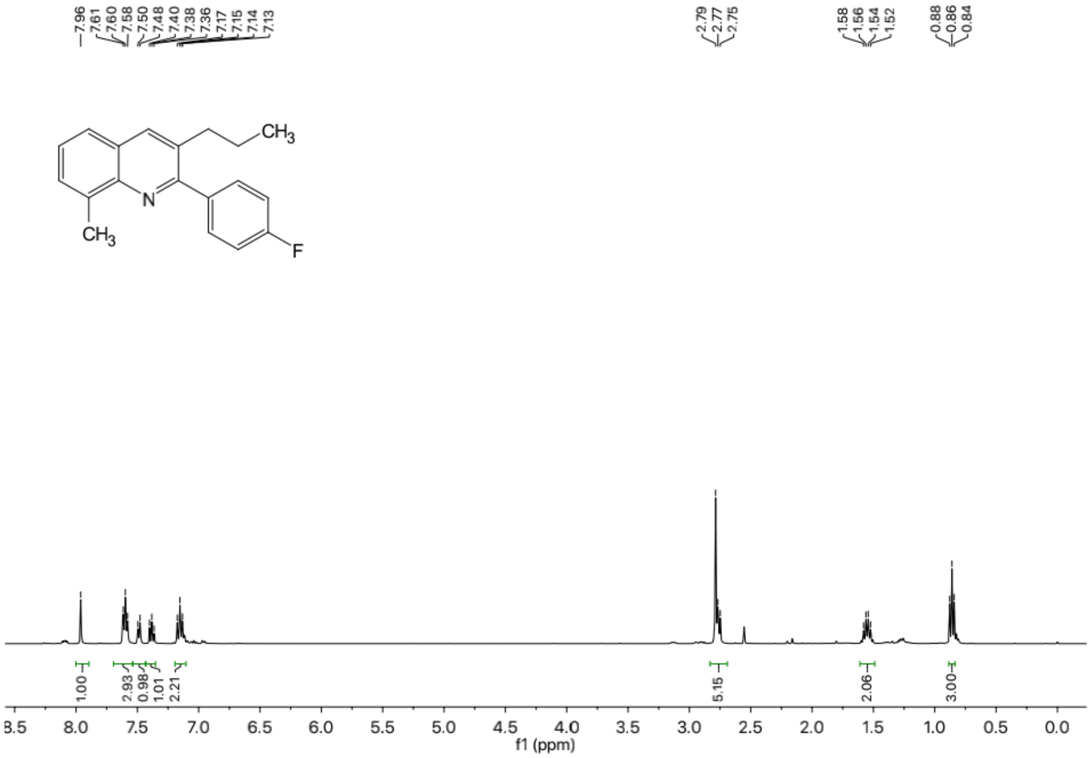

^{13}C NMR spectrum of 2-(4-fluorophenyl)-8-methyl-3-propylquinoline（3bf）

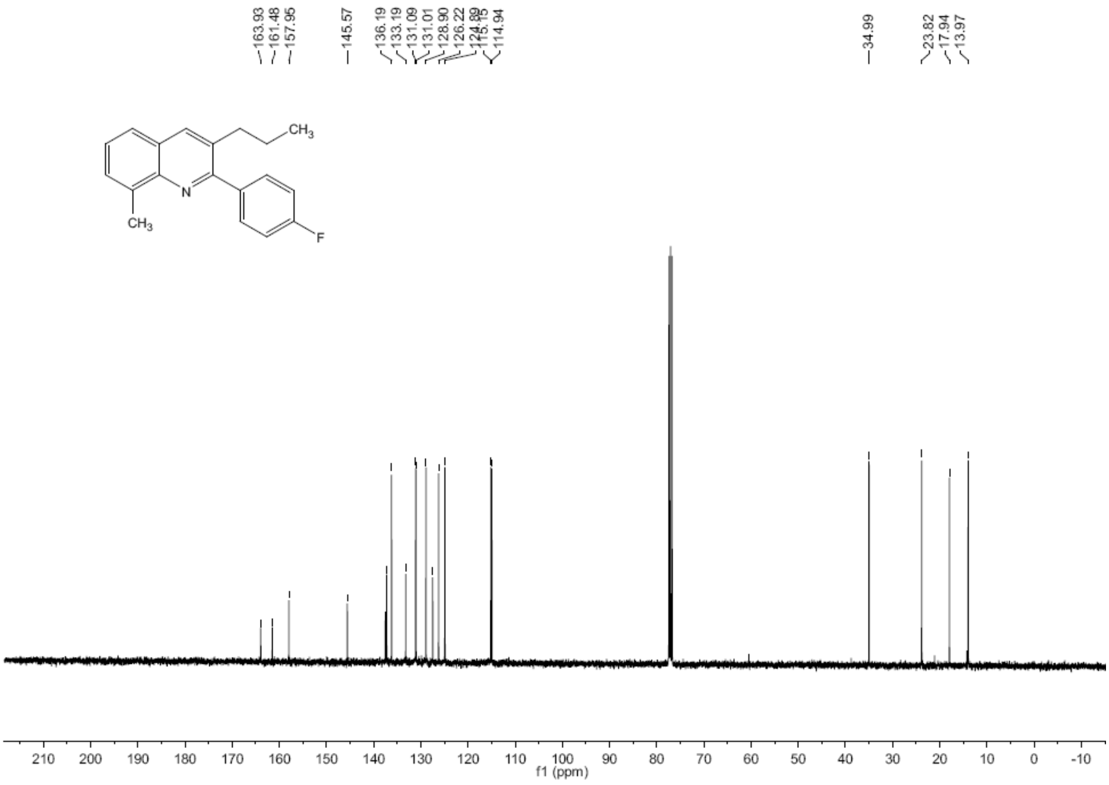

^{1}H NMR spectrum of 6-chloro-2-(4-fluorophenyl)-3-propylquinoline（3cf）

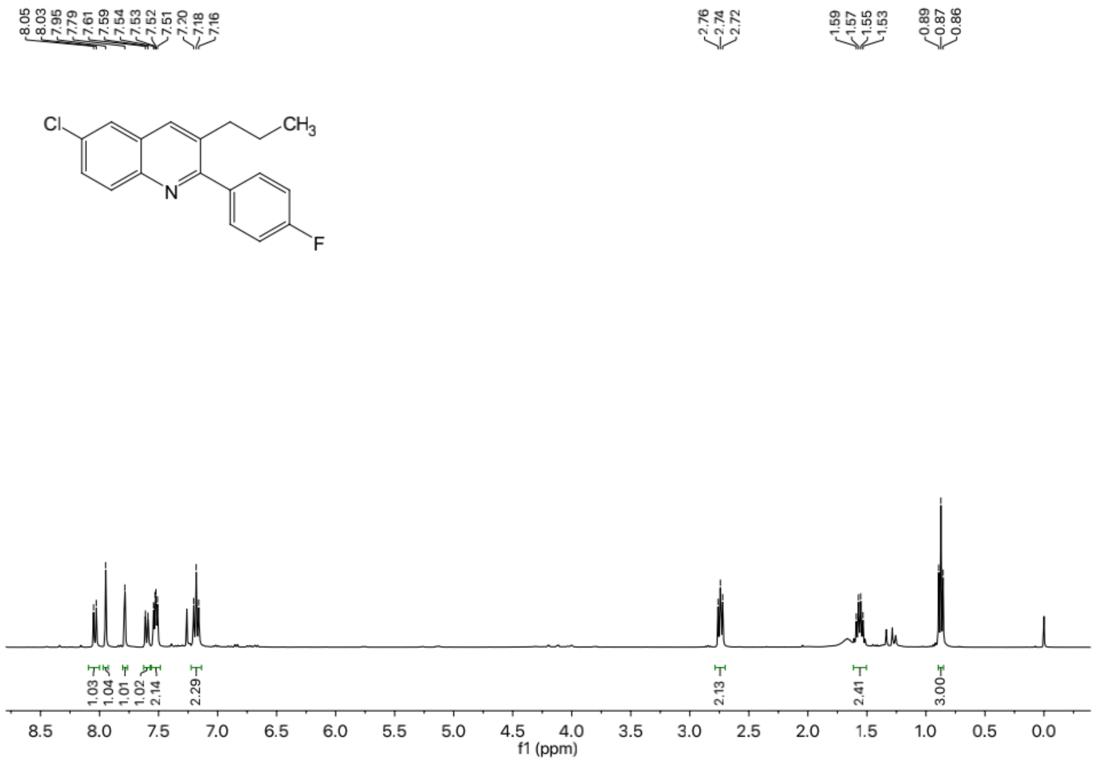

^{13}C NMR spectrum of 6-chloro-2-(4-fluorophenyl)-3-propylquinoline（3cf）

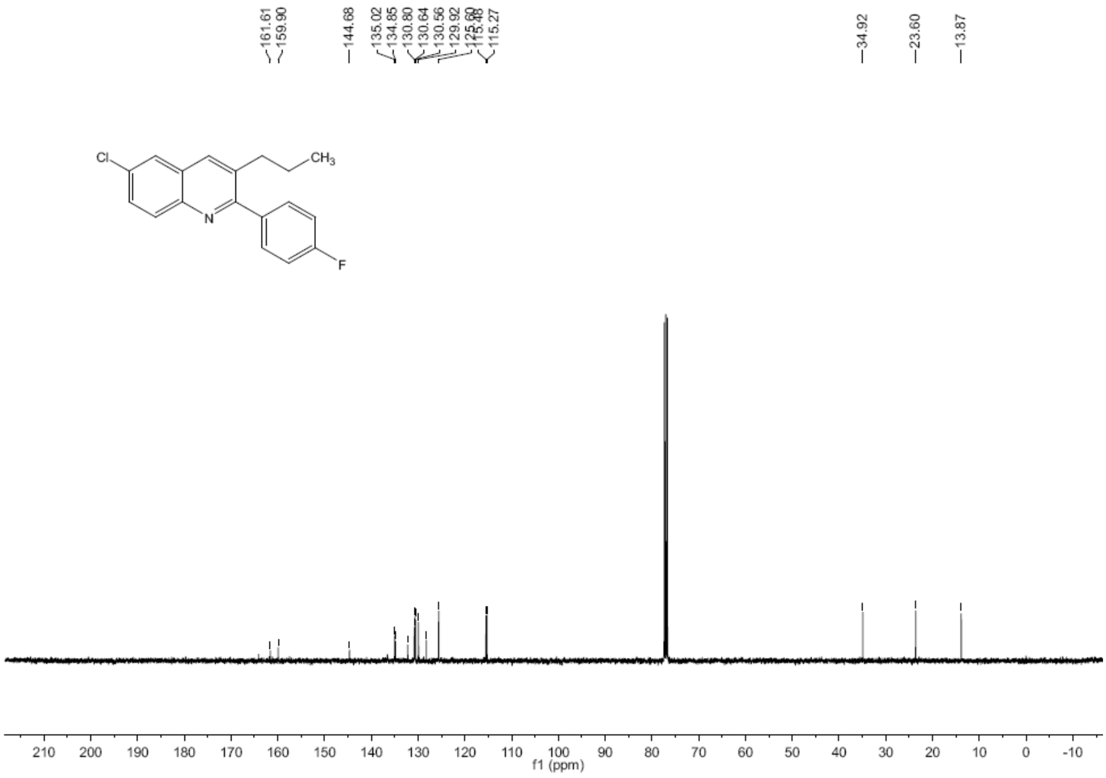

^{1}H NMR spectrum of 6,8-dibromo-2-(4-fluorophenyl)-3-propylquinoline（3df）

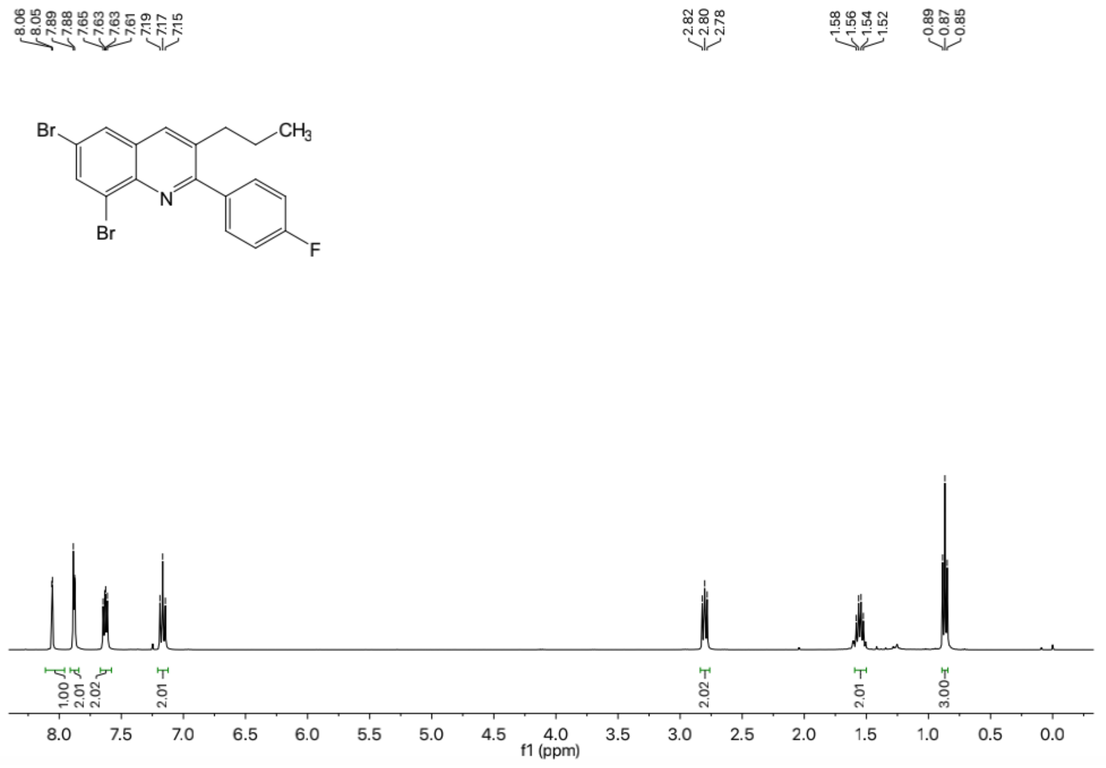

^{13}C NMR spectrum of 6,8-dibromo-2-(4-fluorophenyl)-3-propylquinoline（3df）

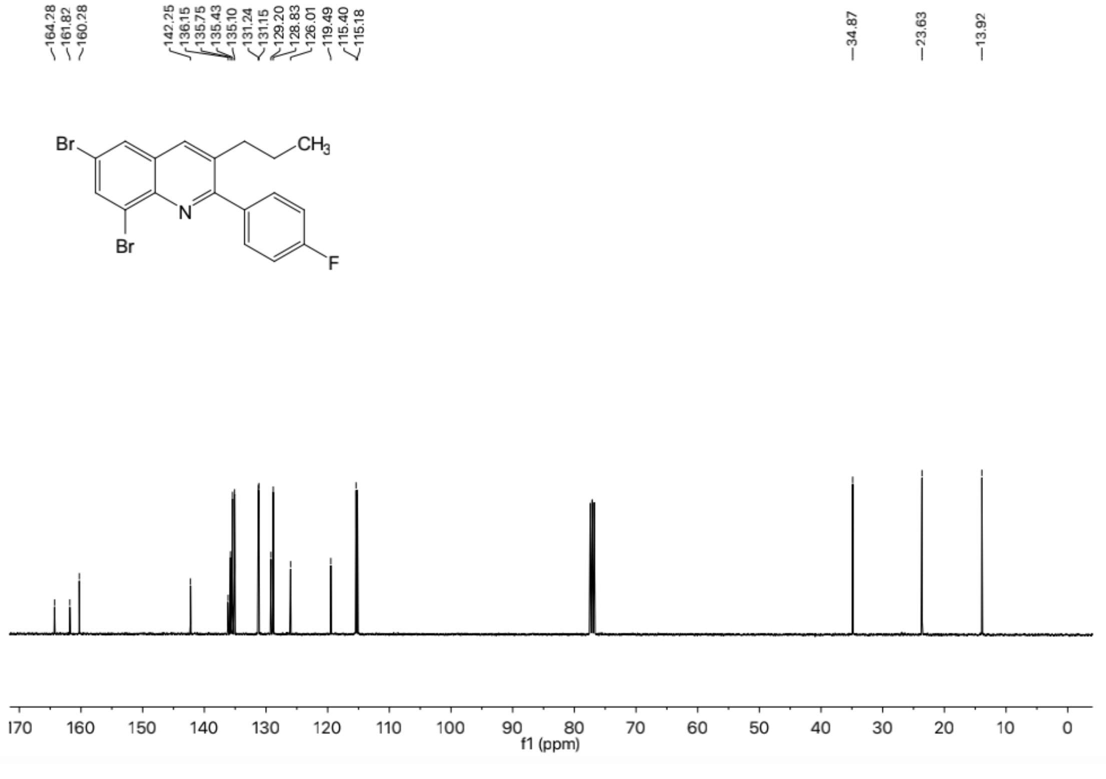

^{1}H NMR spectrum of 2-(2,4-dichlorophenyl)-3-propylquinoline（3ag）

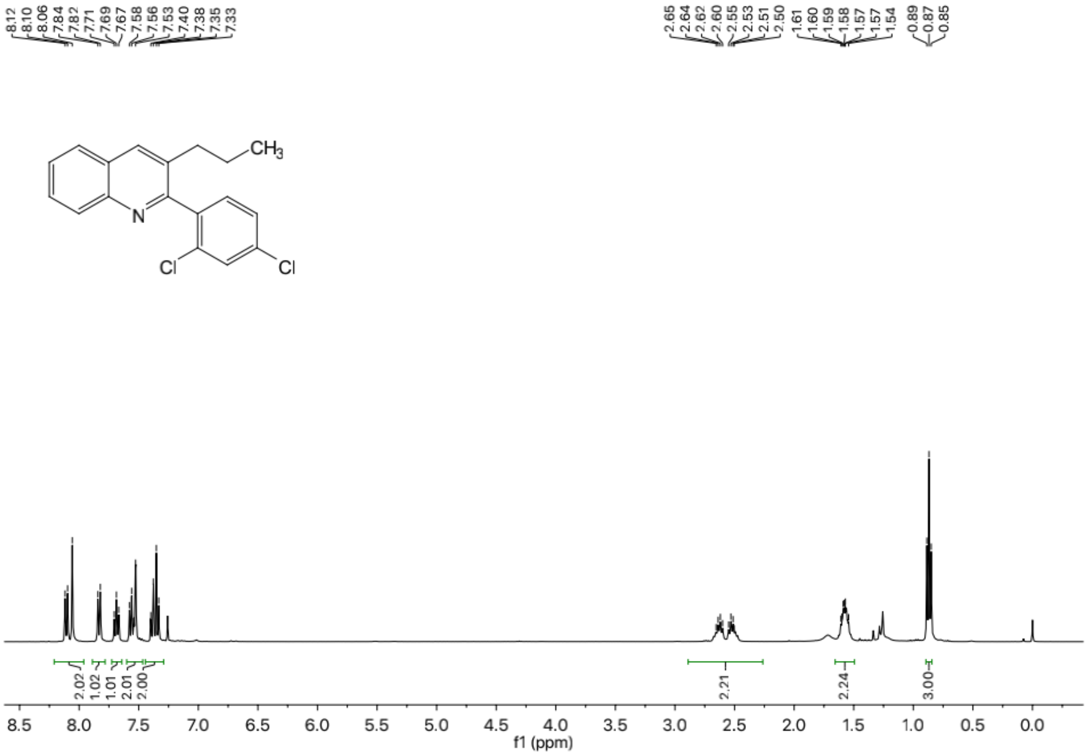

^{13}C NMR spectrum of 2-(2,4-dichlorophenyl)-3-propylquinoline（3ag）

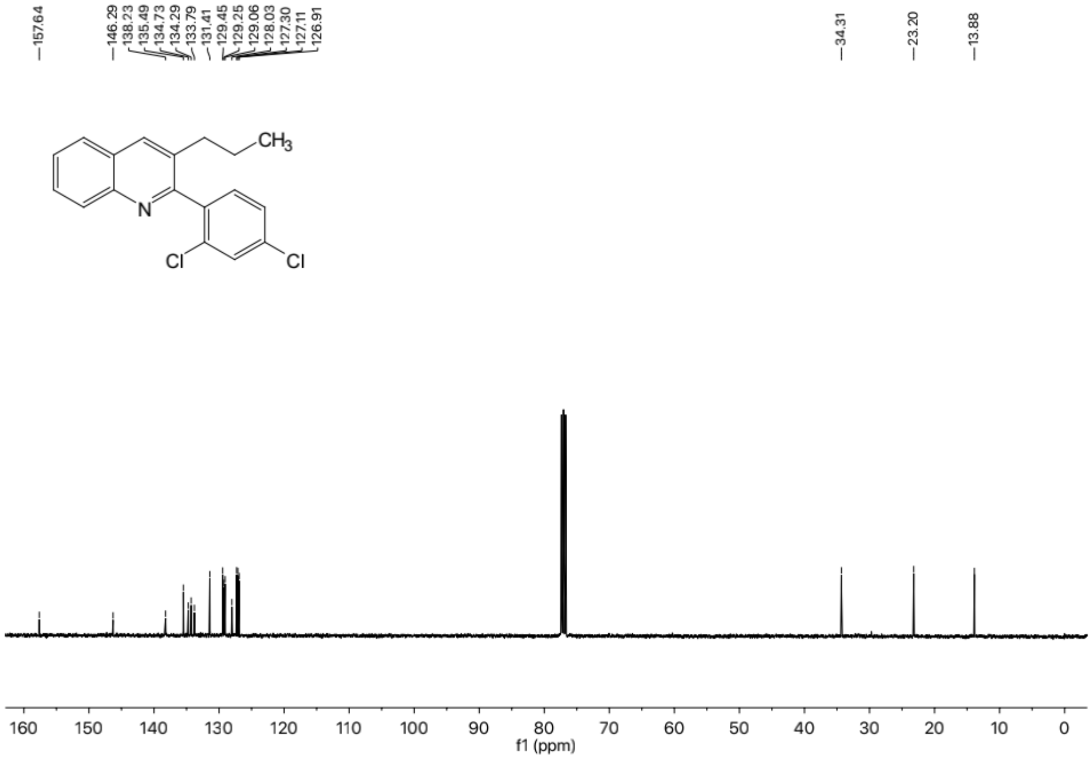

^{1}H NMR spectrum of 2-(2,4-dichlorophenyl)-8-methyl-3-propylquinoline（3bg）

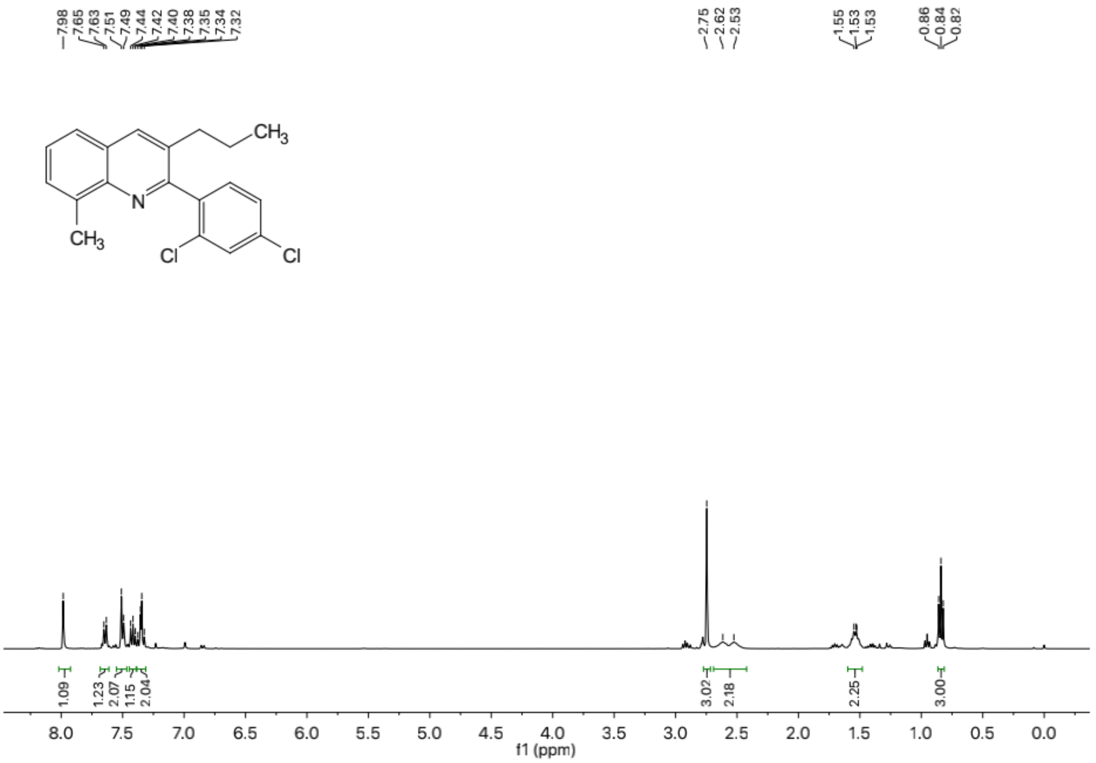

^{13}C NMR spectrum of 2-(2,4-dichlorophenyl)-8-methyl-3-propylquinoline（3bg）

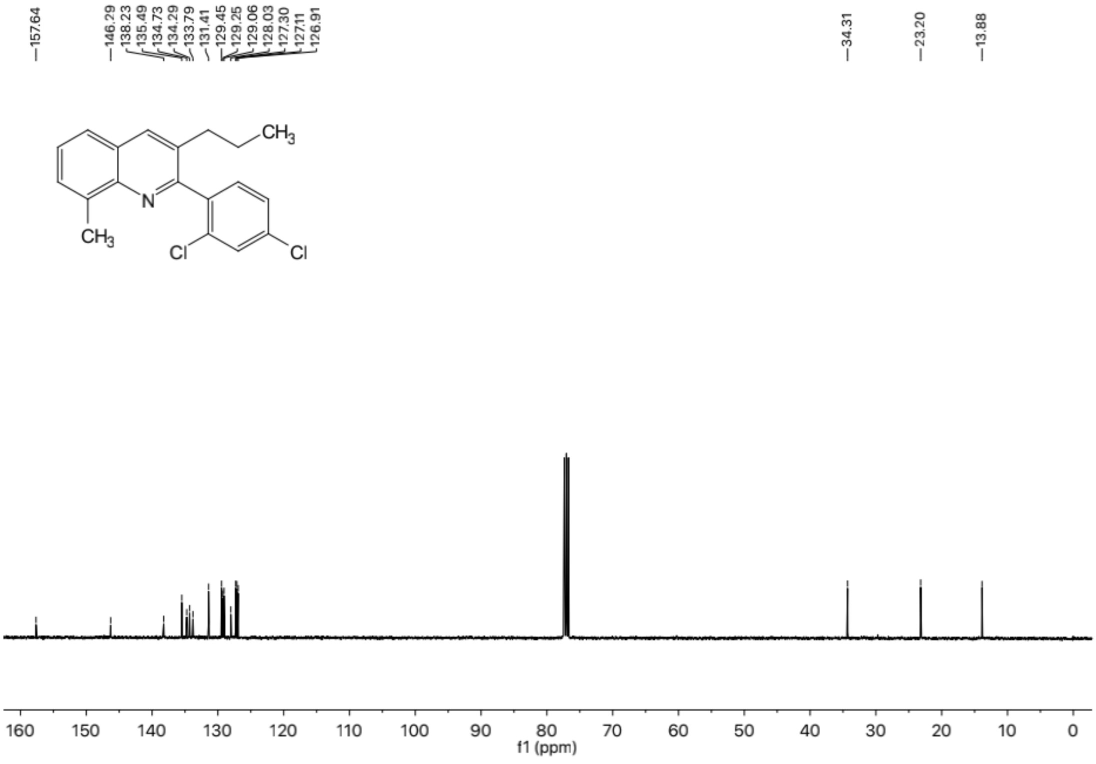

^{1}H NMR spectrum of 6-chloro-2-(2,4-dichlorophenyl)-3-propylquinoline（3cg）

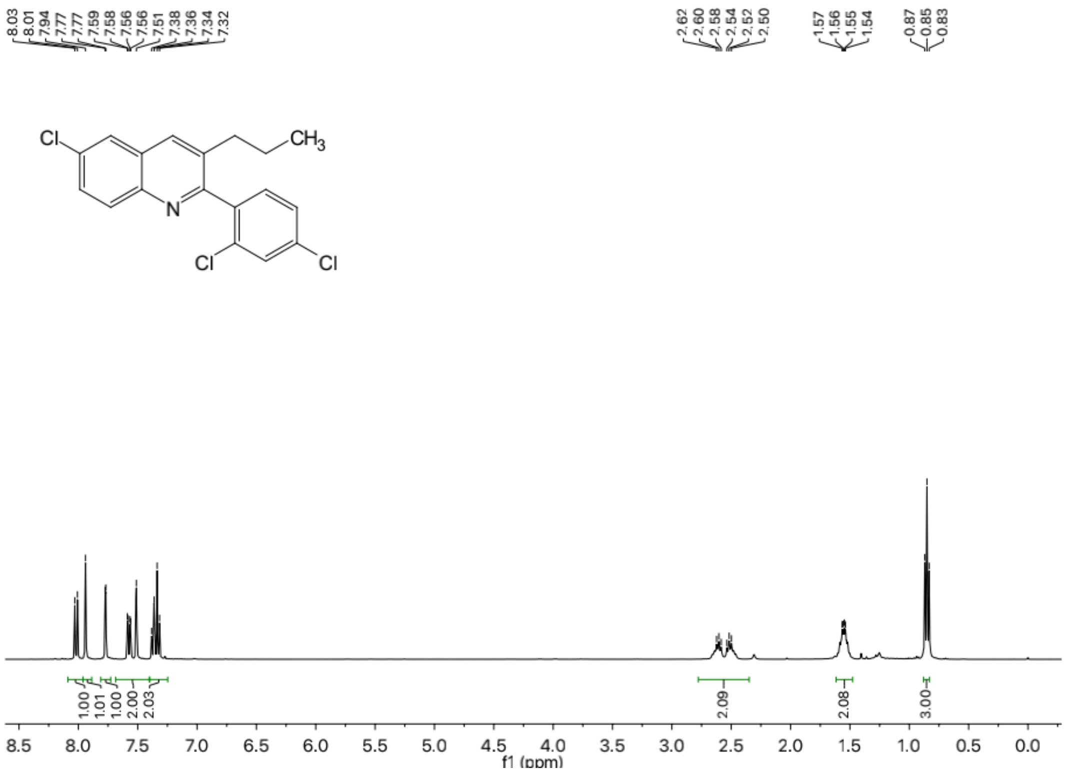

^{13}C NMR spectrum of 6-chloro-2-(2,4-dichlorophenyl)-3-propylquinoline（3cg）

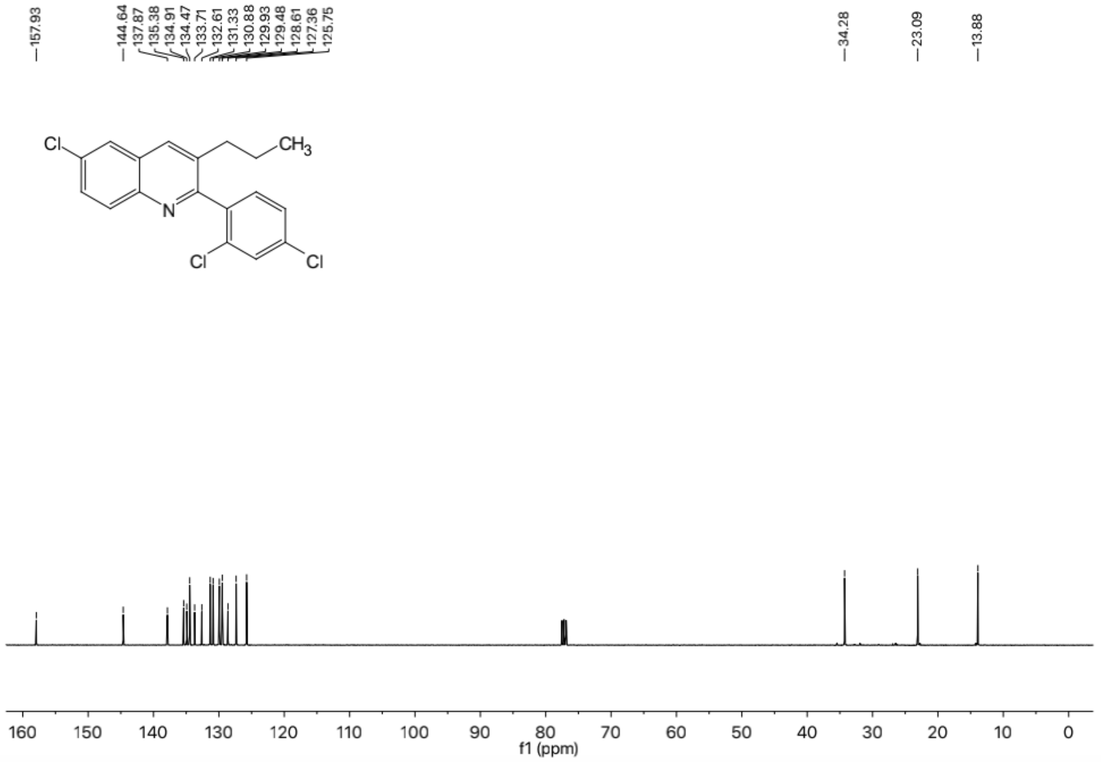

^{1}H NMR spectrum of 6,8-dibromo-2-(2,4-dichlorophenyl)-3-propylquinoline （3dg）

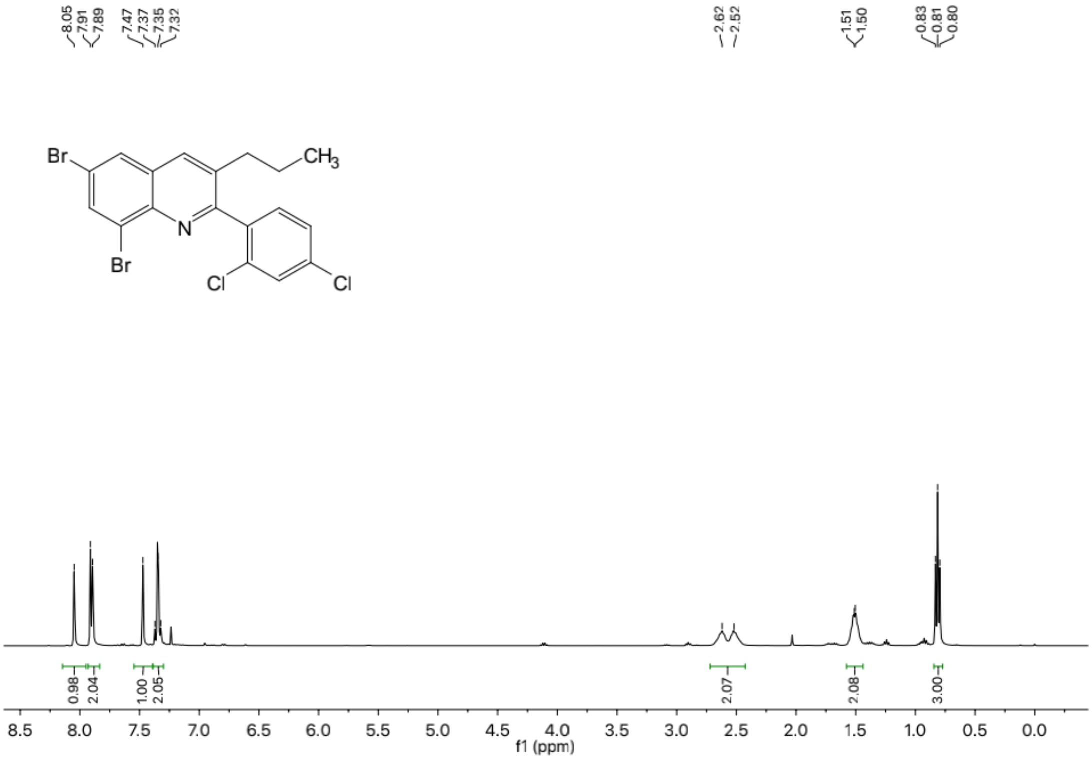

^{13}C NMR spectrum of 6,8-dibromo-2-(2,4-dichlorophenyl)-3-propylquinoline （3dg）

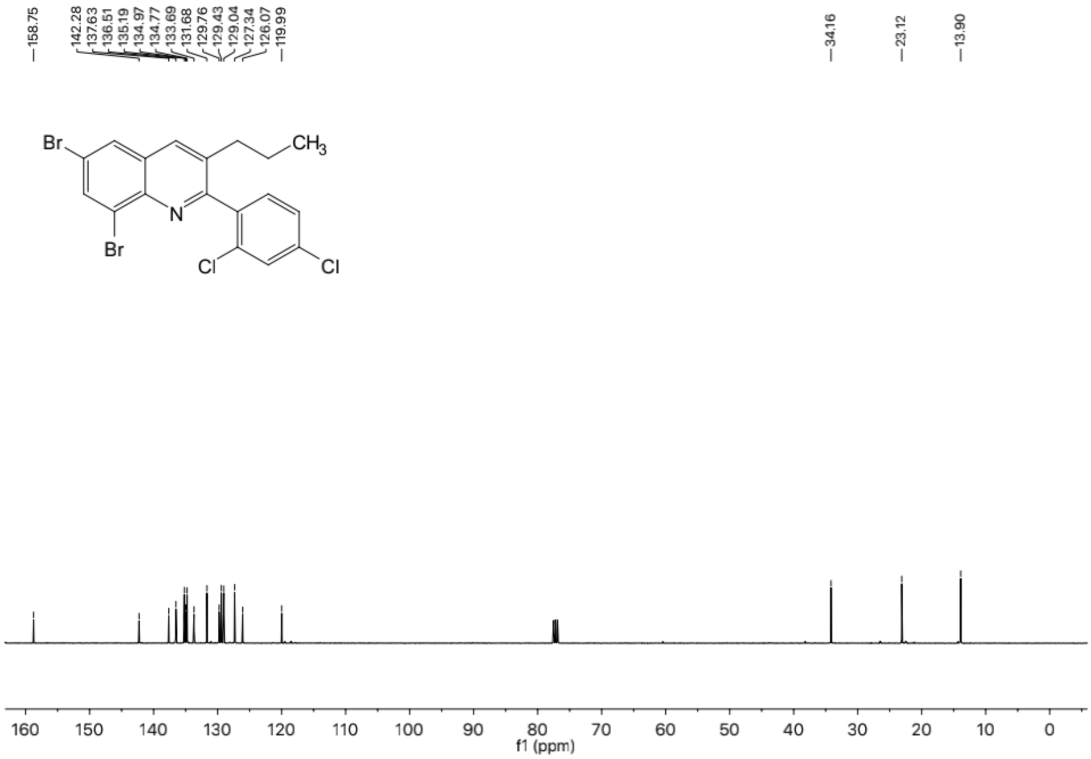

^{1}H NMR spectrum of 3-benzyl-2-(furan-2-yl)quinoline (3ha)

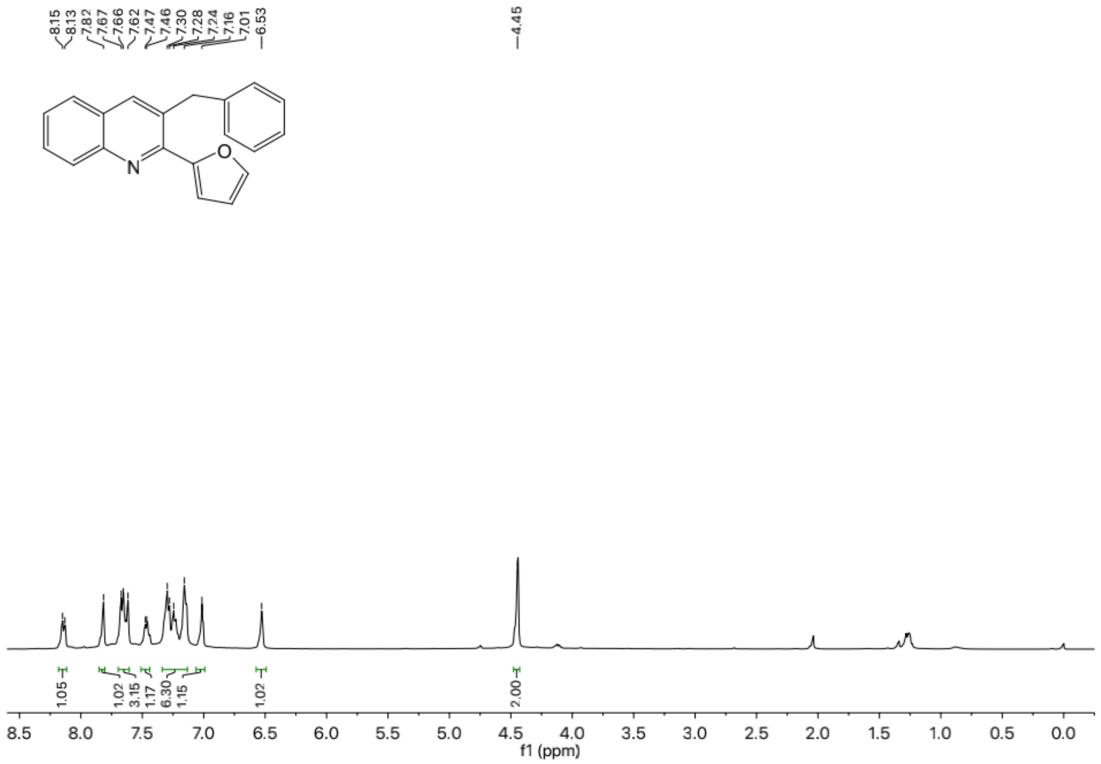

^{13}C NMR spectrum of 3-benzyl-2-(furan-2-yl)quinoline (3ha)

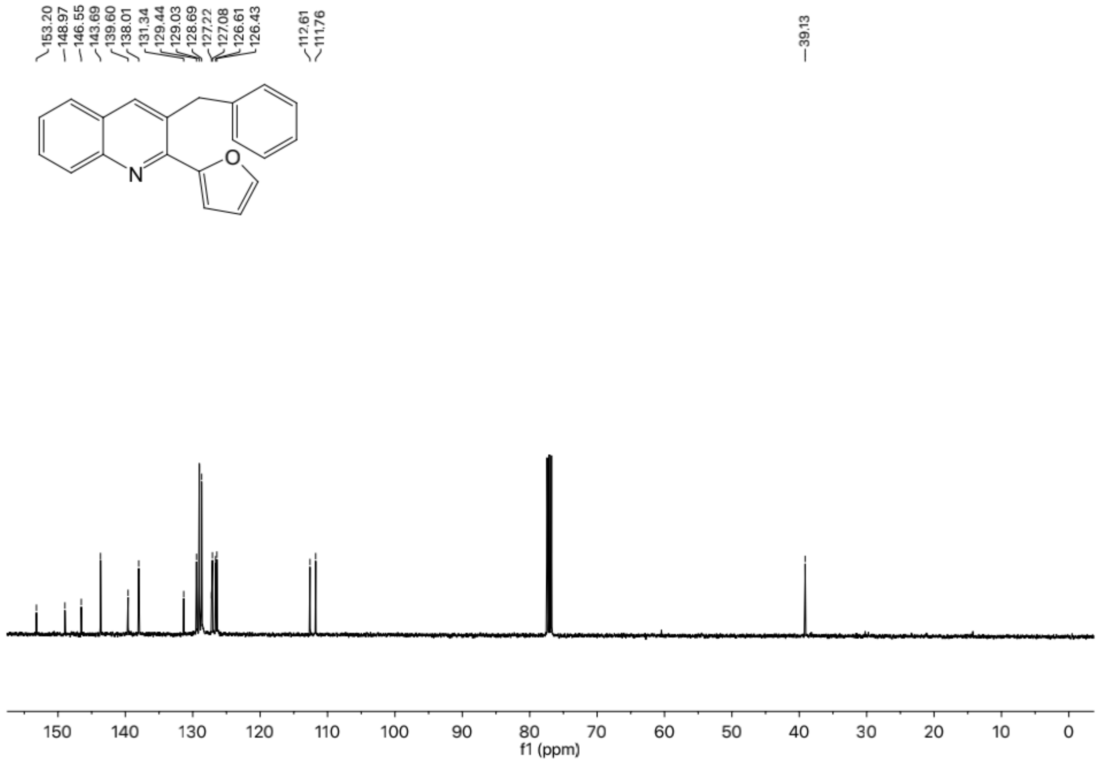

^{1}H NMR spectrum of 2-(furan-2-yl)quinoline (3ai)

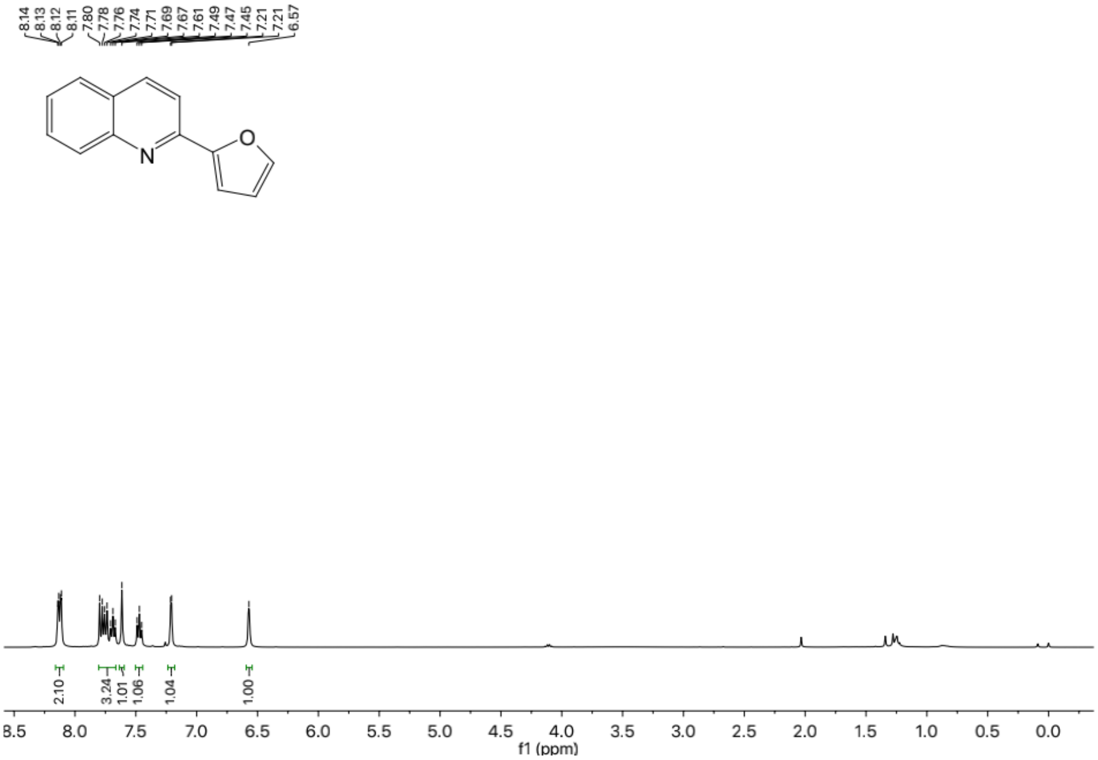

^{13}C NMR spectrum of 2-(furan-2-yl)quinoline (3ai)

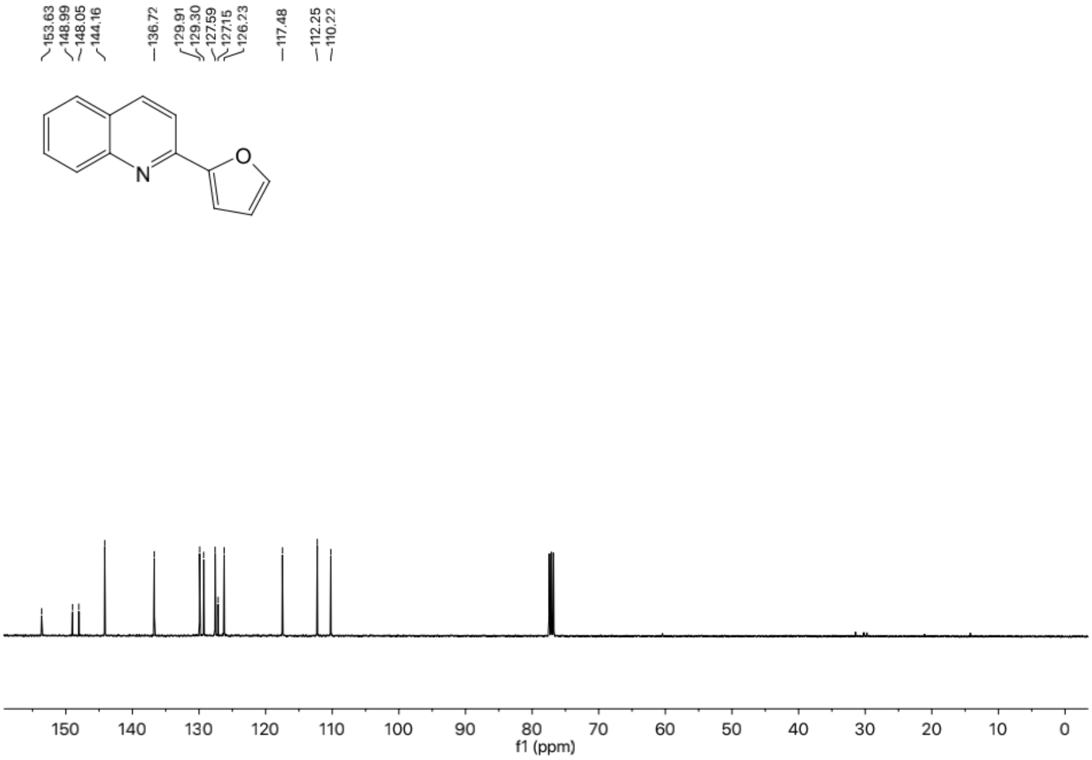

^{1}H NMR spectrum of 2-(furan-2-yl)-8-methylquinoline（3bi）

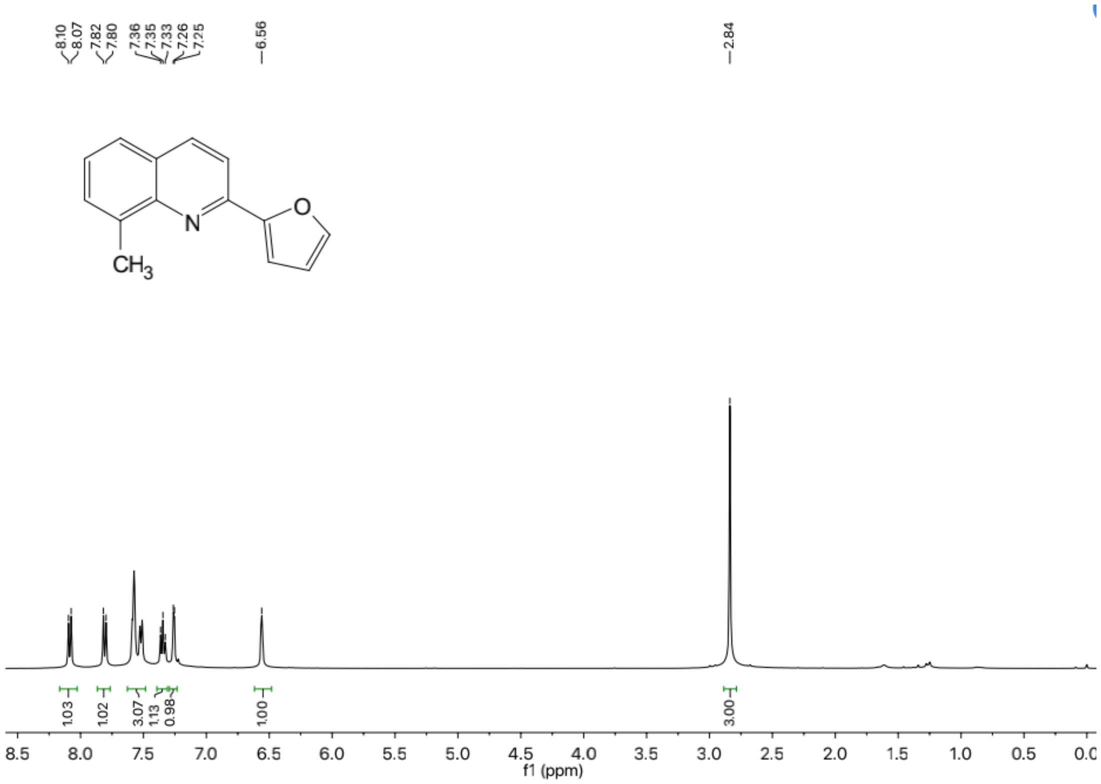

^{13}C NMR spectrum of 2-(furan-2-yl)-8-methylquinoline（3bi）

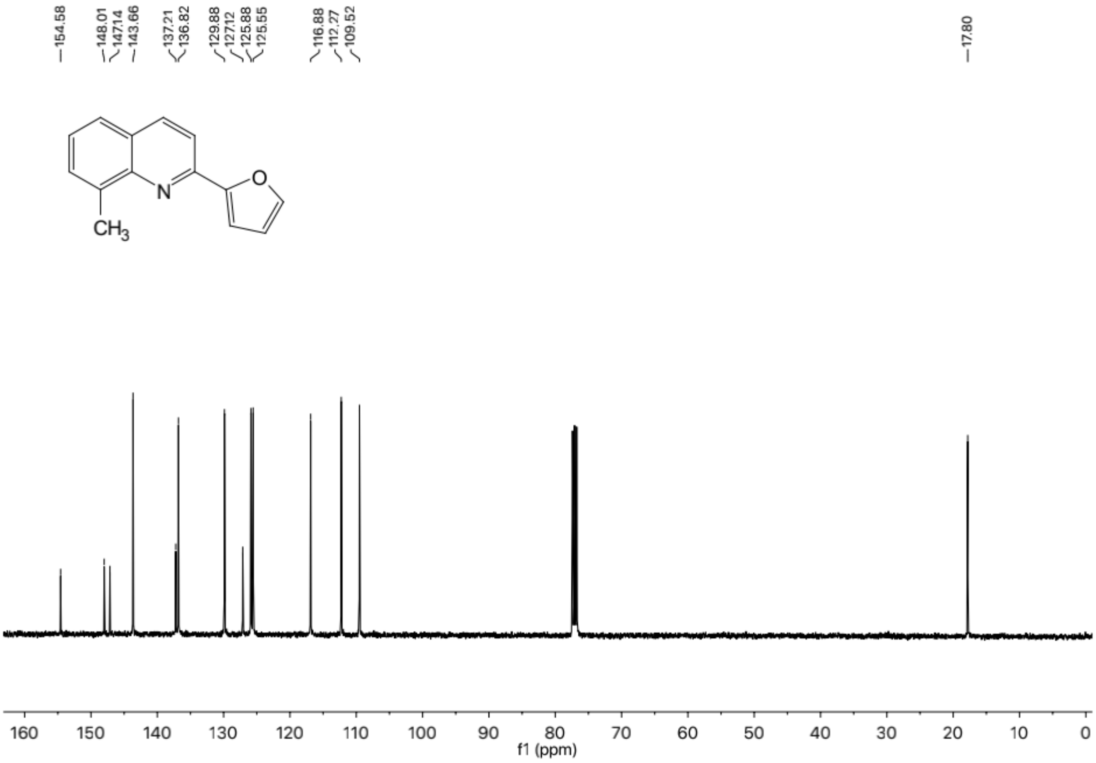

^{1}H NMR spectrum of 6-chloro-2-(furan-2-yl)quinoline（3ci）

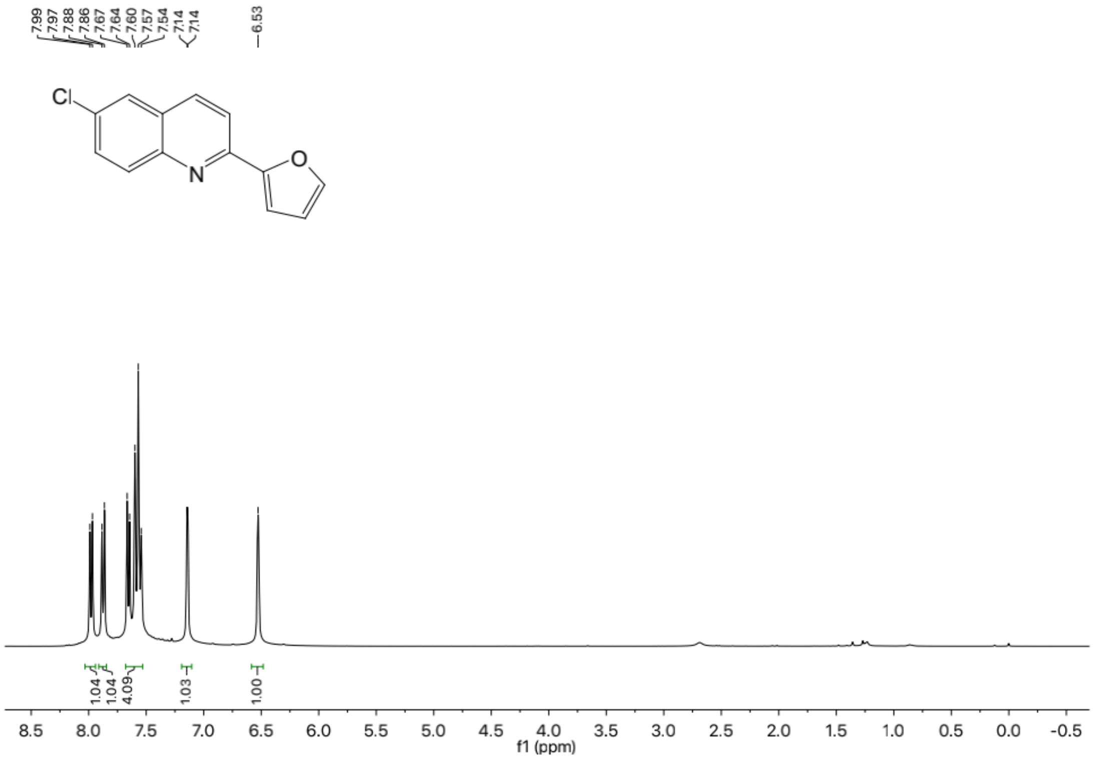

^{13}C NMR spectrum of 6-chloro-2-(furan-2-yl)quinoline（3ci）

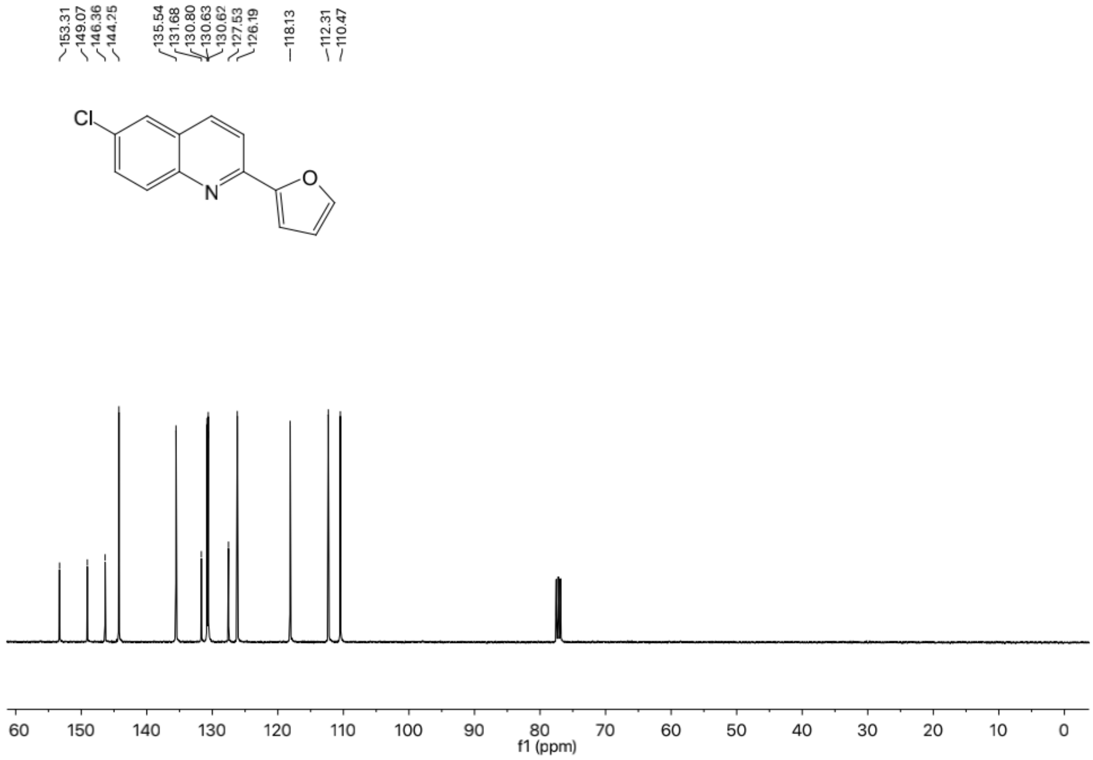

^1H NMR spectrum of 6,8-dibromo-2-(furan-2-yl)quinoline（3di）

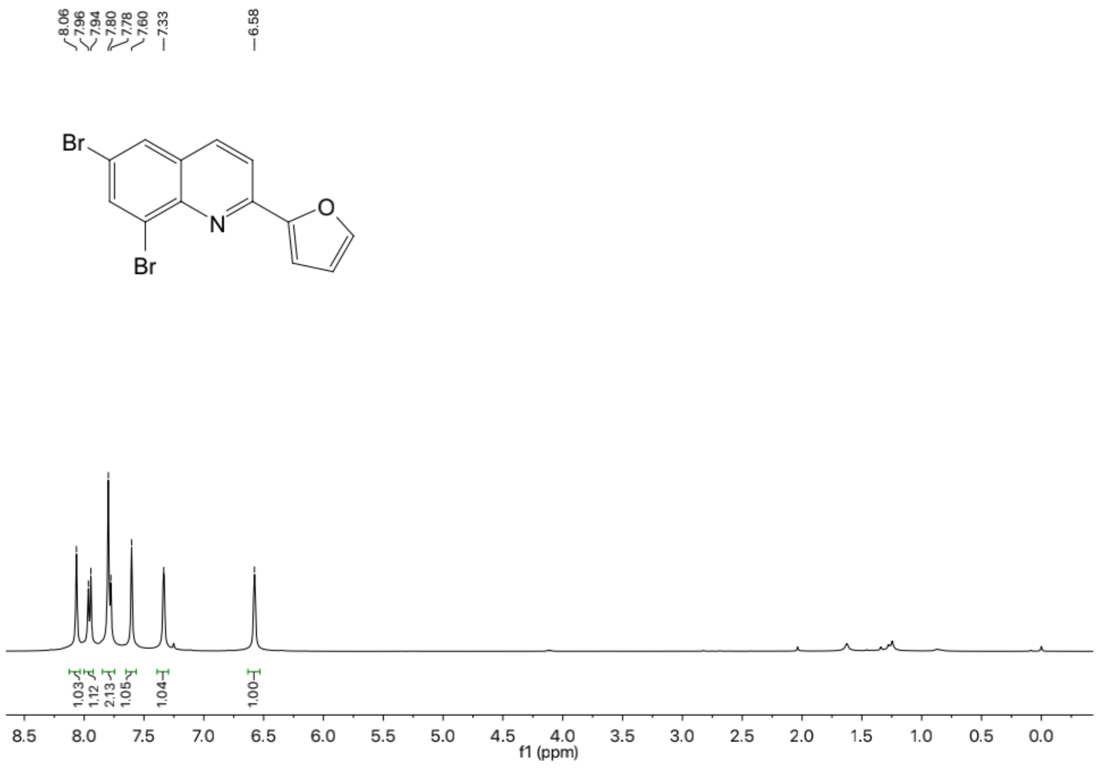

^{13}C NMR spectrum of 6,8-dibromo-2-(furan-2-yl)quinoline（3di）

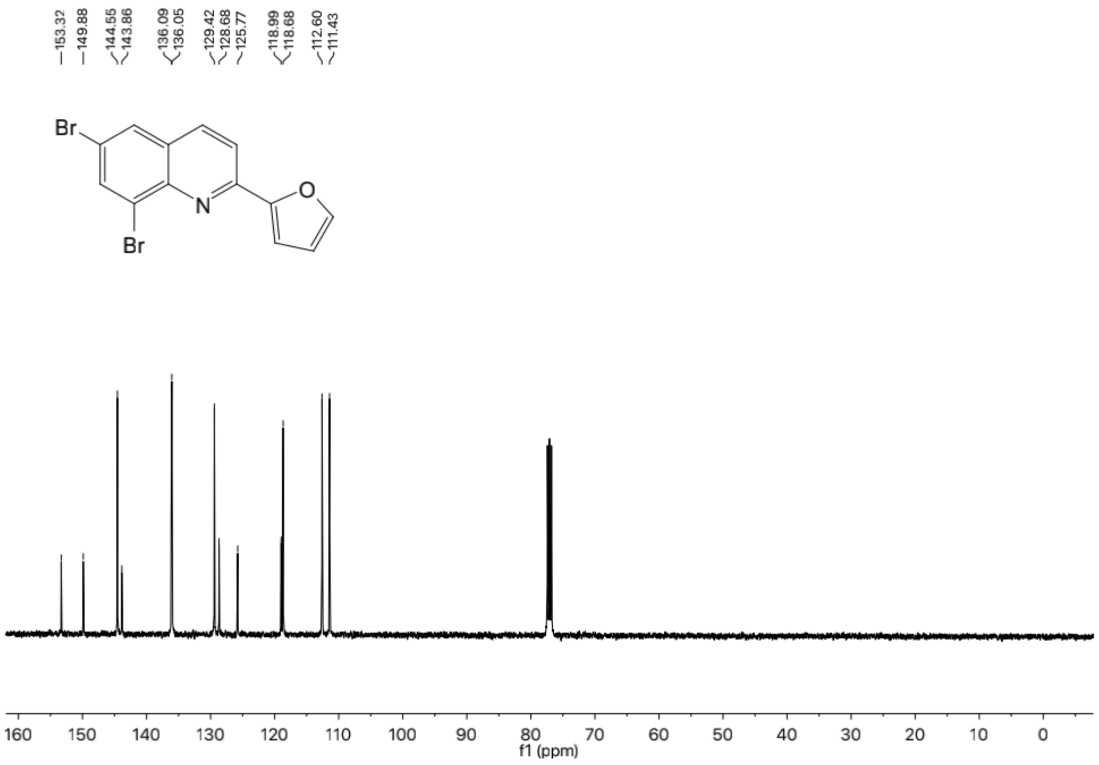

^{1}H NMR spectrum of 2-(5-methylfuran-2-yl)quinoline（3aj）

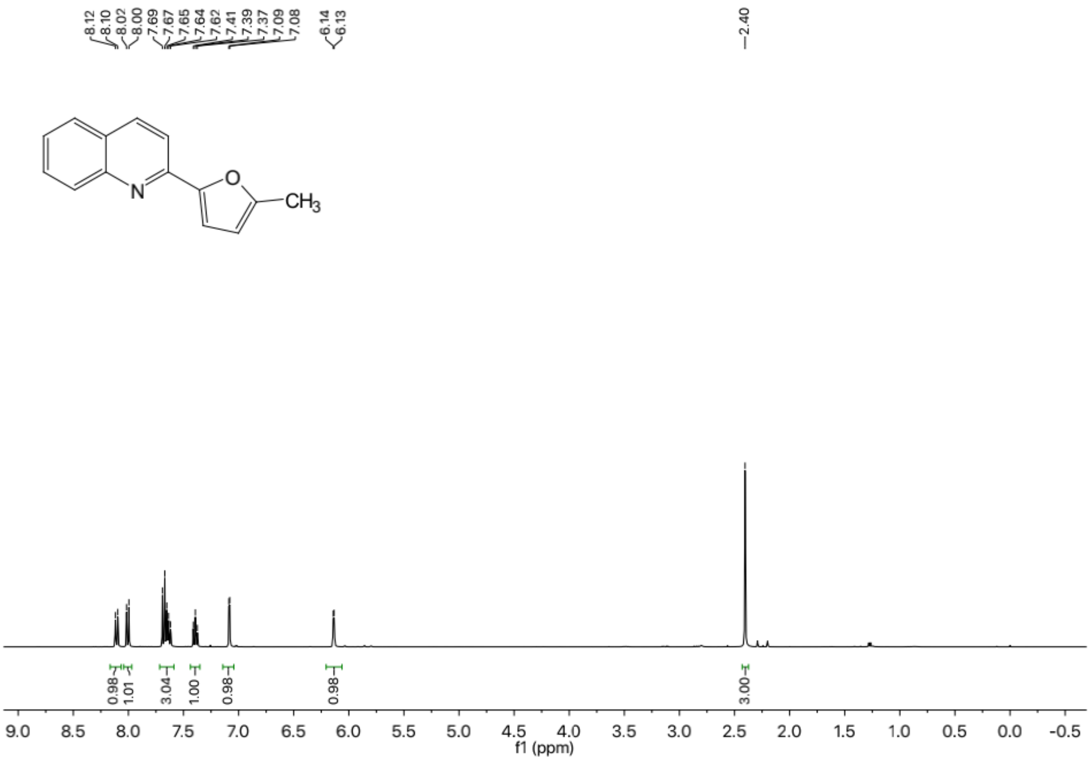

^{13}C NMR spectrum of 2-(5-methylfuran-2-yl)quinoline（3aj）

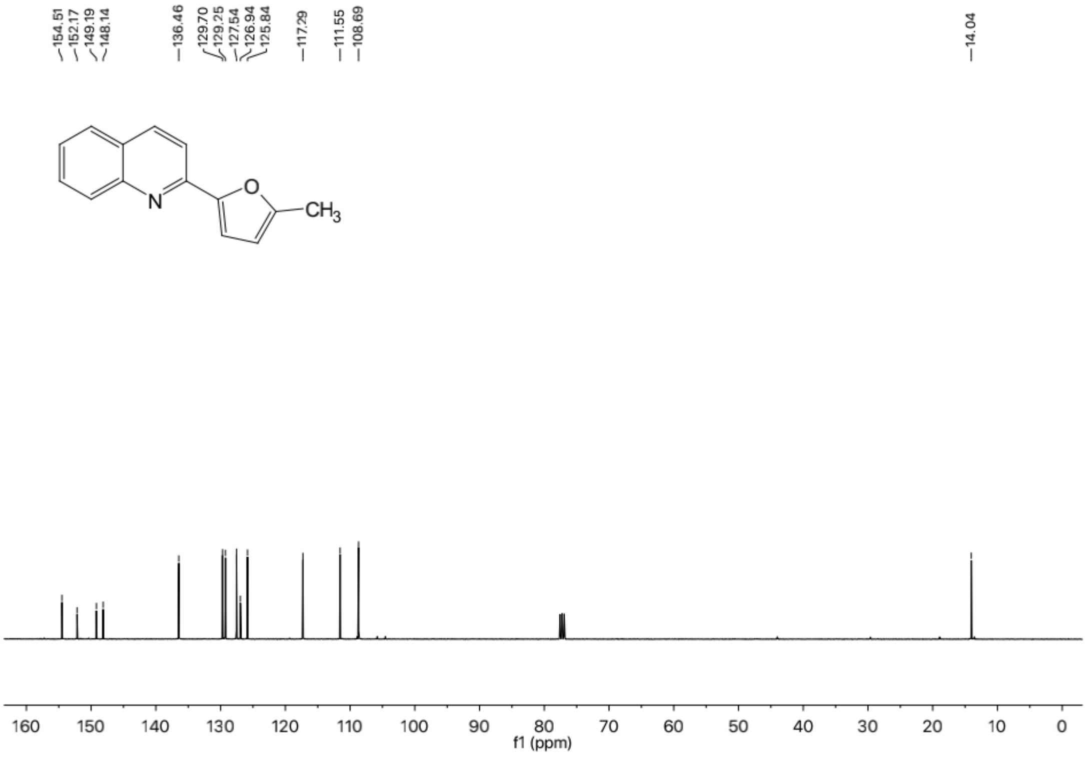

^{1}H NMR spectrum of 8-methyl-2-(5-methylfuran-2-yl)quinoline（3bj）

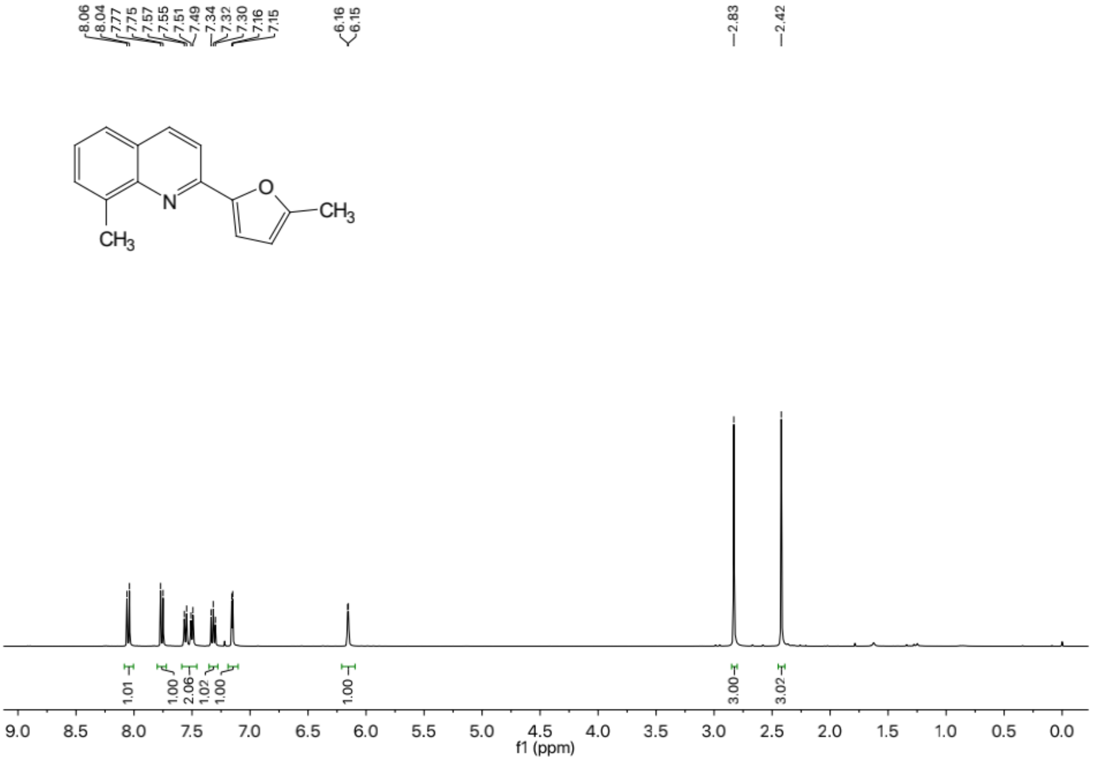

^{13}C NMR spectrum of 8-methyl-2-(5-methylfuran-2-yl)quinoline（3bj）

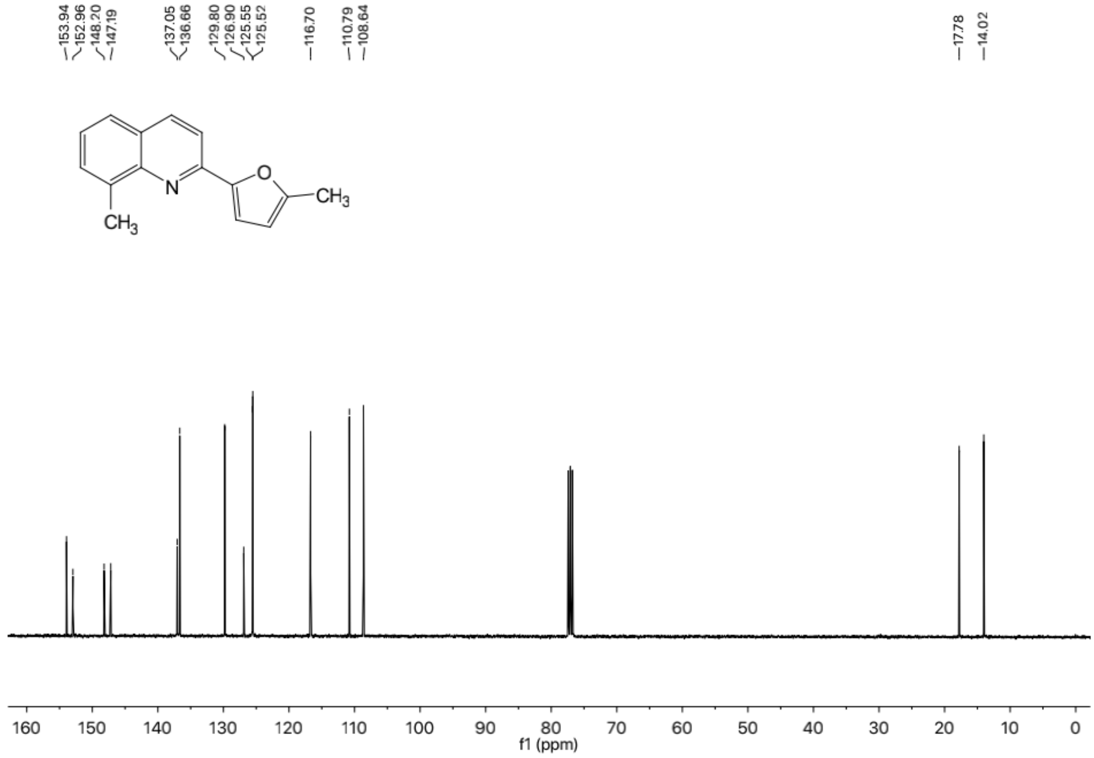

^{1}H NMR spectrum of 6-chloro-2-(5-methylfuran-2-yl)quinoline（3cj）

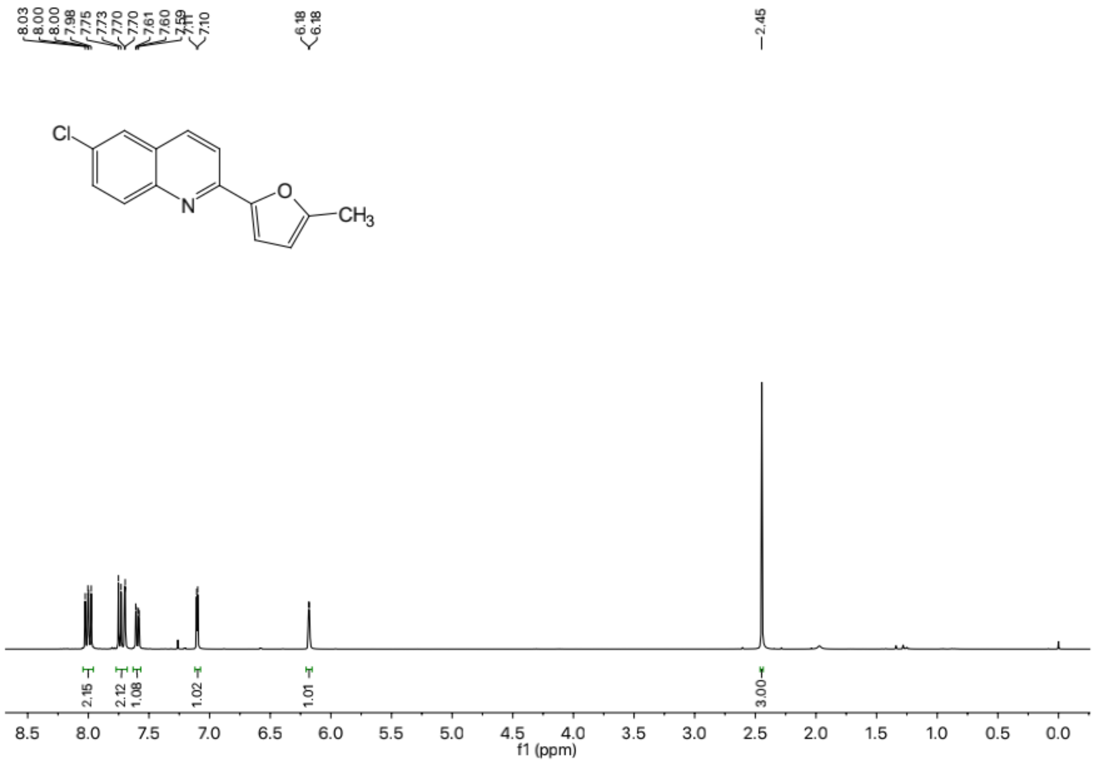

^{13}C NMR spectrum of 6-chloro-2-(5-methylfuran-2-yl)quinoline（3cj）

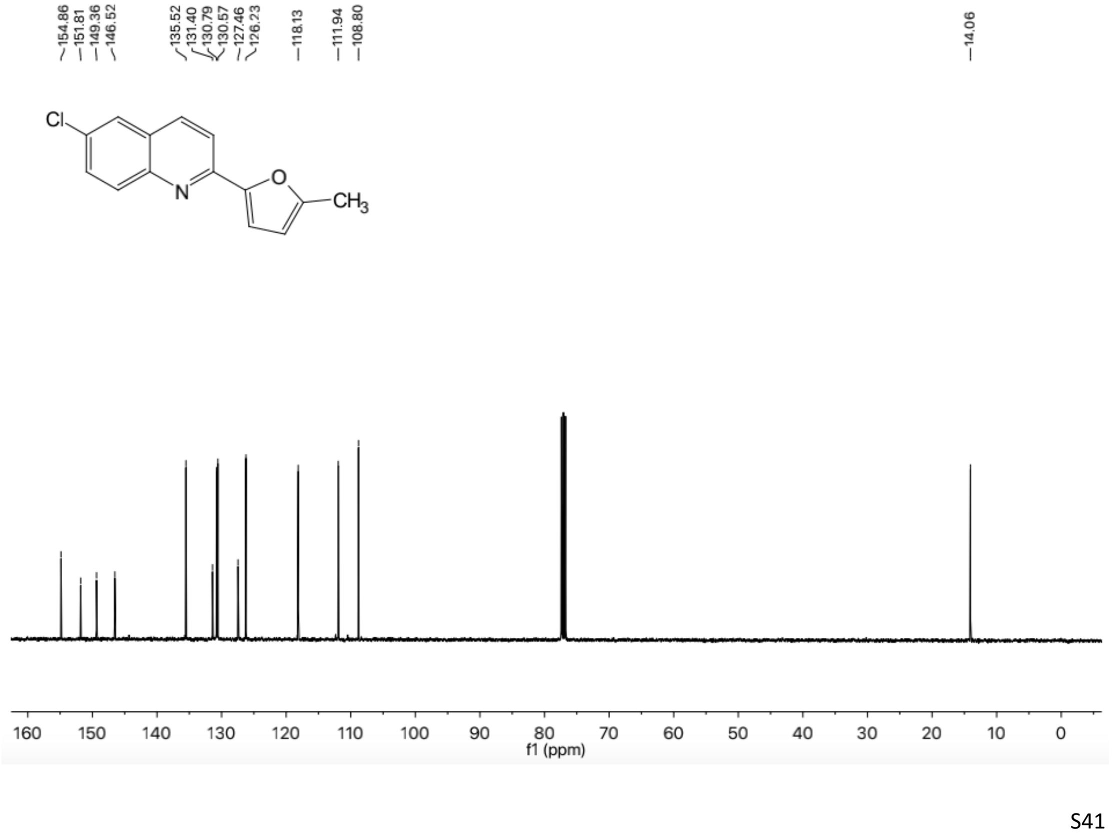

^{1}H NMR spectrum of 6,8-dibromo-2-(5-methylfuran-2-yl)quinoline（3dj）

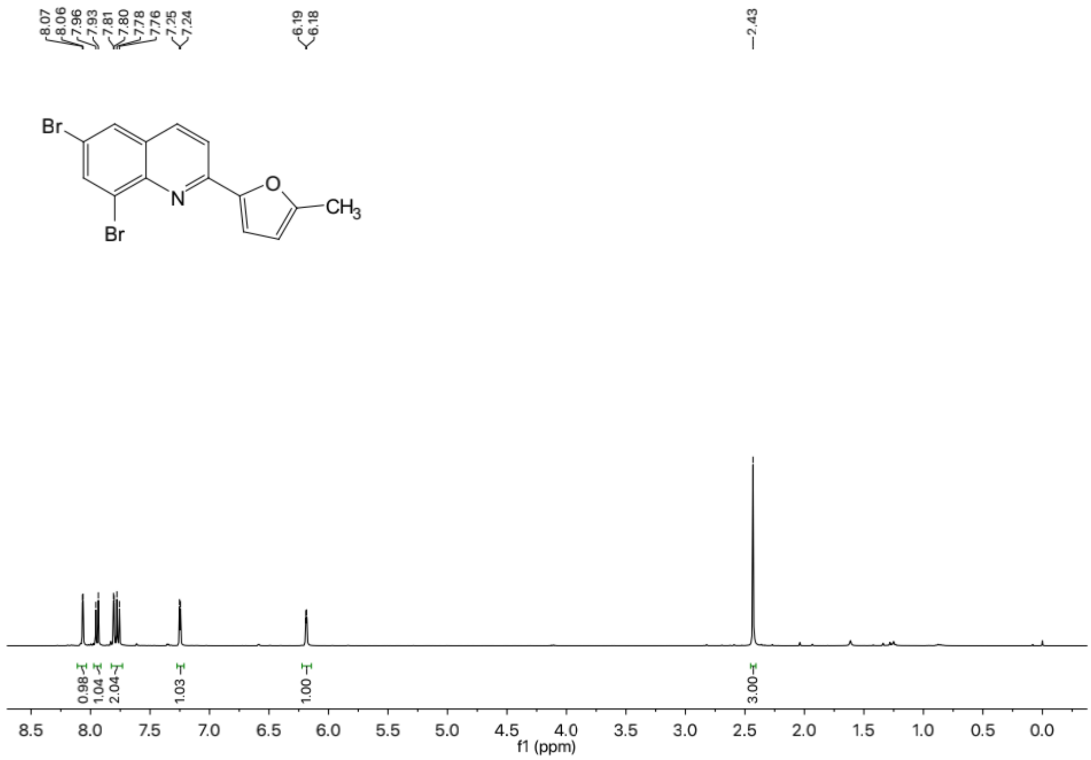

^{13}C NMR spectrum of 6,8-dibromo-2-(5-methylfuran-2-yl)quinoline（3dj）

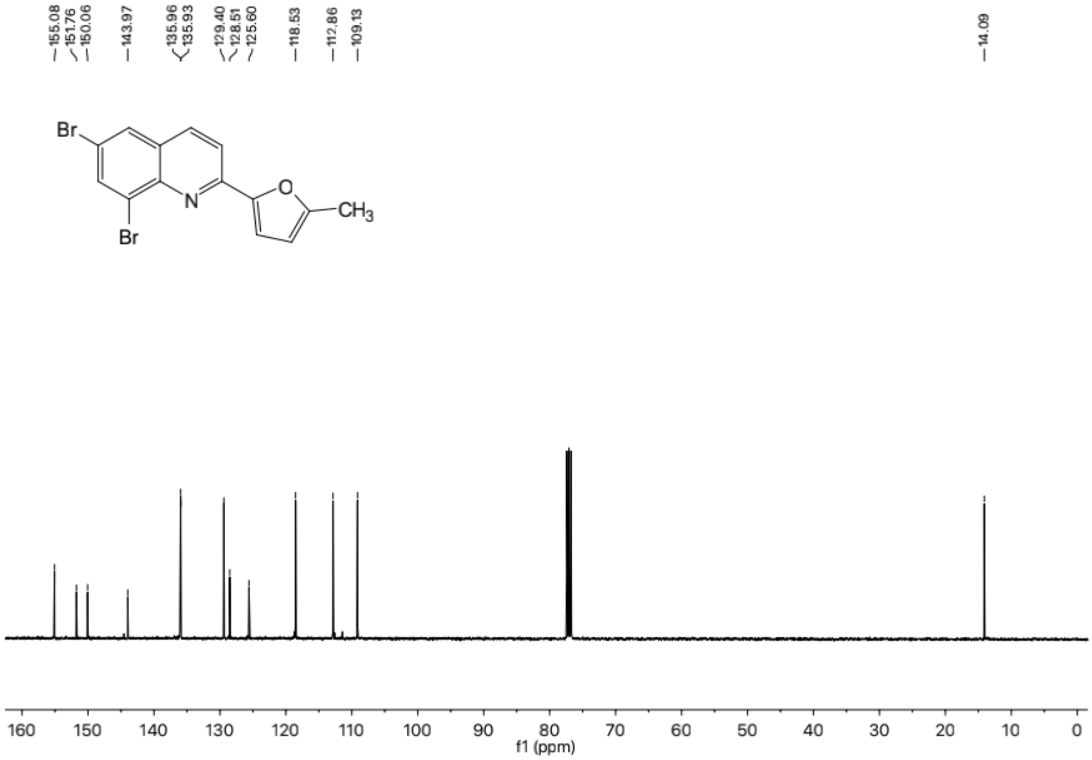

^{1}H NMR spectrum of 3-methyl-2-(5-methylfuran-2-yl)quinoline（3ak）

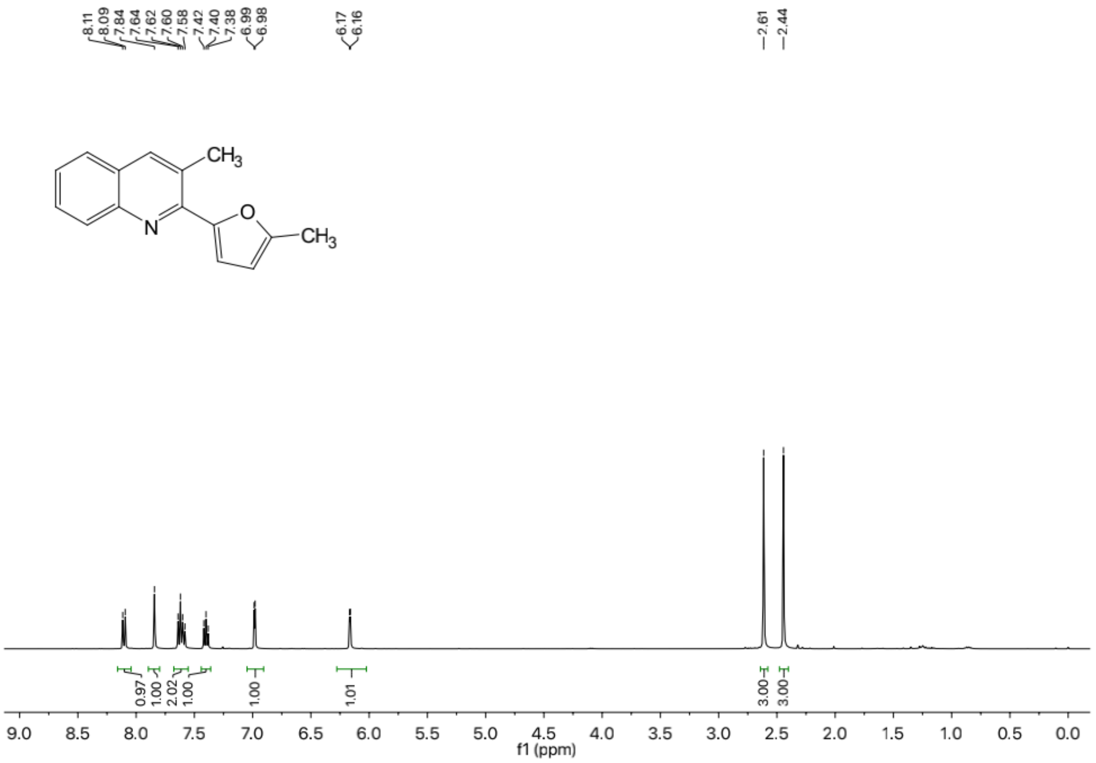

^{13}C NMR spectrum of 3-methyl-2-(5-methylfuran-2-yl)quinoline（3ak）

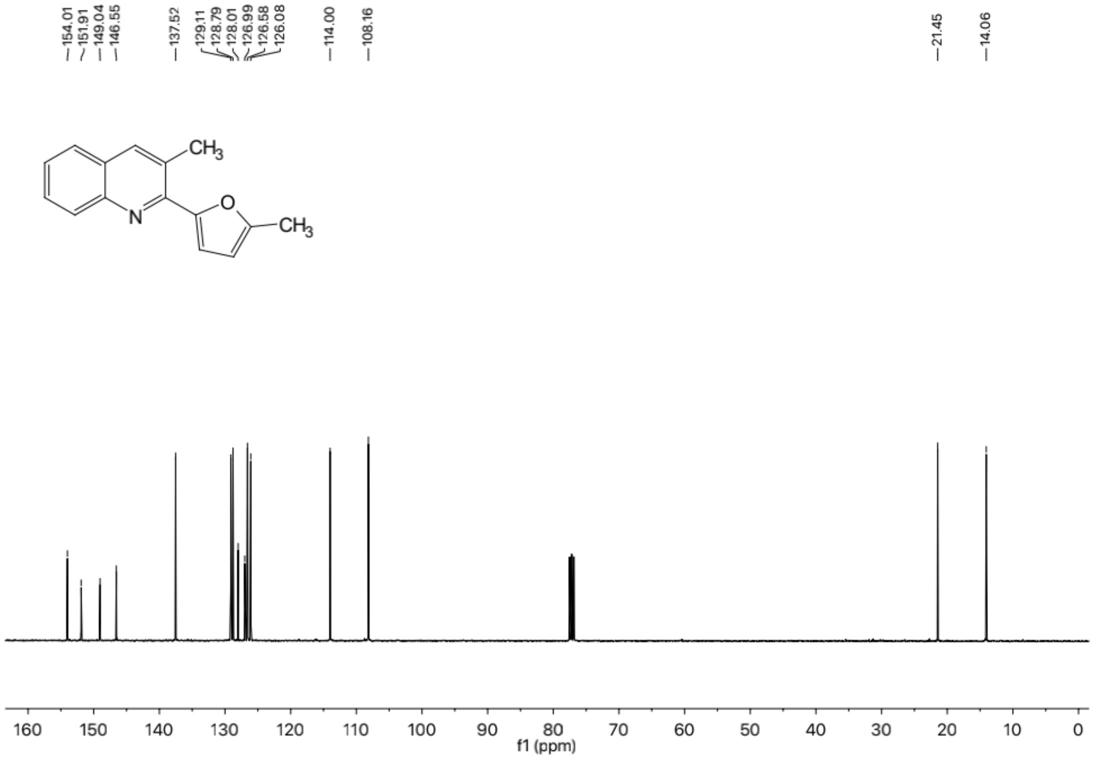

^{1}H NMR spectrum of 3,8-dimethyl-2-(5-methylfuran-2-yl)quinoline（3bk）

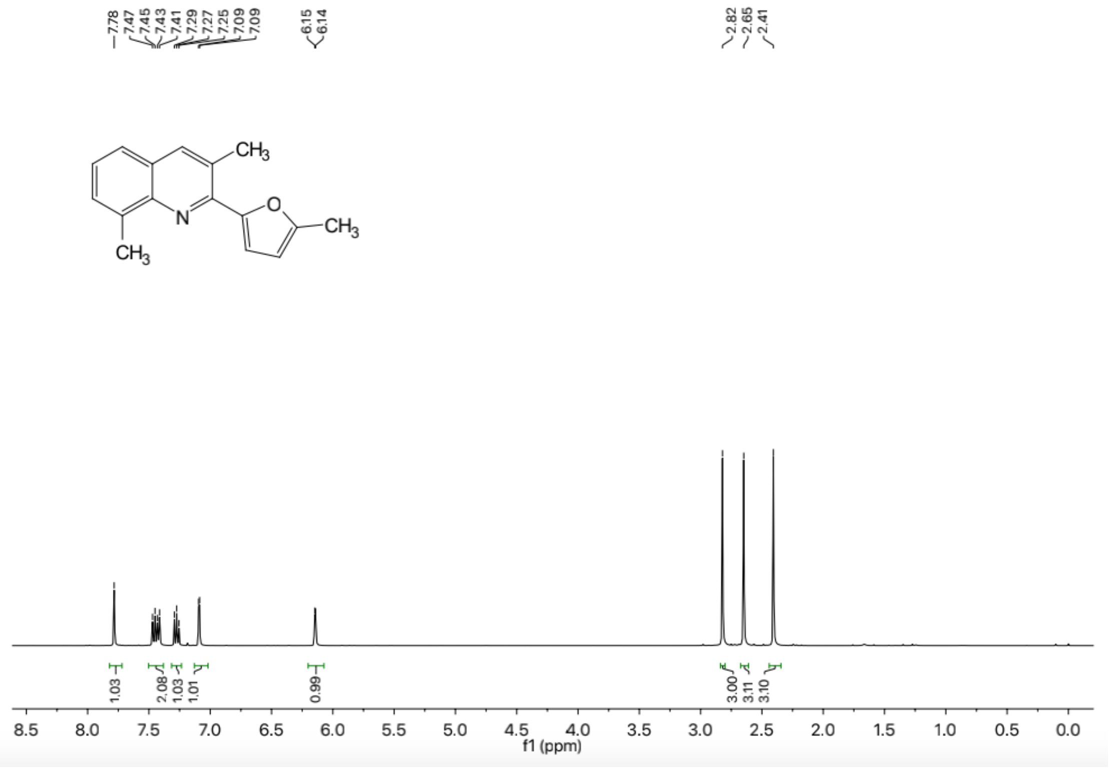

^{13}C NMR spectrum of 3,8-dimethyl-2-(5-methylfuran-2-yl)quinoline（3bk）

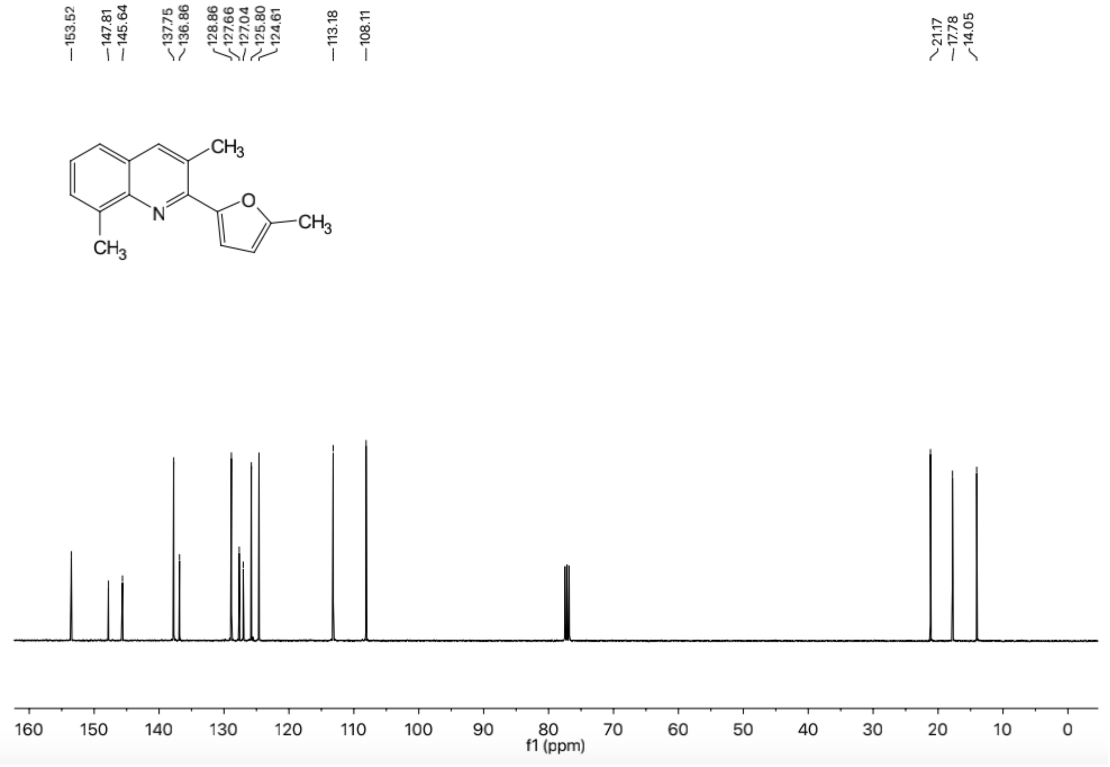

^{1}H NMR spectrum of 6-chloro-3-methyl-2-(5-methylfuran-2-yl)quinoline（3ck）

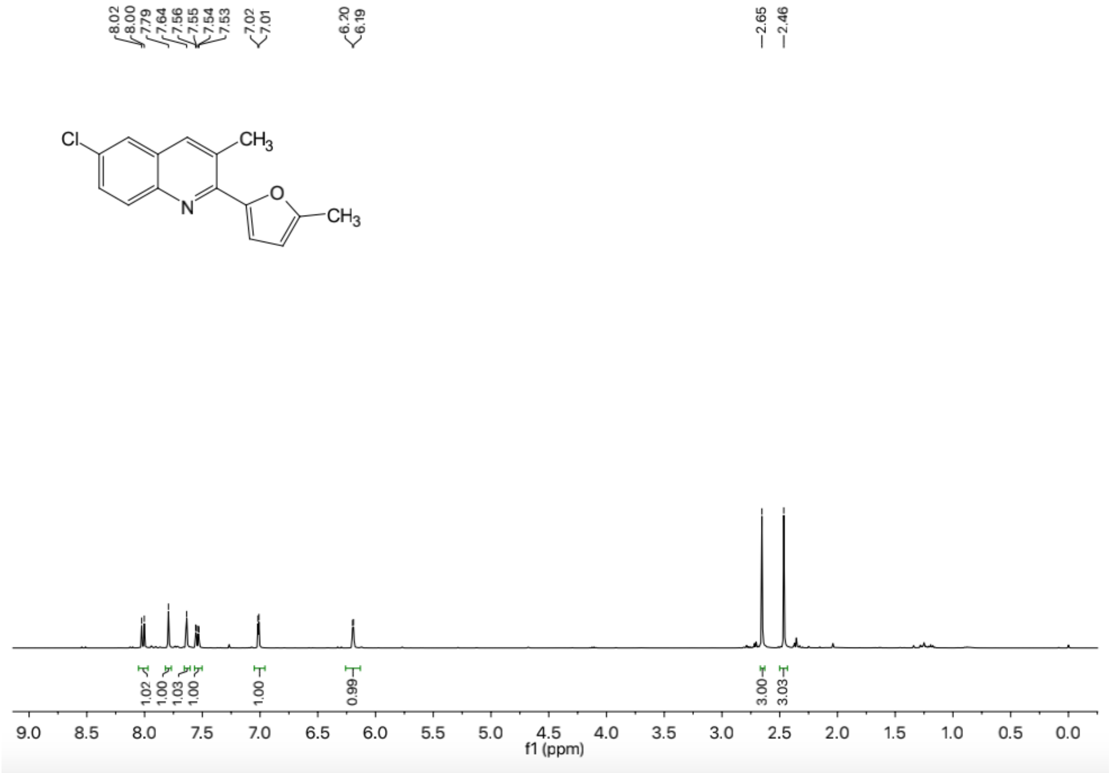

^{13}C NMR spectrum of 6-chloro-3-methyl-2-(5-methylfuran-2-yl)quinoline

（3ck）

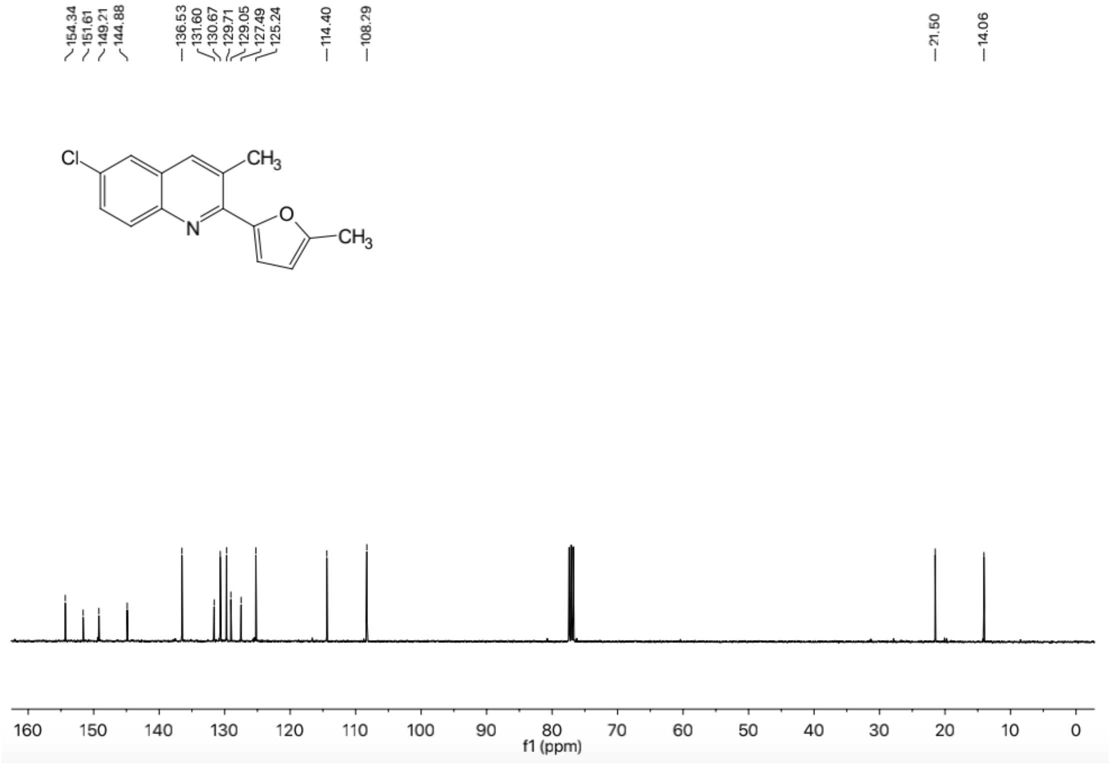

^{1}H NMR spectrum of 6,8-dibromo-3-methyl-2-(5-methylfuran-2-yl)quinoline (3dk)

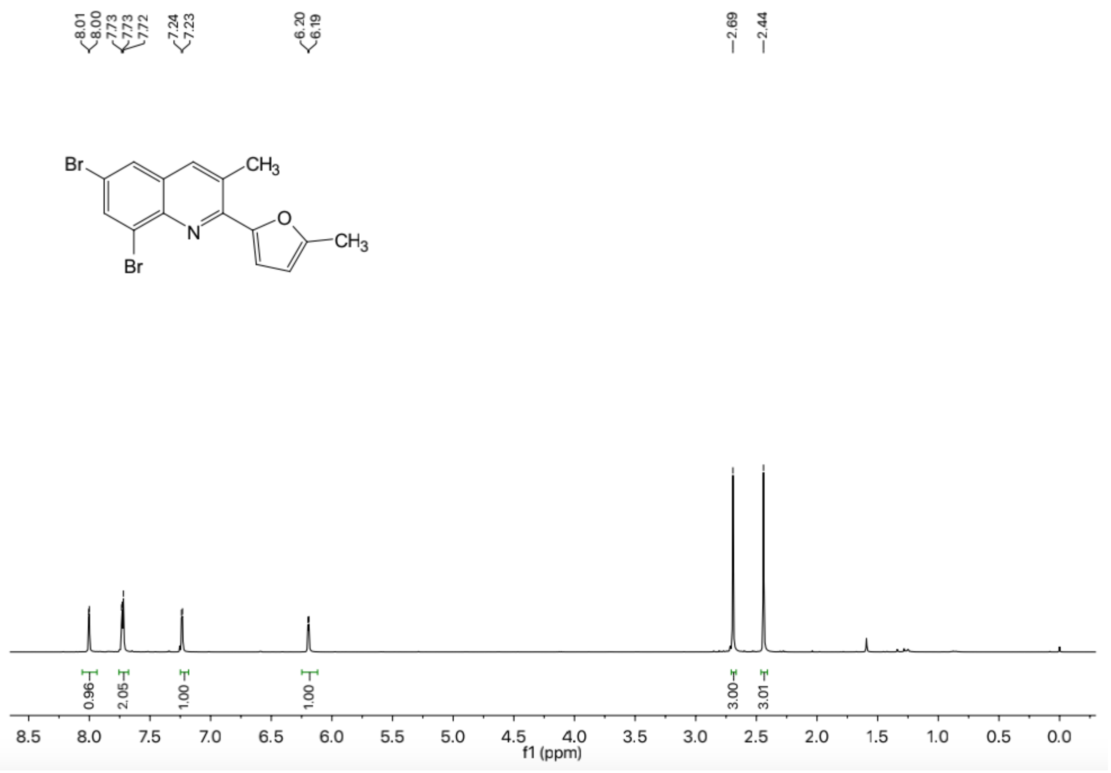

^{13}C NMR spectrum of 6,8-dibromo-3-methyl-2-(5-methylfuran-2-yl)quinoline (3dk)

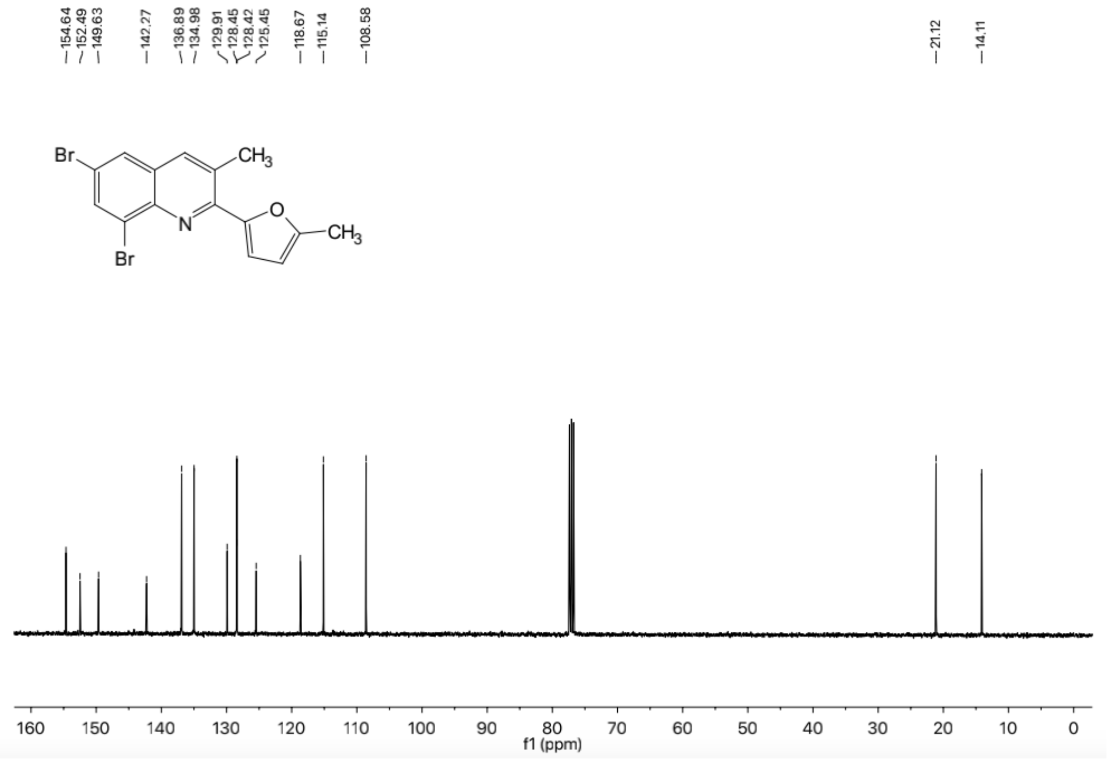

^{1}H NMR spectrum of 2-(thiophen-2-yl)quinoline（3al）

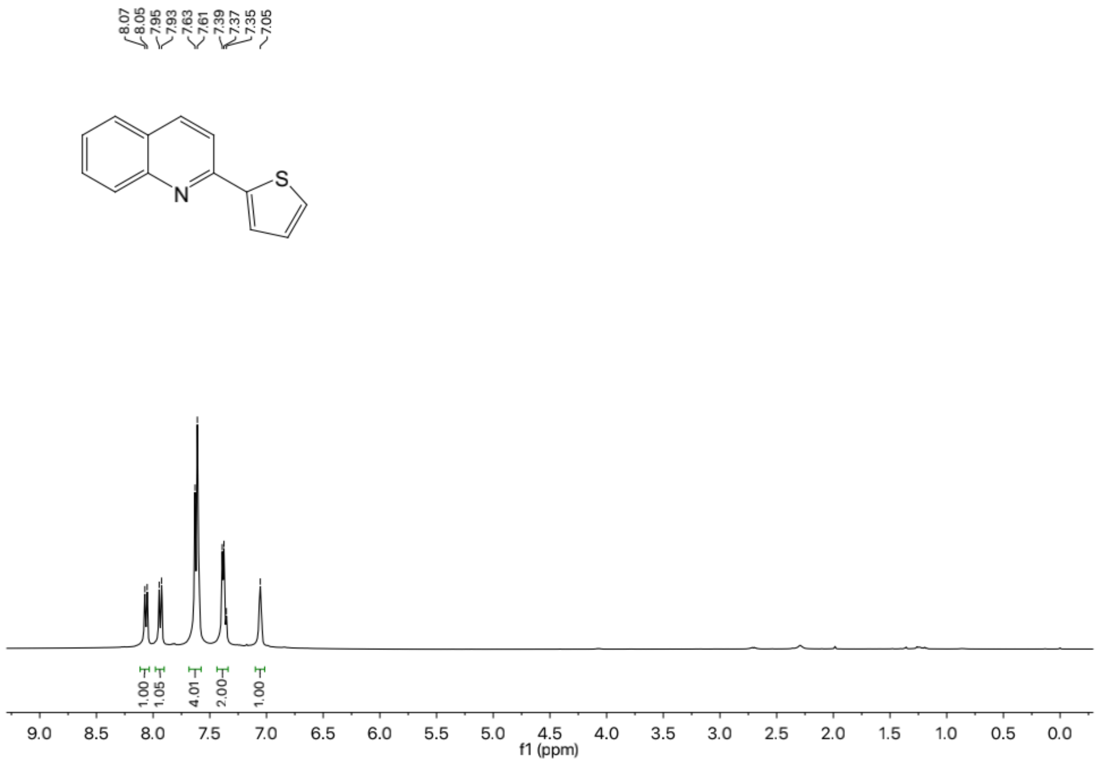

^{13}C NMR spectrum of 2-(thiophen-2-yl)quinoline（3al）

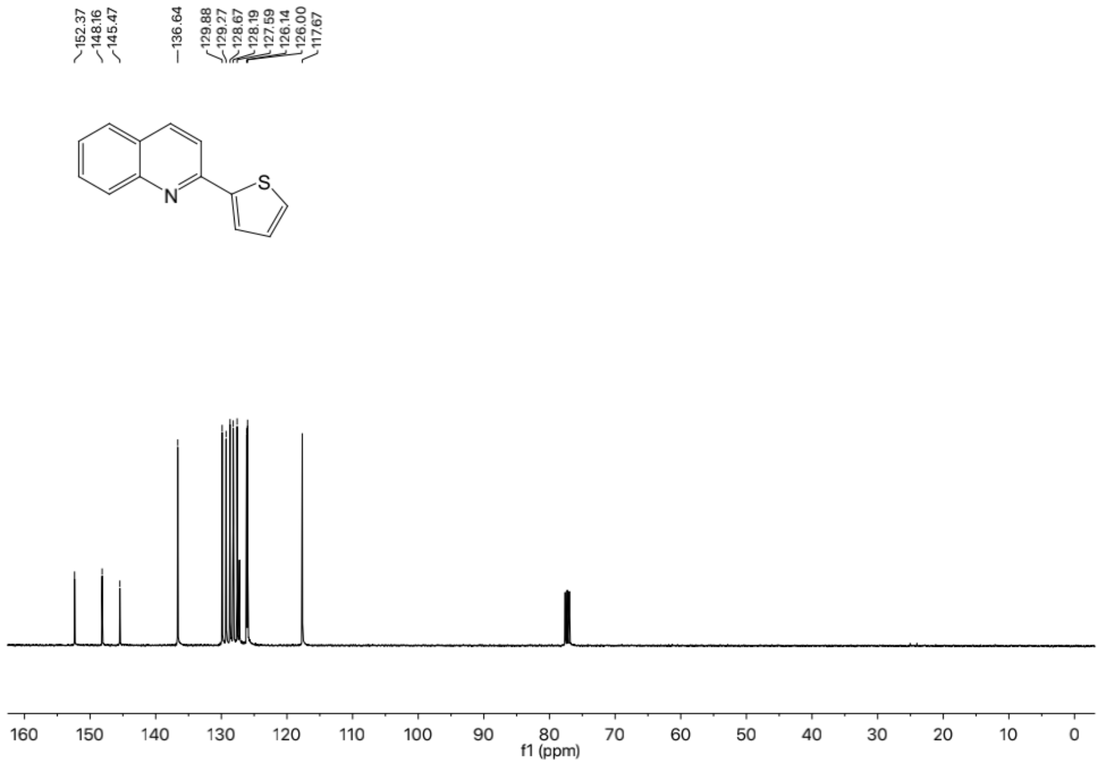

^{1}H NMR spectrum of 8-methyl-2-(thiophen-2-yl)quinoline（3bl）

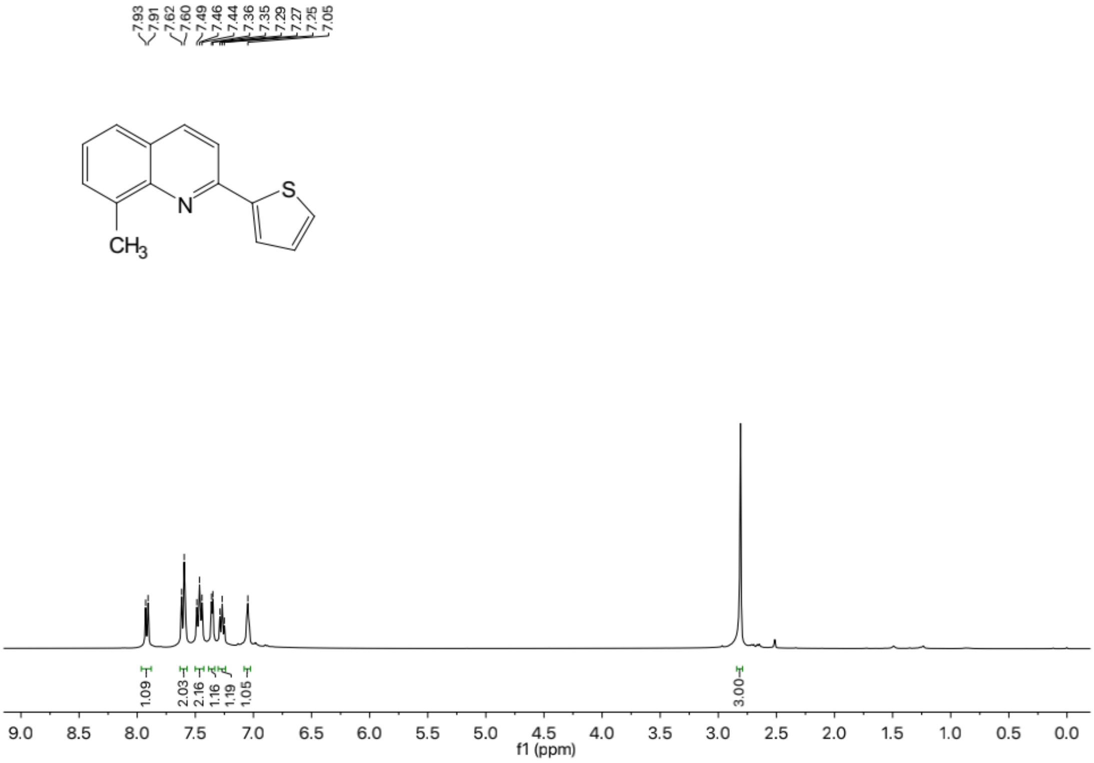

^{13}C NMR spectrum of 8-methyl-2-(thiophen-2-yl)quinoline（3bl）

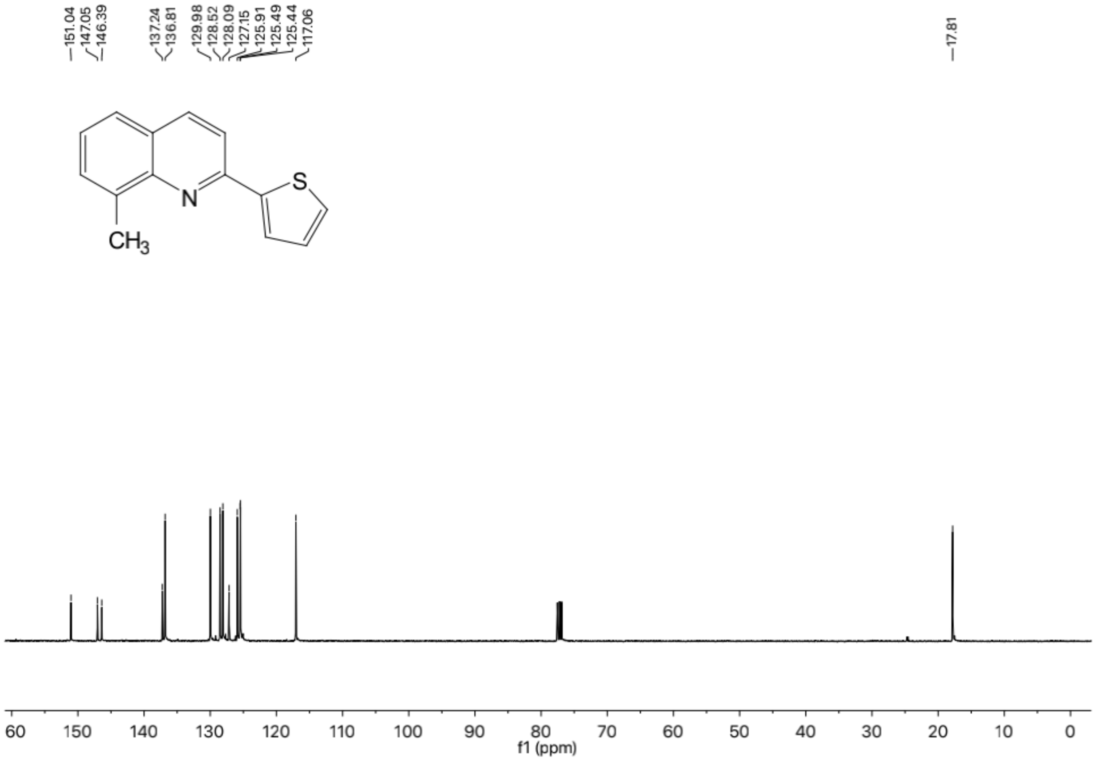

^{1}H NMR spectrum of 6-chloro-2-(thiophen-2-yl)quinoline（3cl）

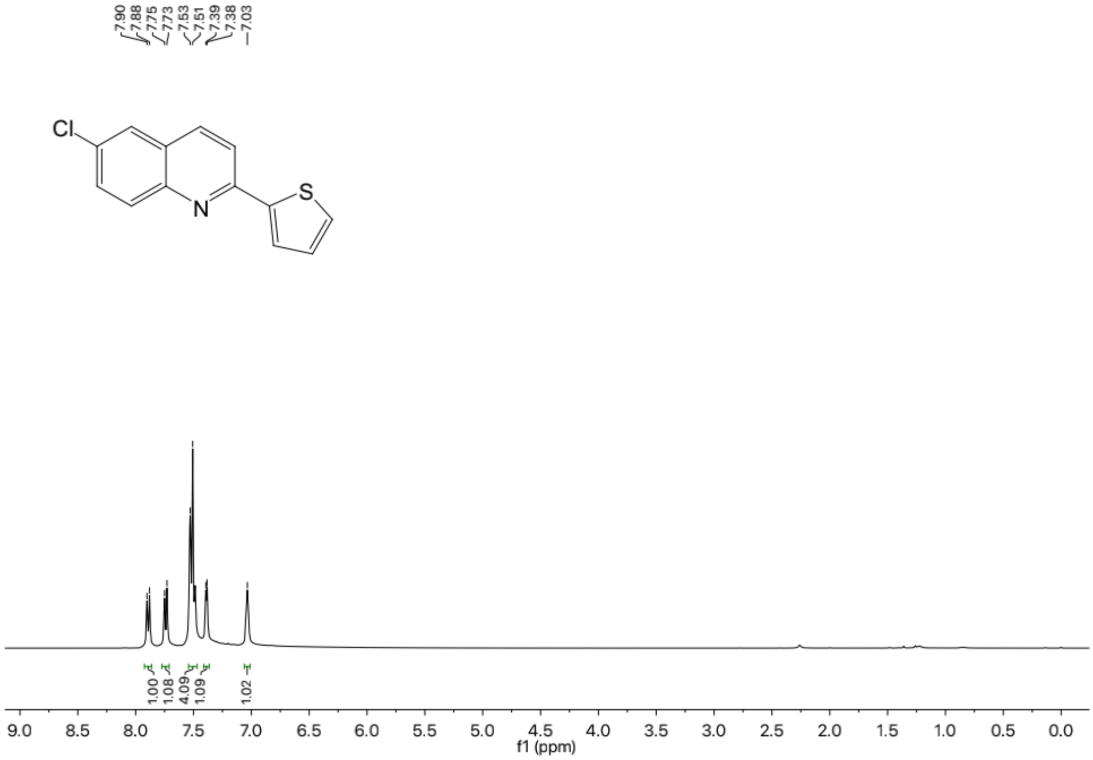

^{13}C NMR spectrum of 6-chloro-2-(thiophen-2-yl)quinoline（3cl）

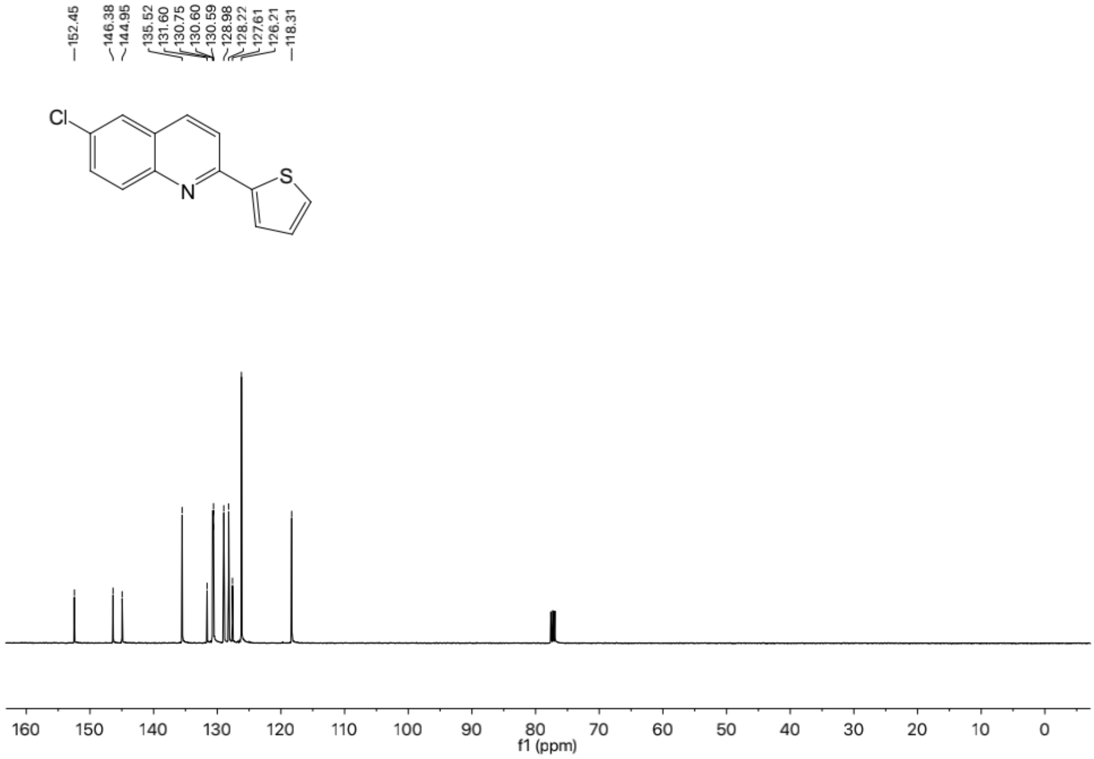

^{1}H NMR spectrum of 2-(pyridin-3-yl)quinoline（3am）

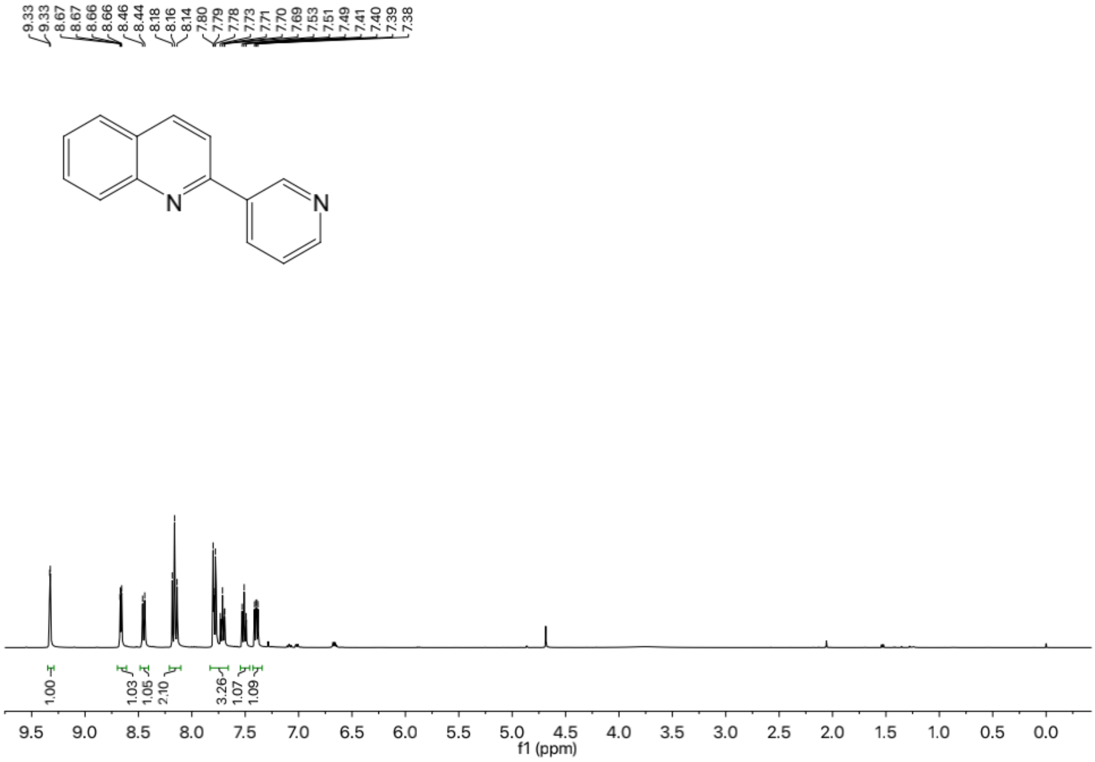

^{13}C NMR spectrum of 2-(pyridin-3-yl)quinoline（3am）

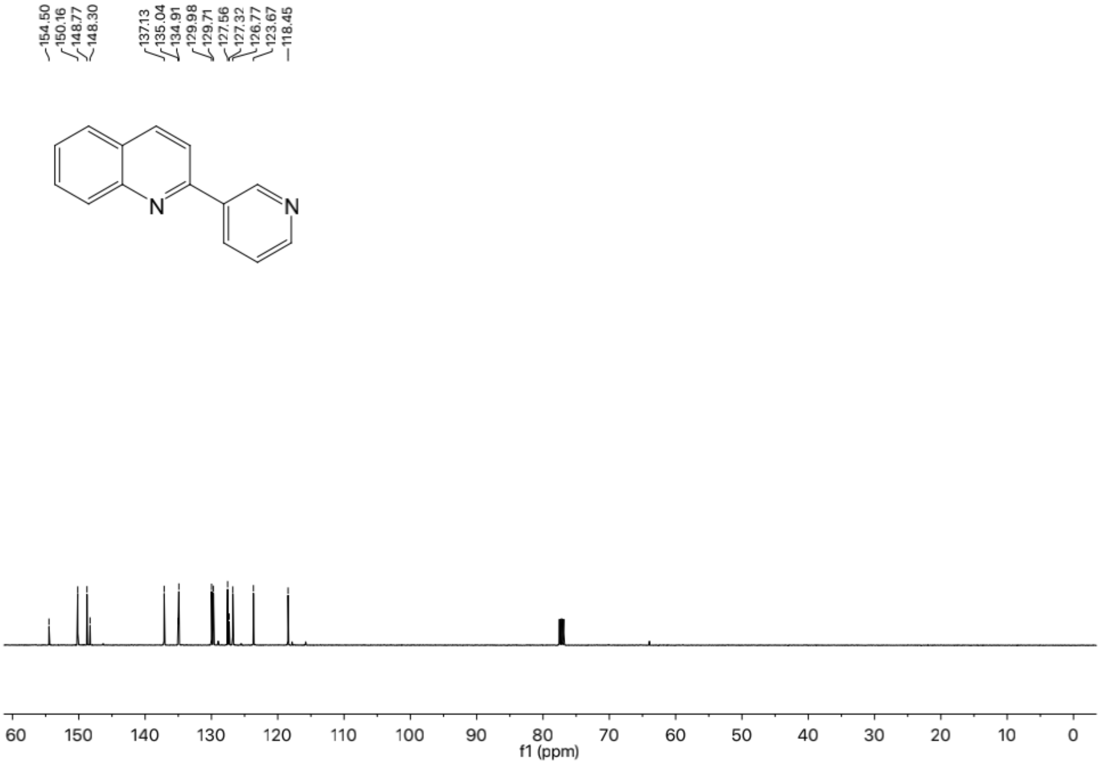

^{1}H NMR spectrum of 2-(6-methylpyridin-2-yl)quinoline（3an）

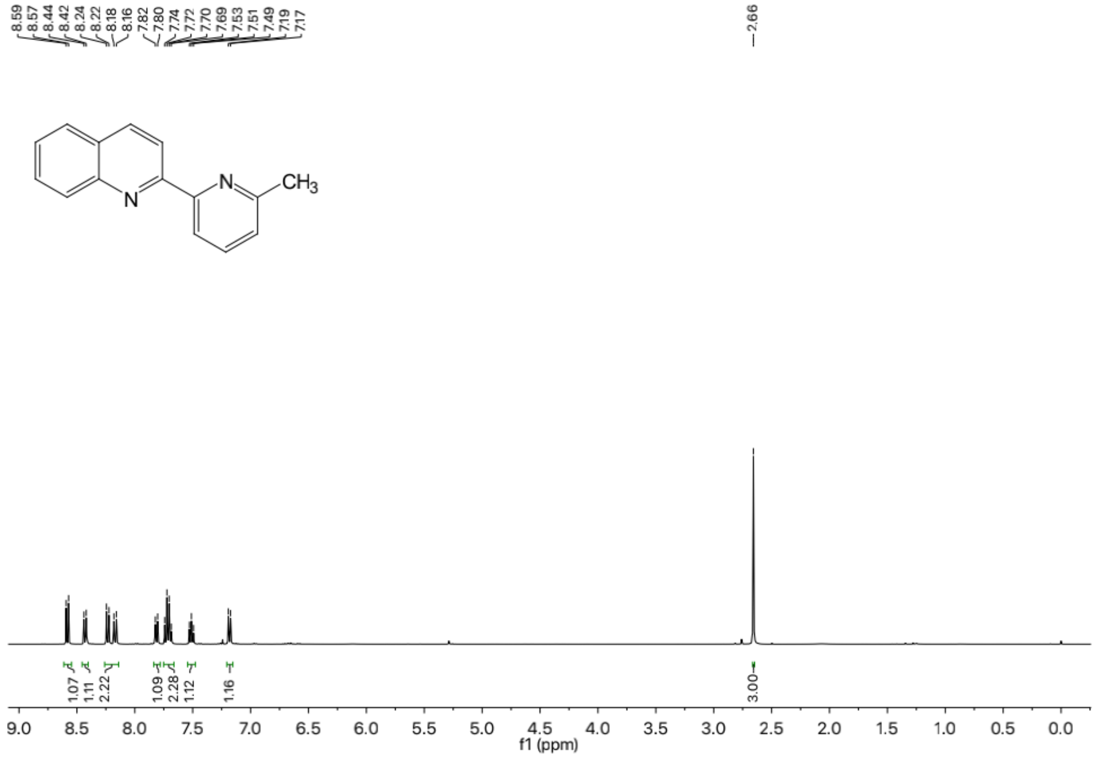

^{13}C NMR spectrum of 2-(6-methylpyridin-2-yl)quinoline（3an）

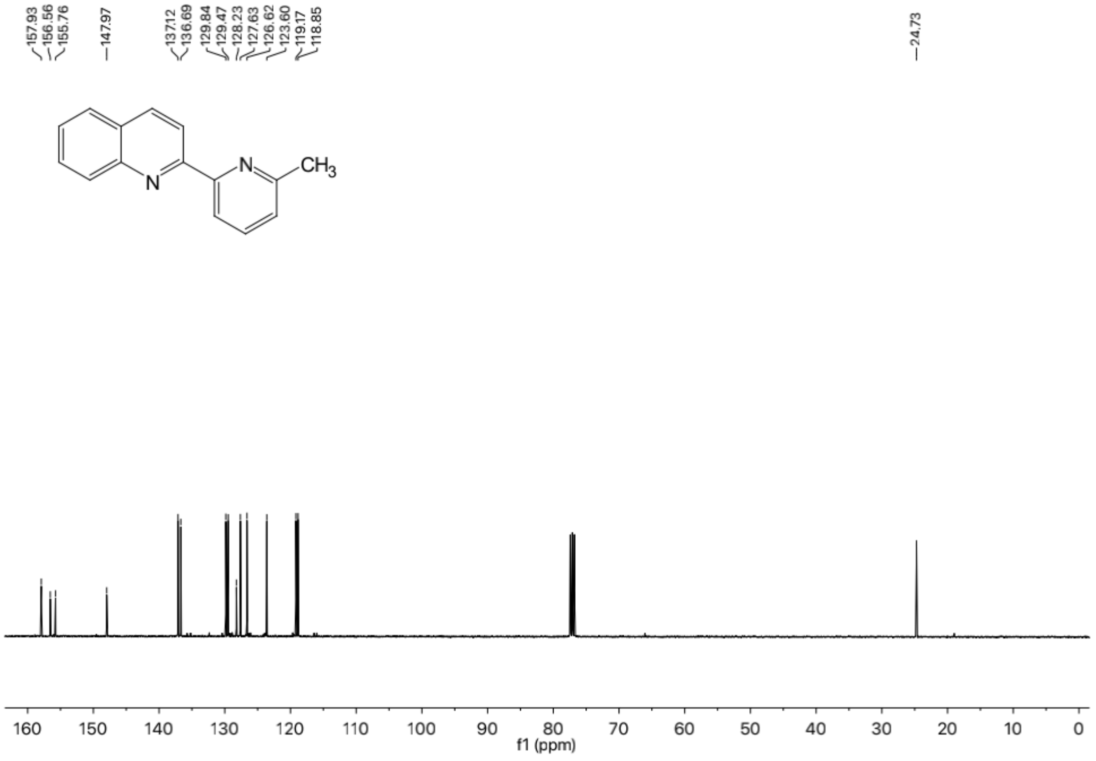

^{1}H NMR spectrum of 2-Cyclohexyl-quinoline (3ao)

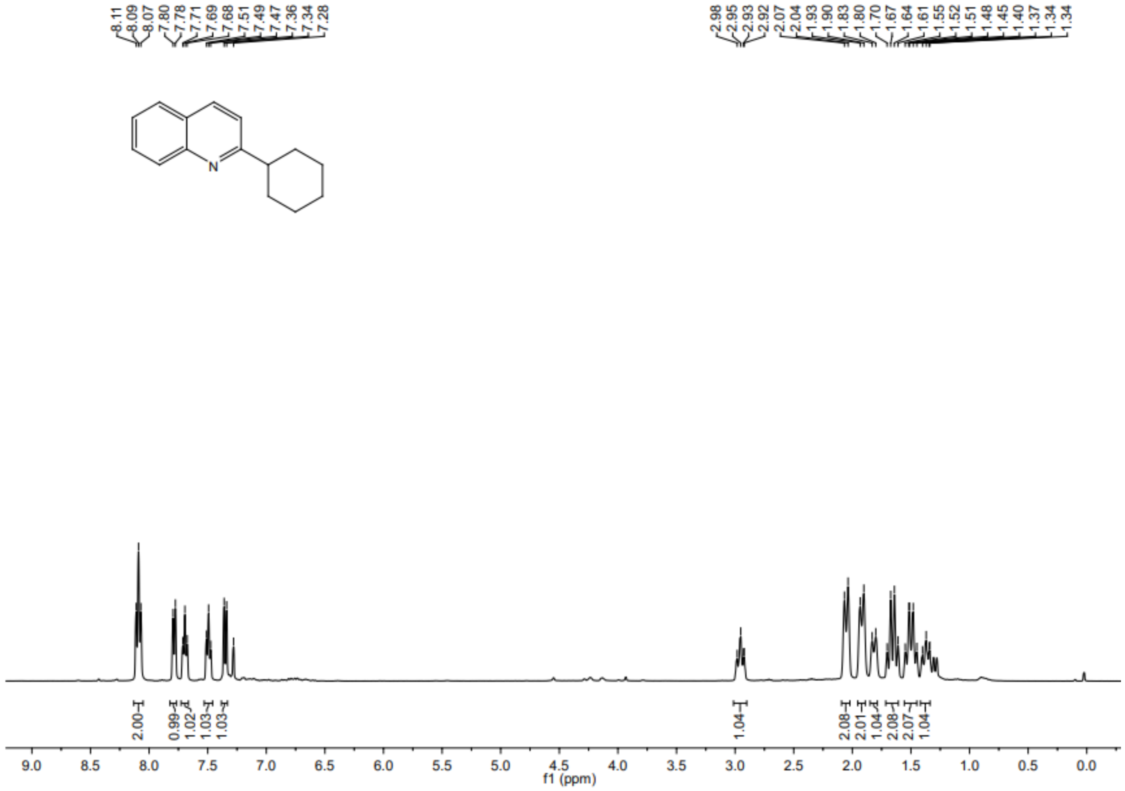

^{13}C NMR spectrum of 2-Cyclohexyl-quinoline (3ao)

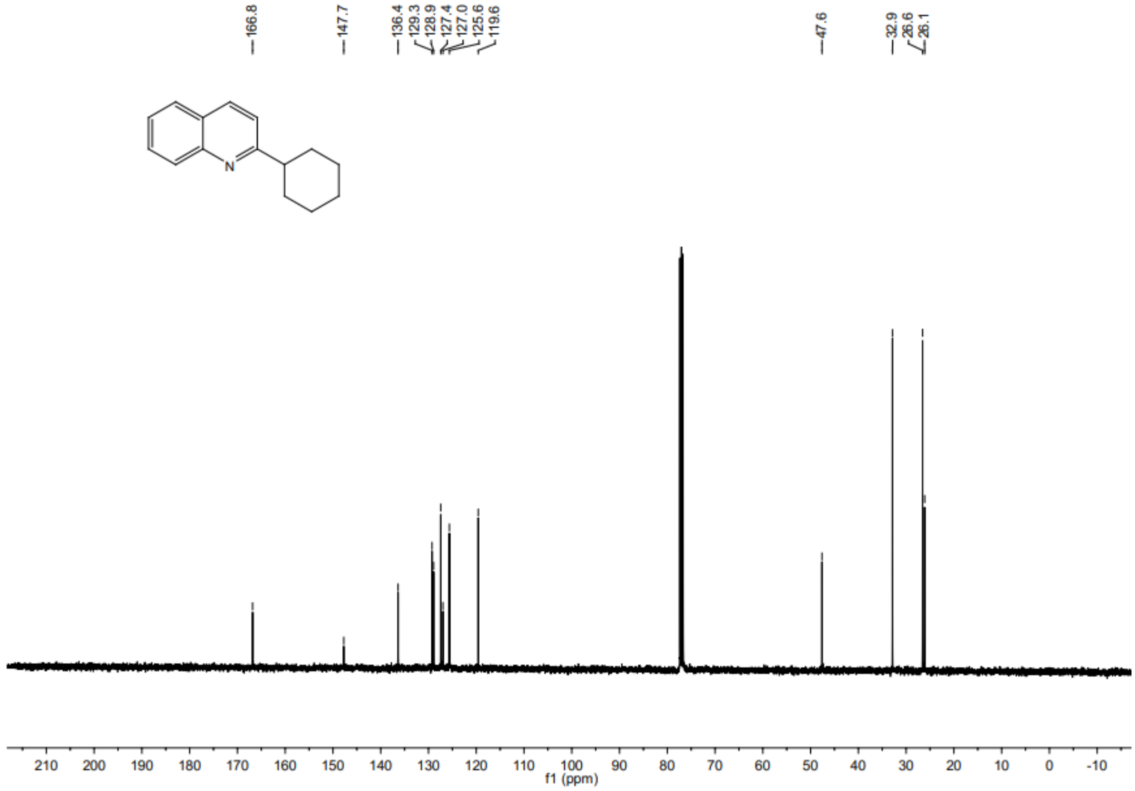

^{1}H NMR spectrum of 3-propylquinoline (3ap)

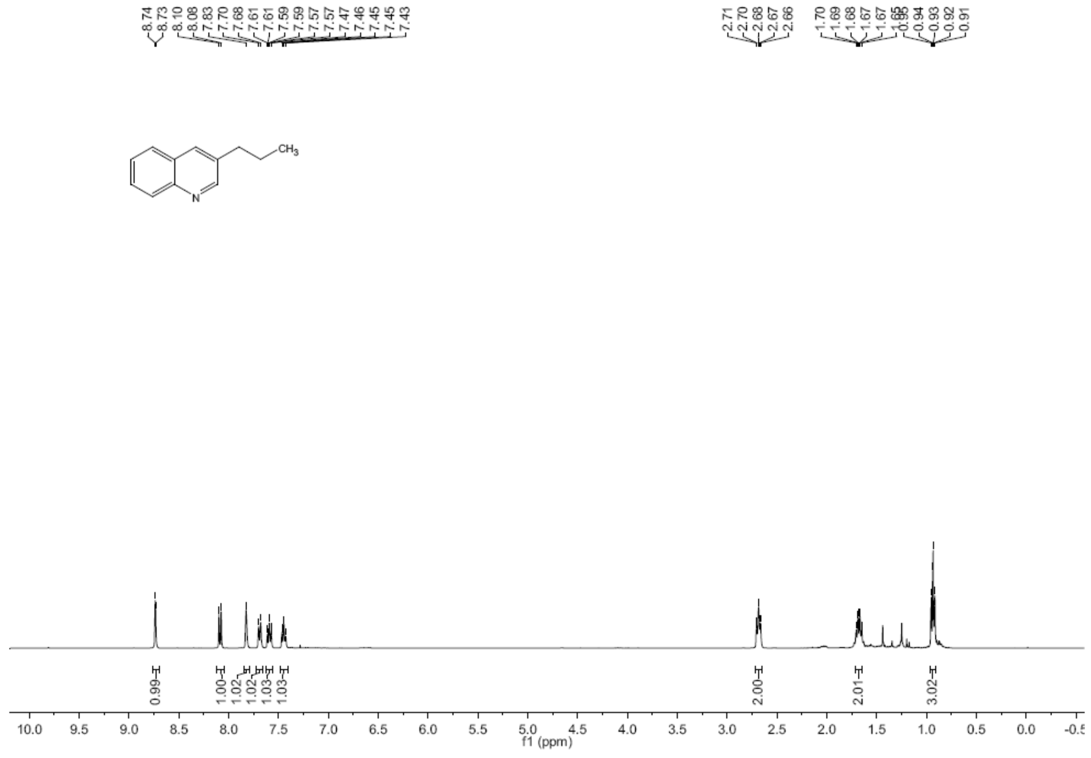

^{13}C NMR spectrum of 3-propylquinoline (3ap)

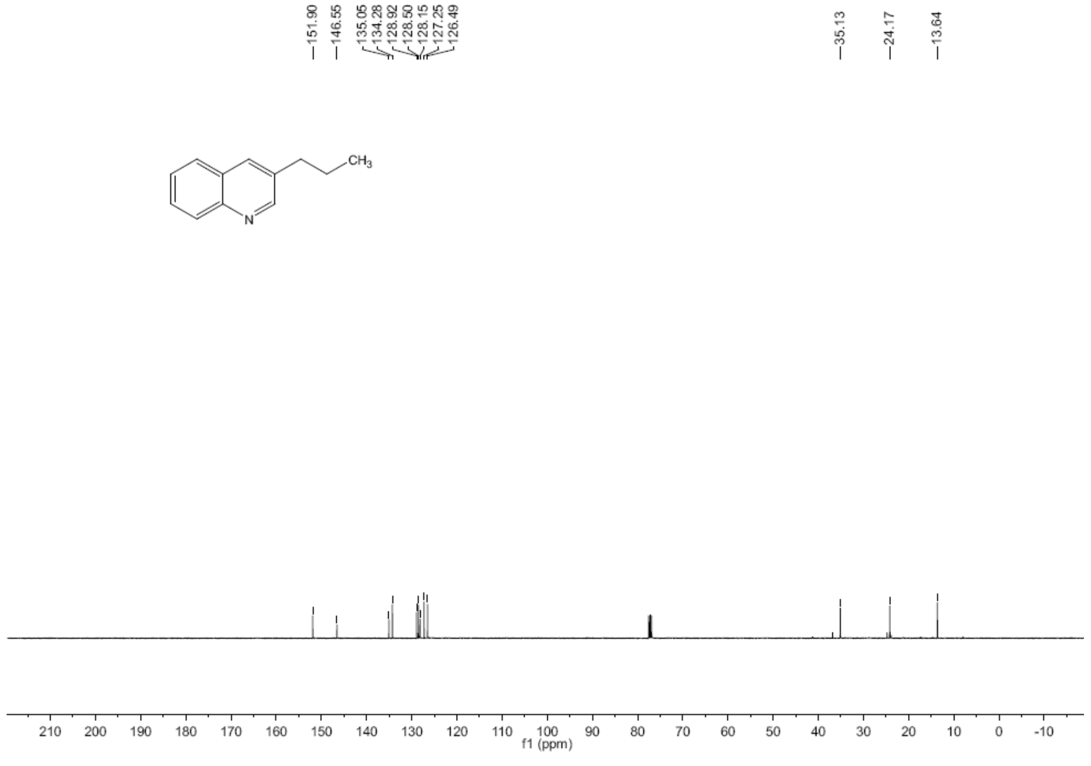

7. HRMS of new compounds

The HRMS of compound 3bb

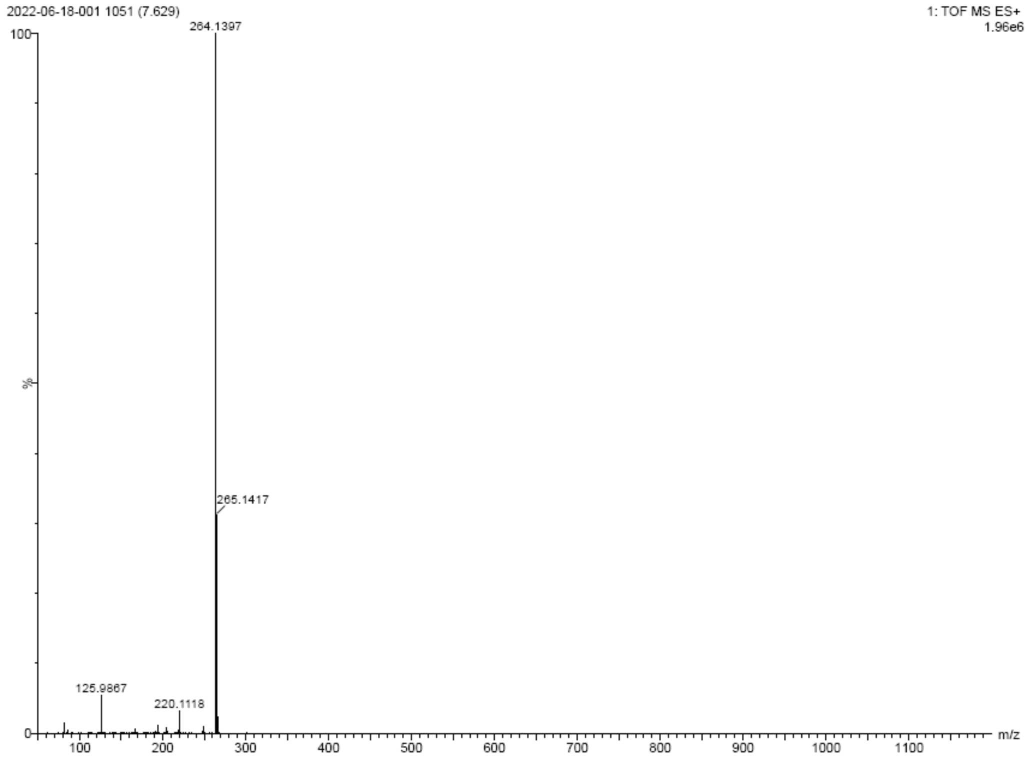

The HRMS of compound 3bc

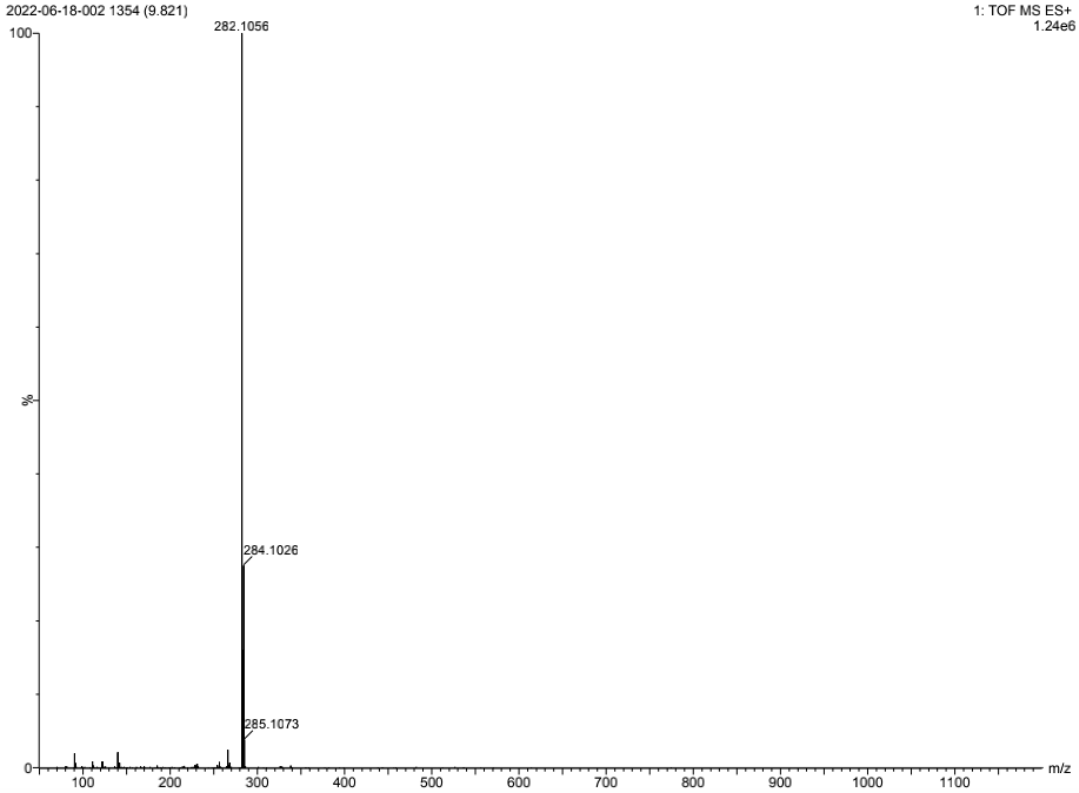

The HRMS of compound 3cc

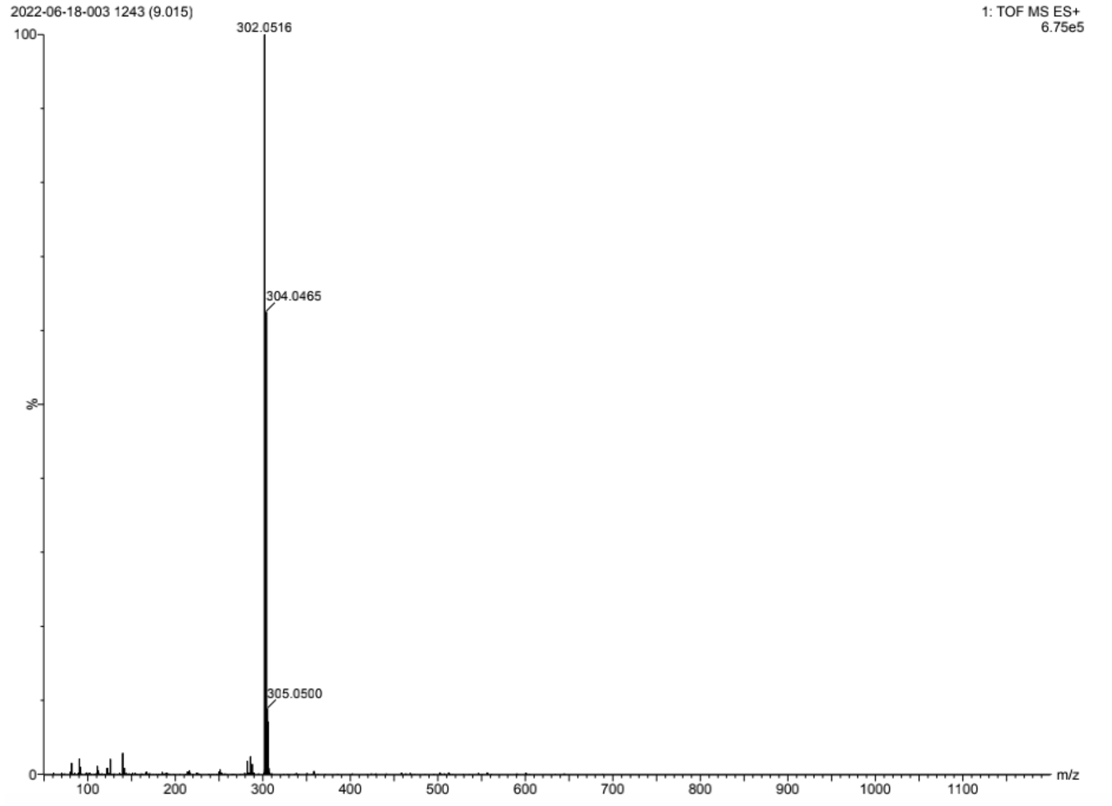

The HRMS of compound 3ad

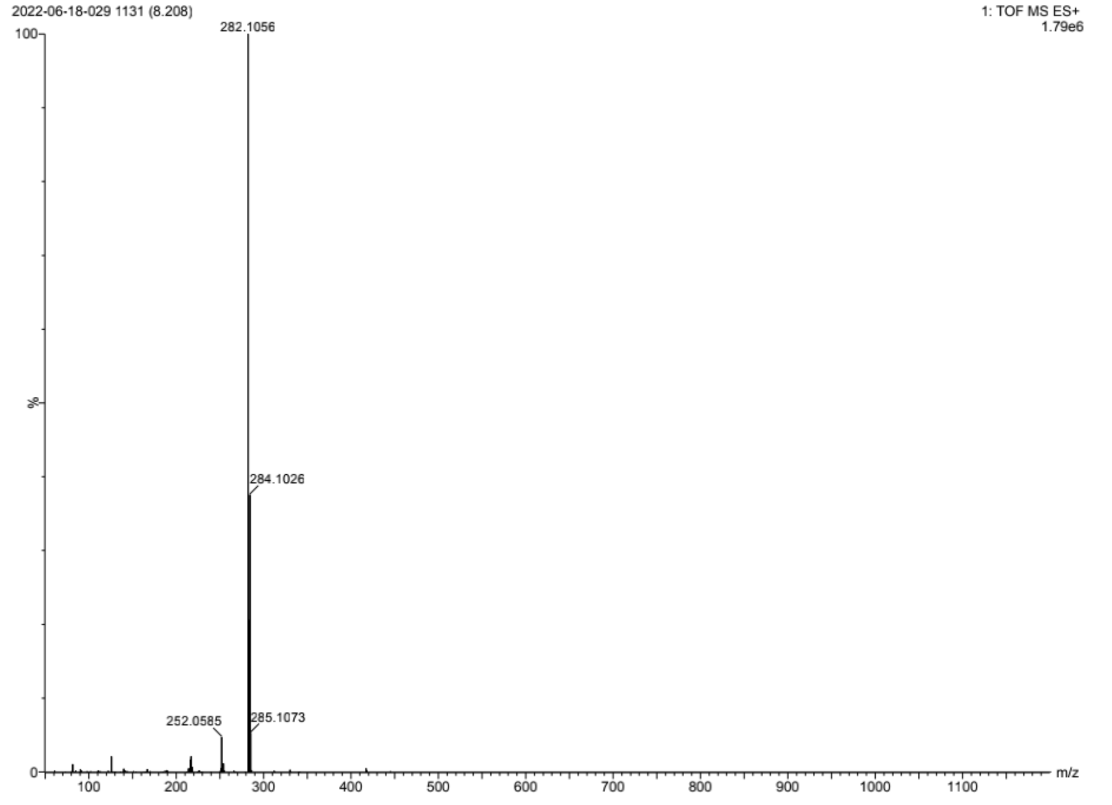

The HRMS of compound 3bd

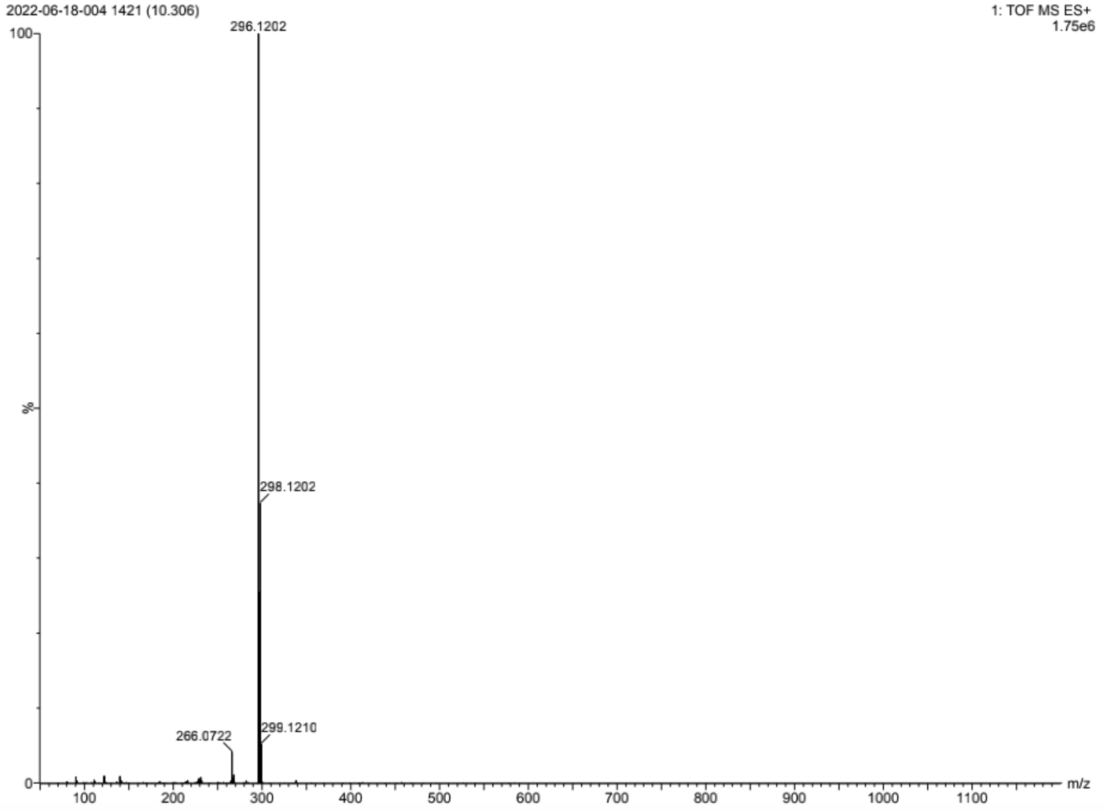

The HRMS of compound 3cd

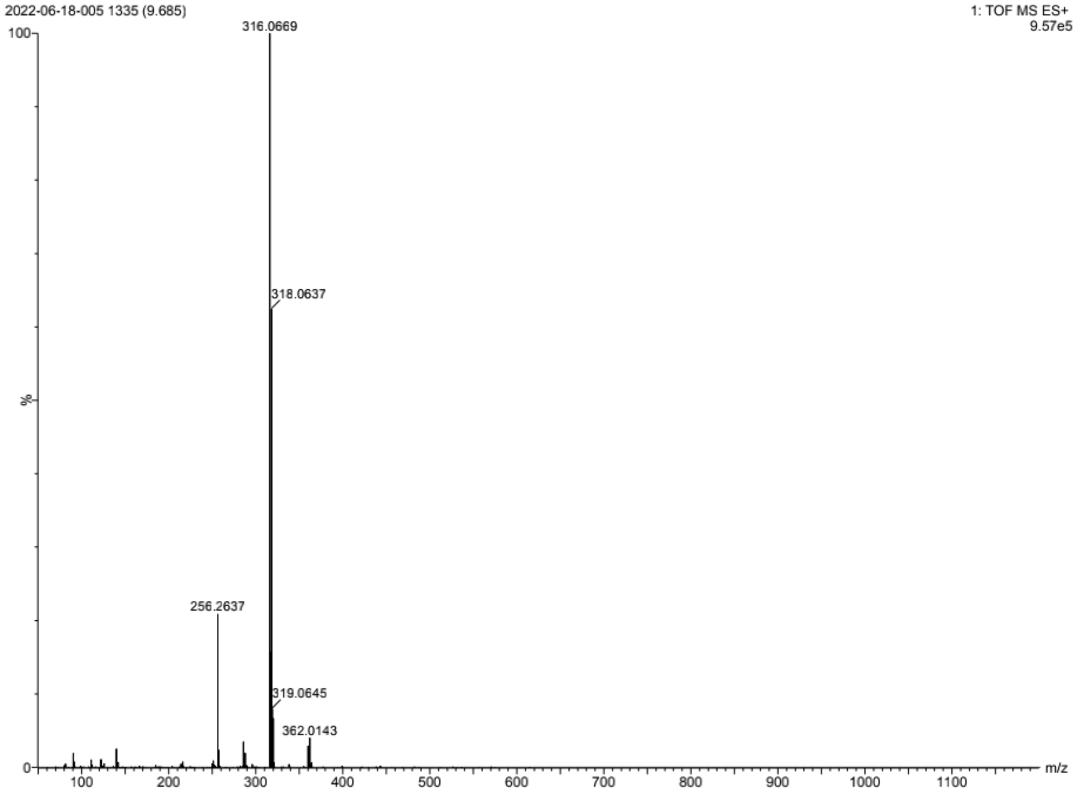

The HRMS of compound 3dd

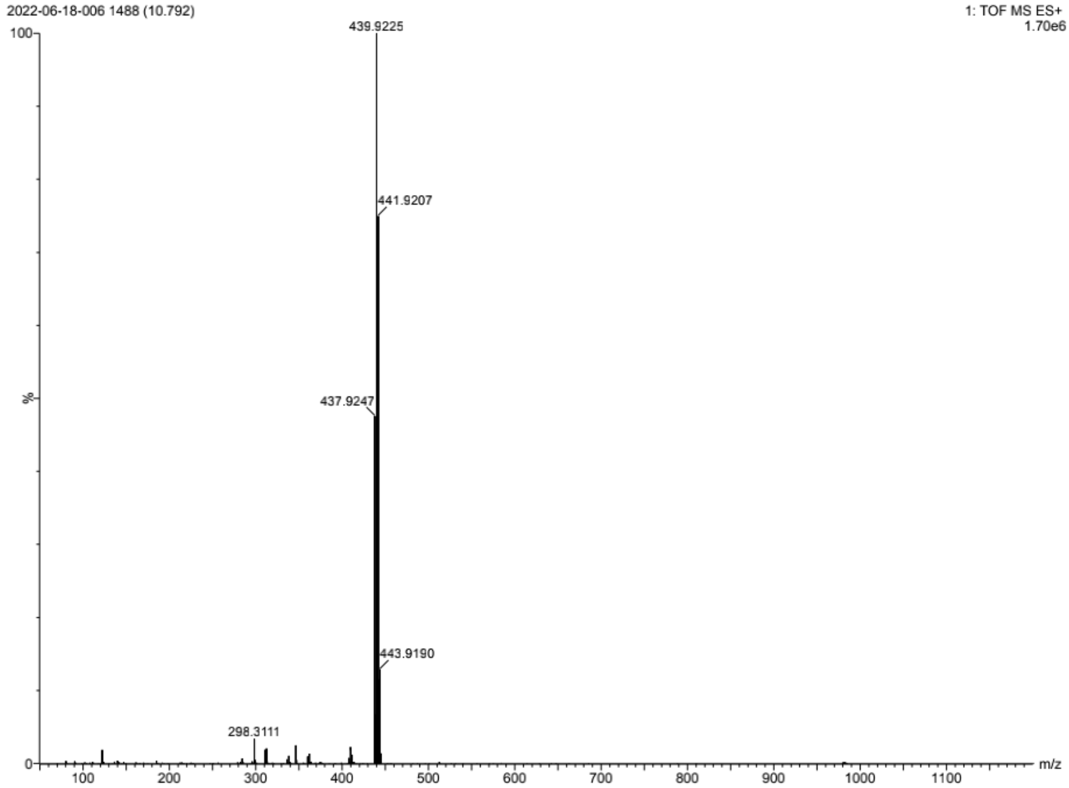

The HRMS of compound 3ae

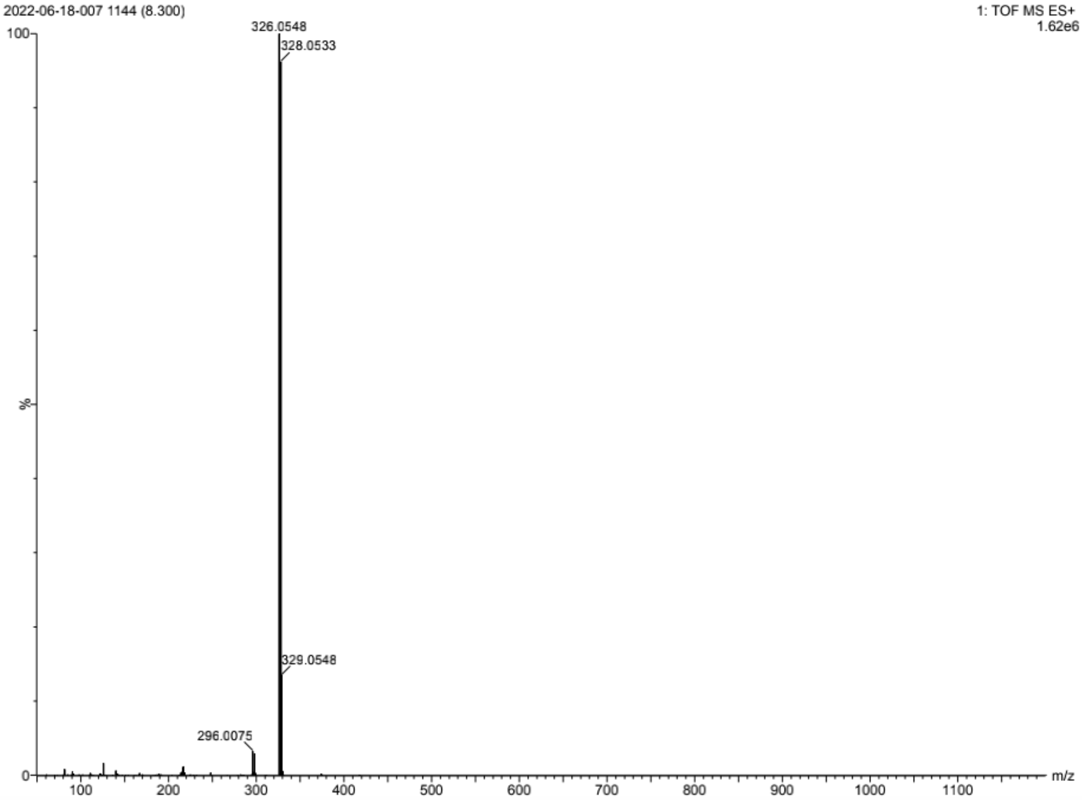

The HRMS of compound 3be

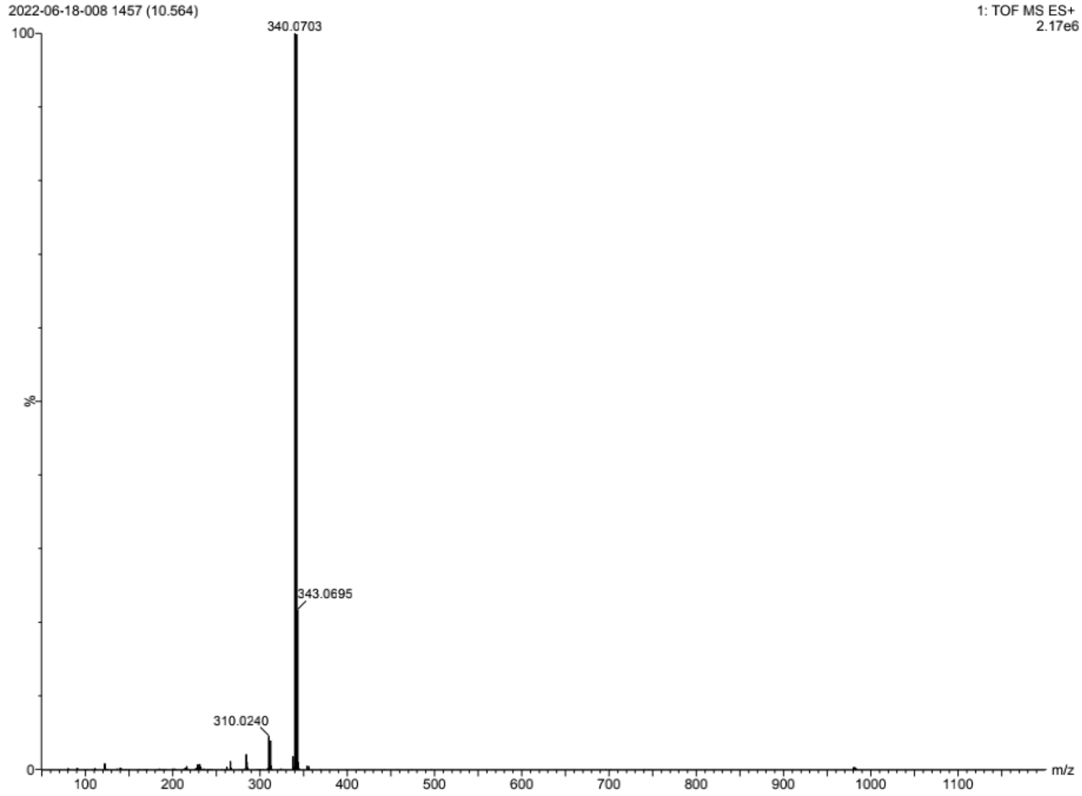

The HRMS of compound 3ce

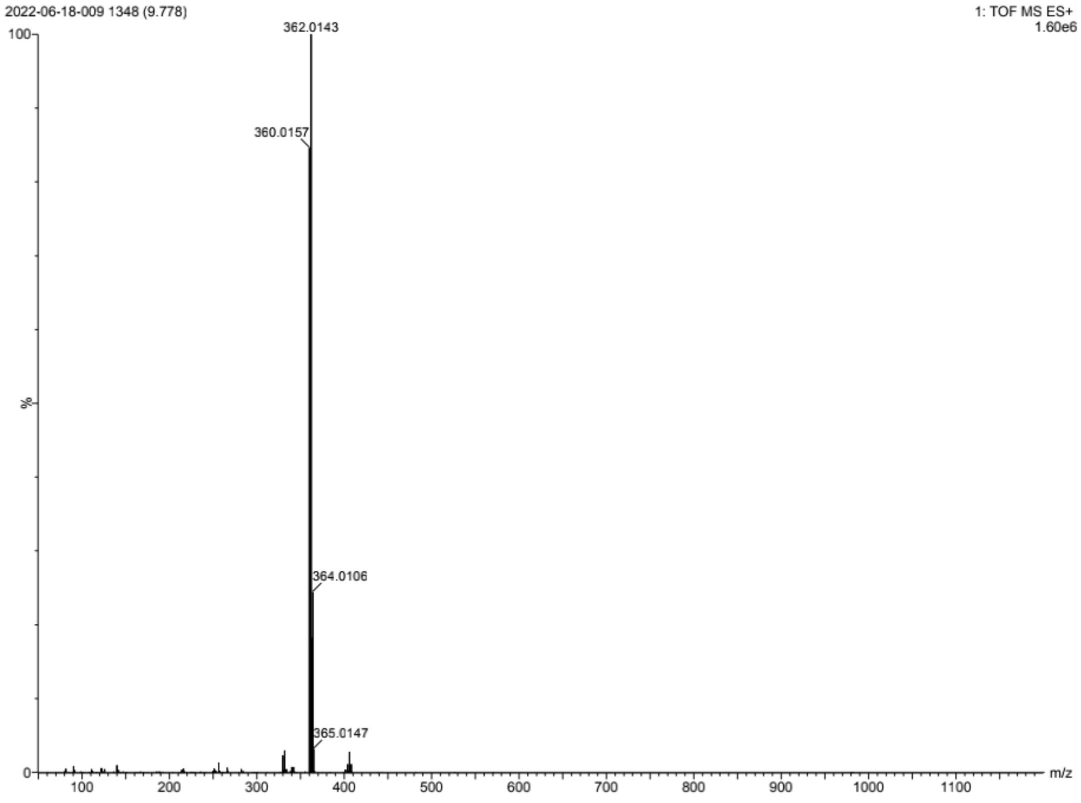

The HRMS of compound 3de

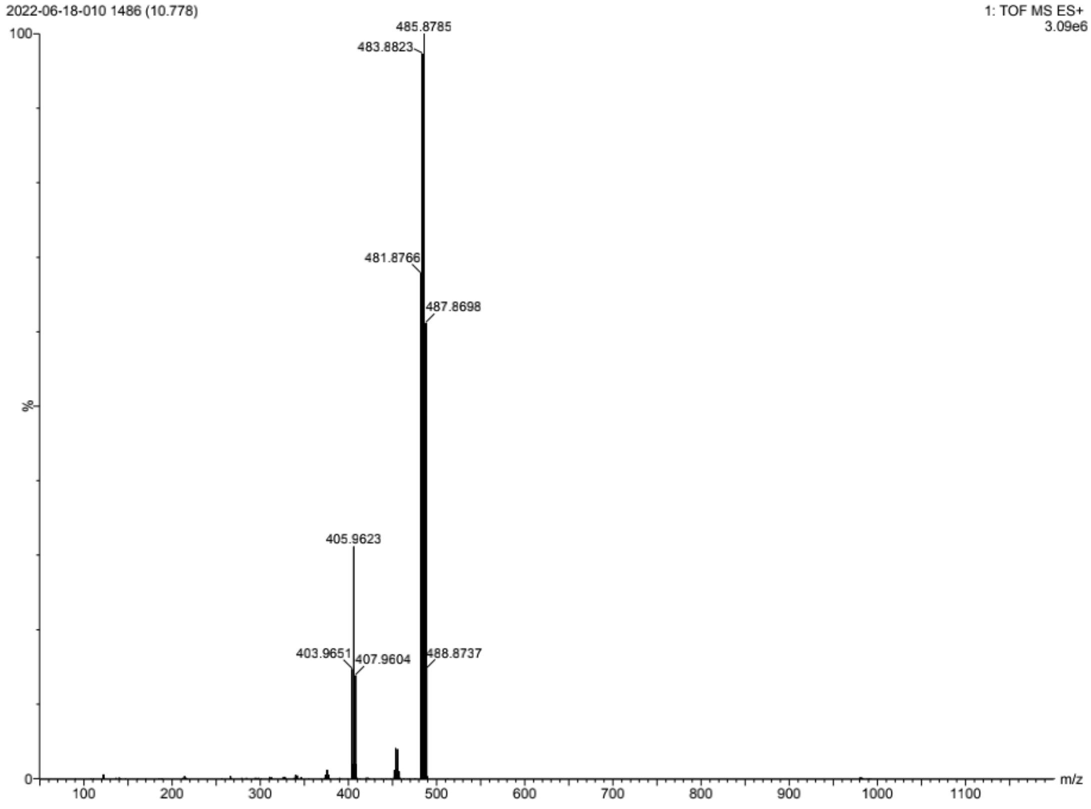

The HRMS of compound 3af

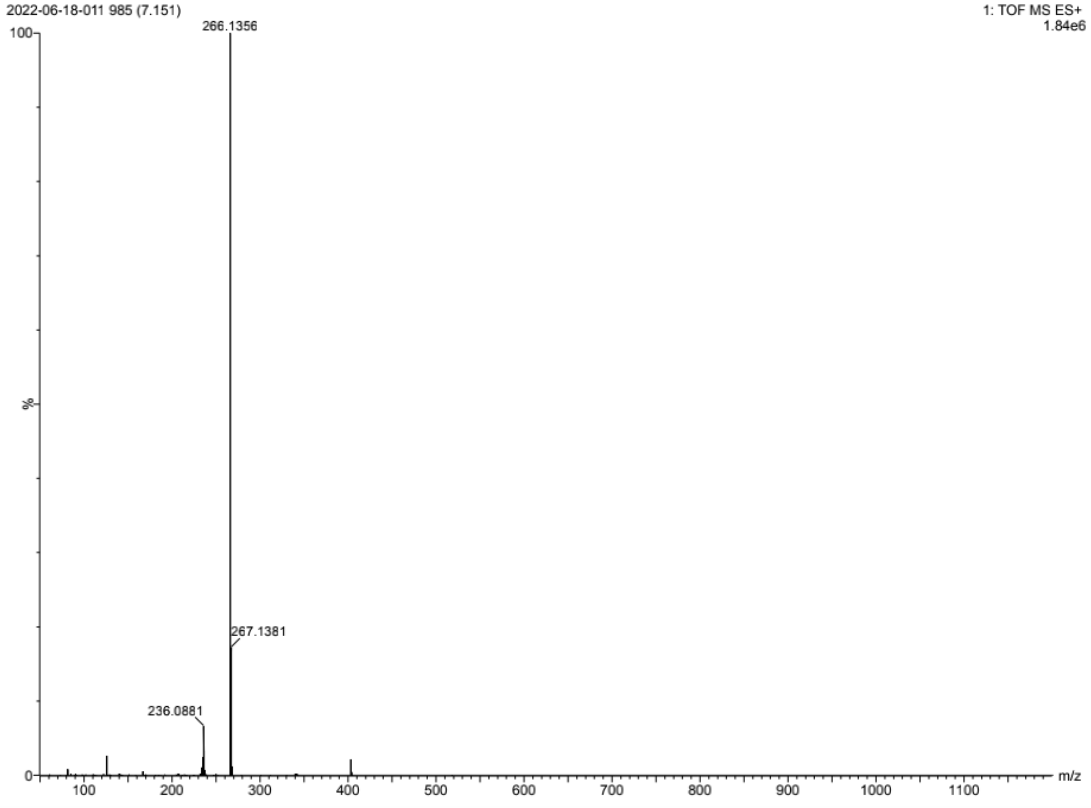

The HRMS of compound 3bf

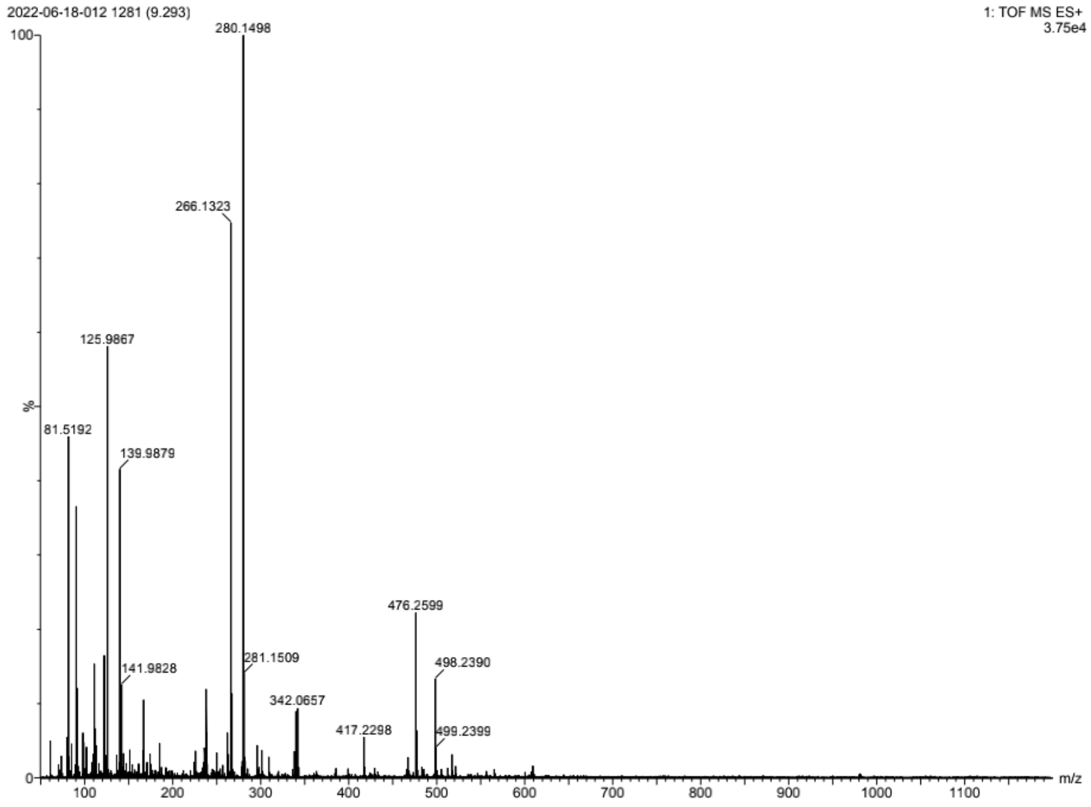

The HRMS of compound 3cf

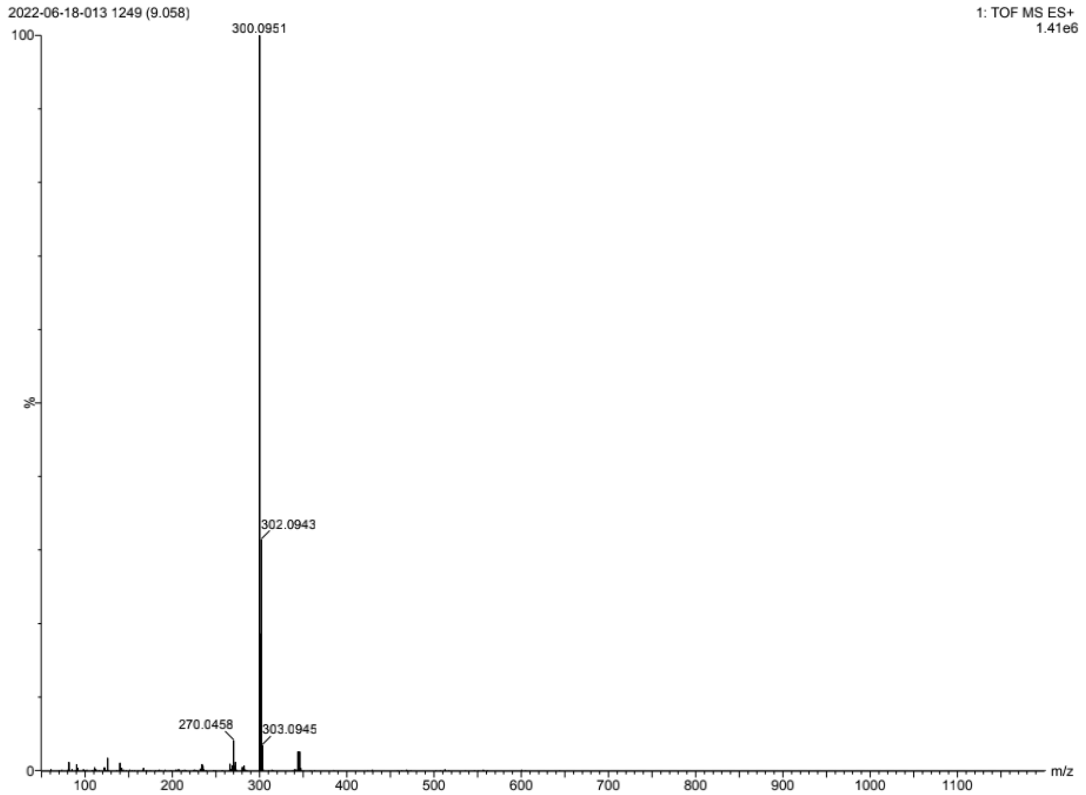

The HRMS of compound 3df

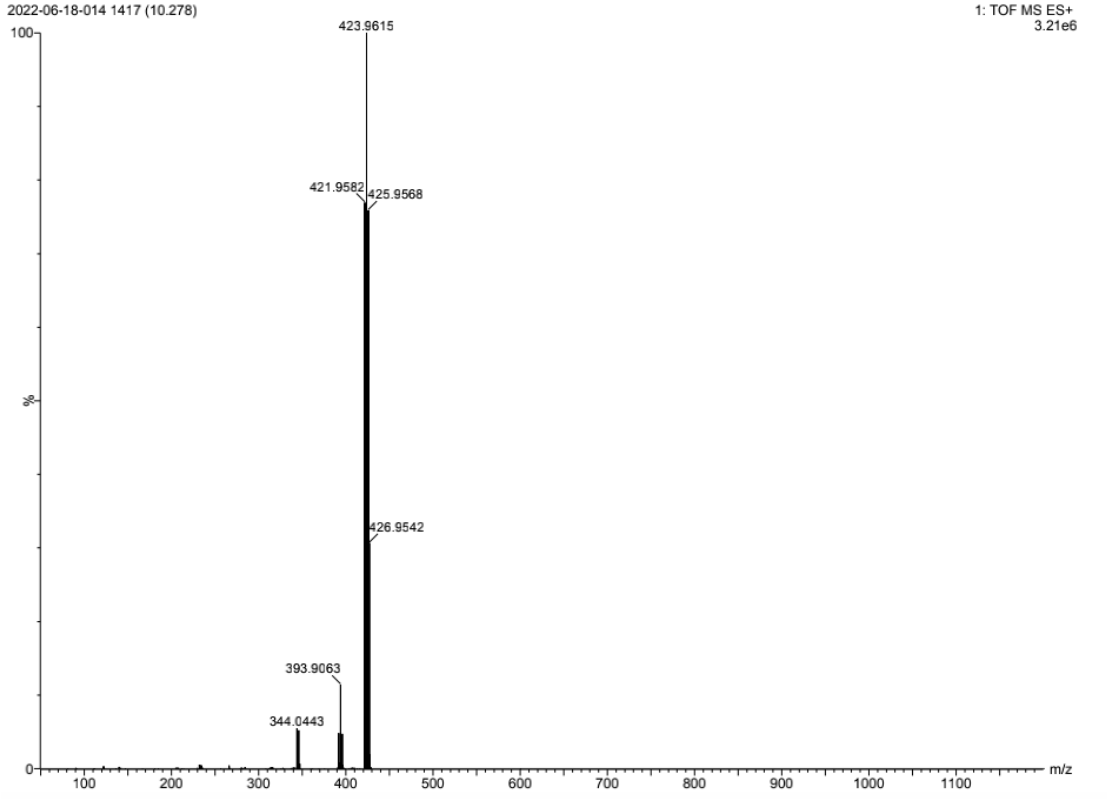

The HRMS of compound 3ag

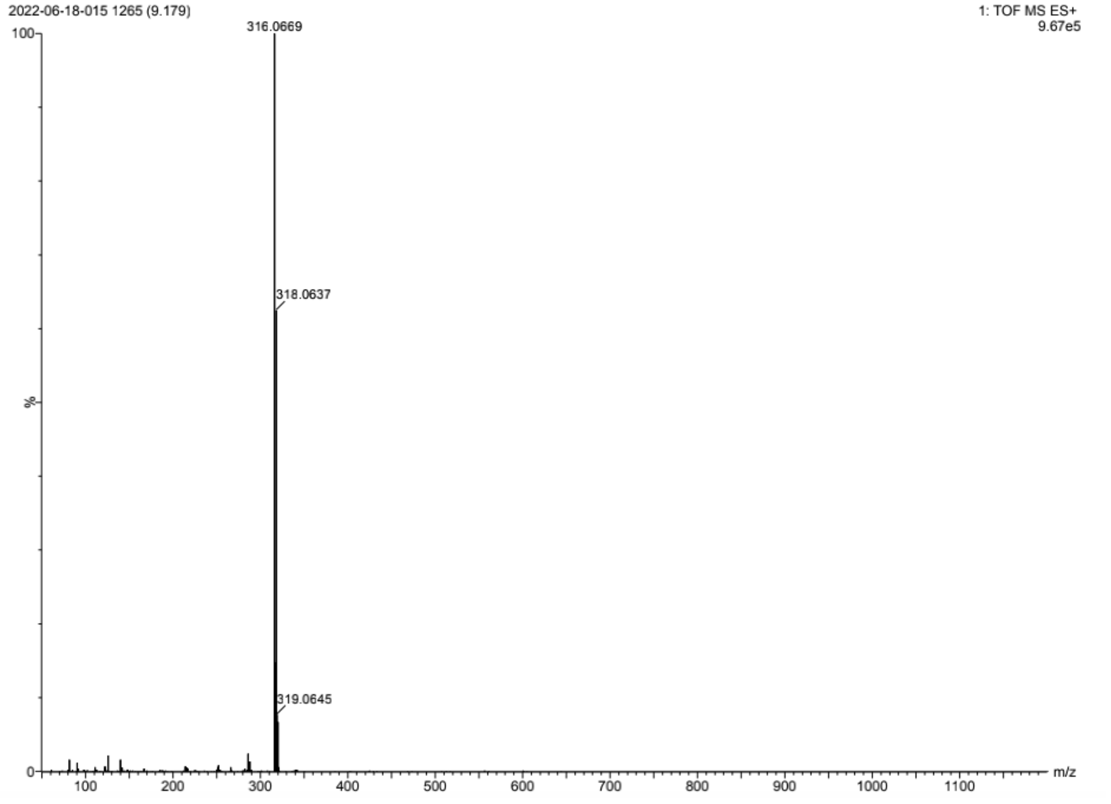

The HRMS of compound 3bg

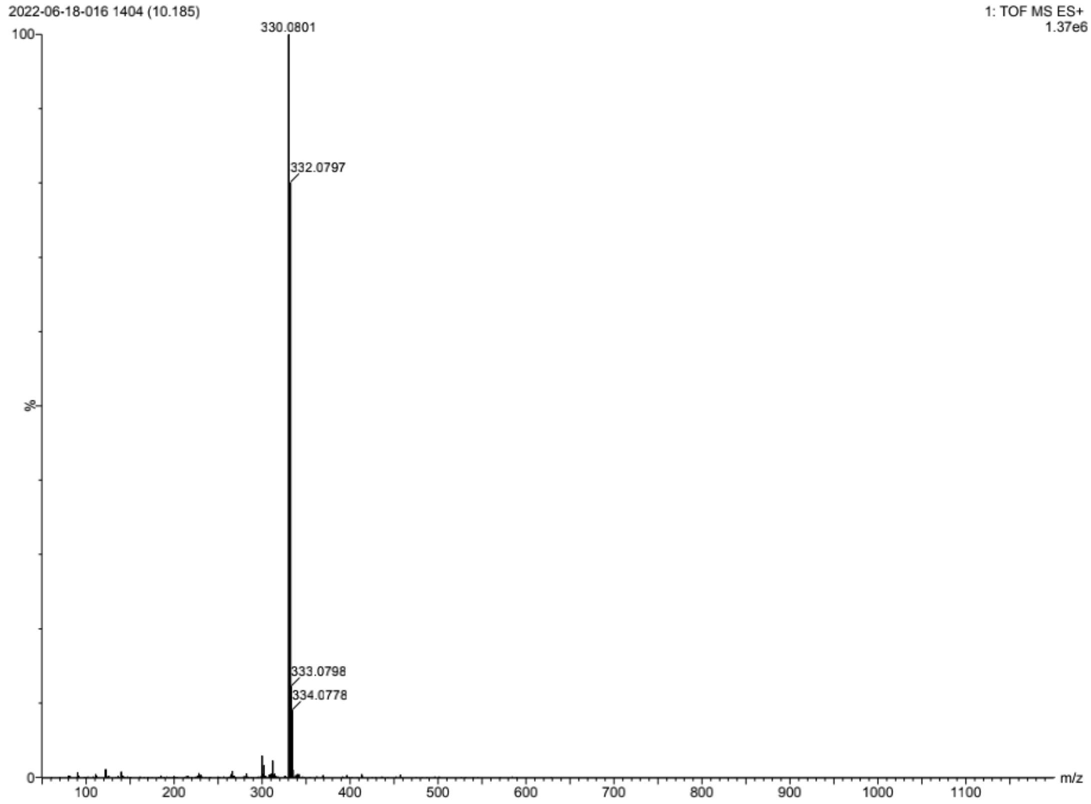

The HRMS of compound 3cg

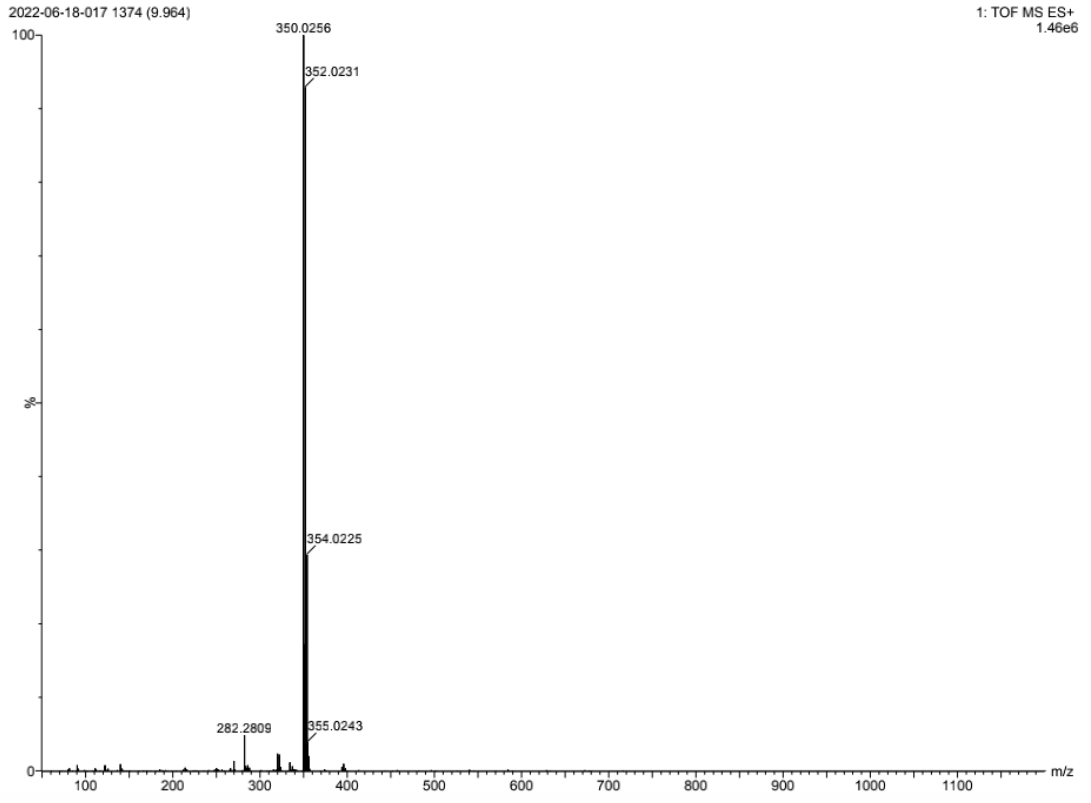

The HRMS of compound 3dg

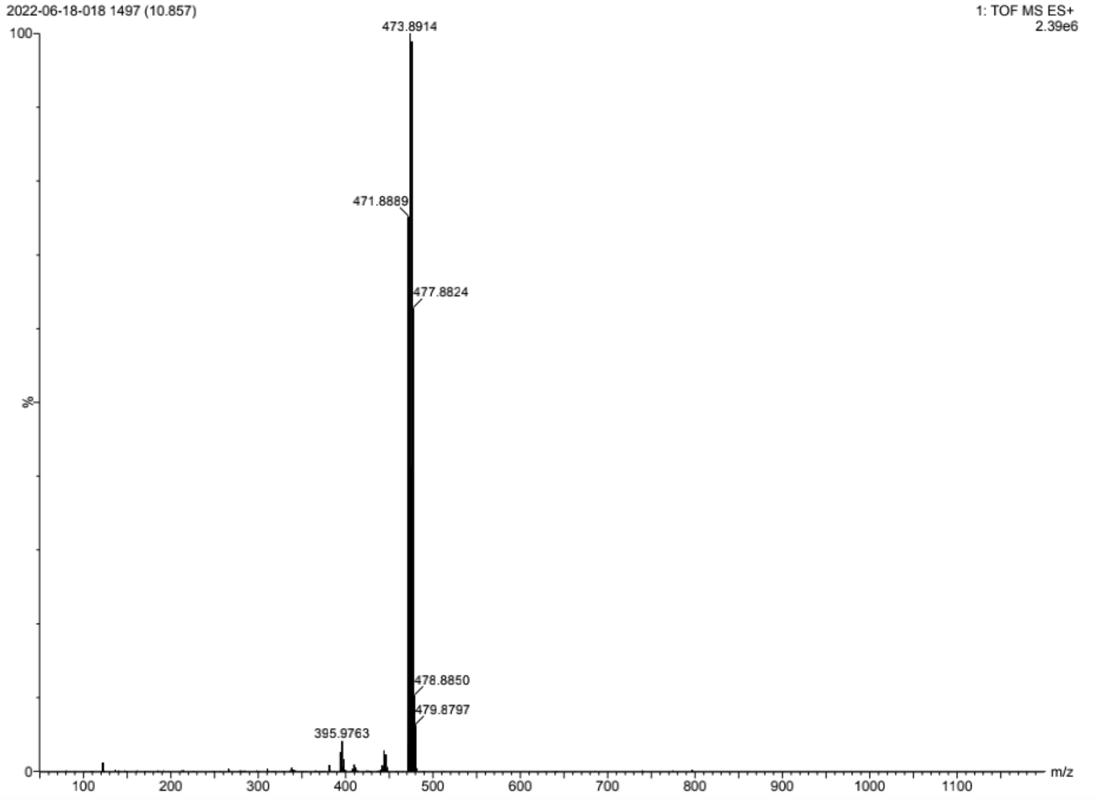

The HRMS of compound 3ah

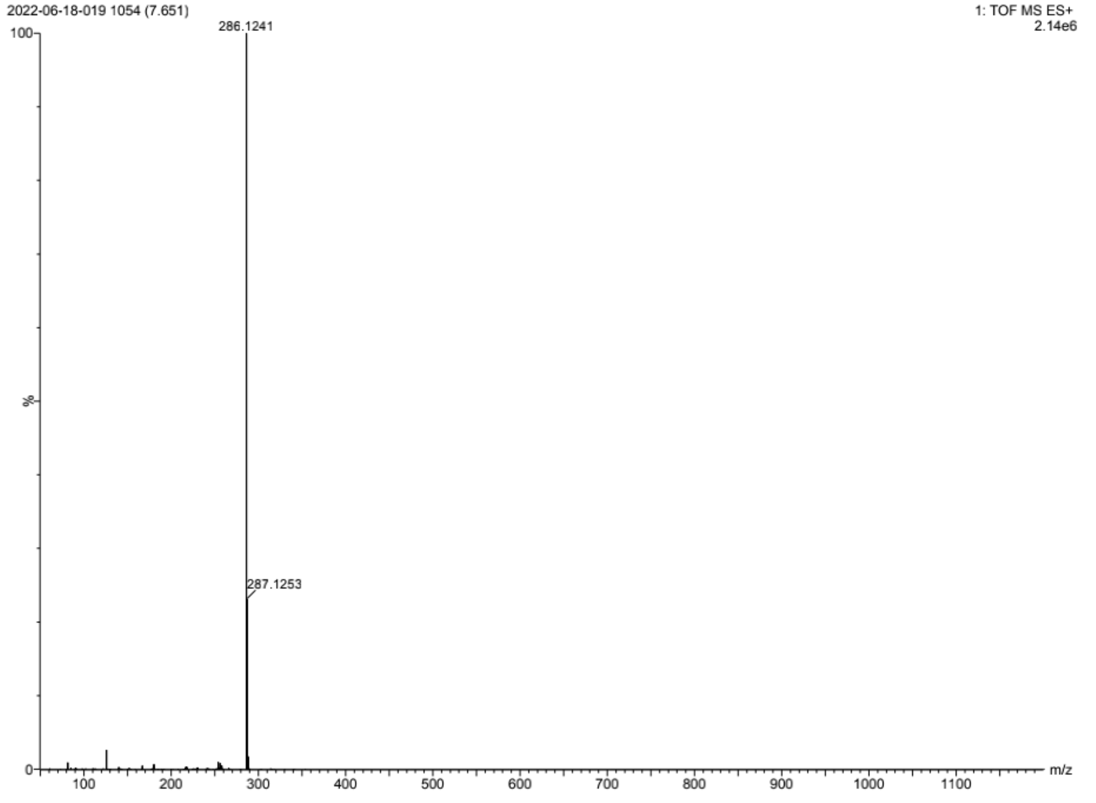

The HRMS of compound 3bcdi

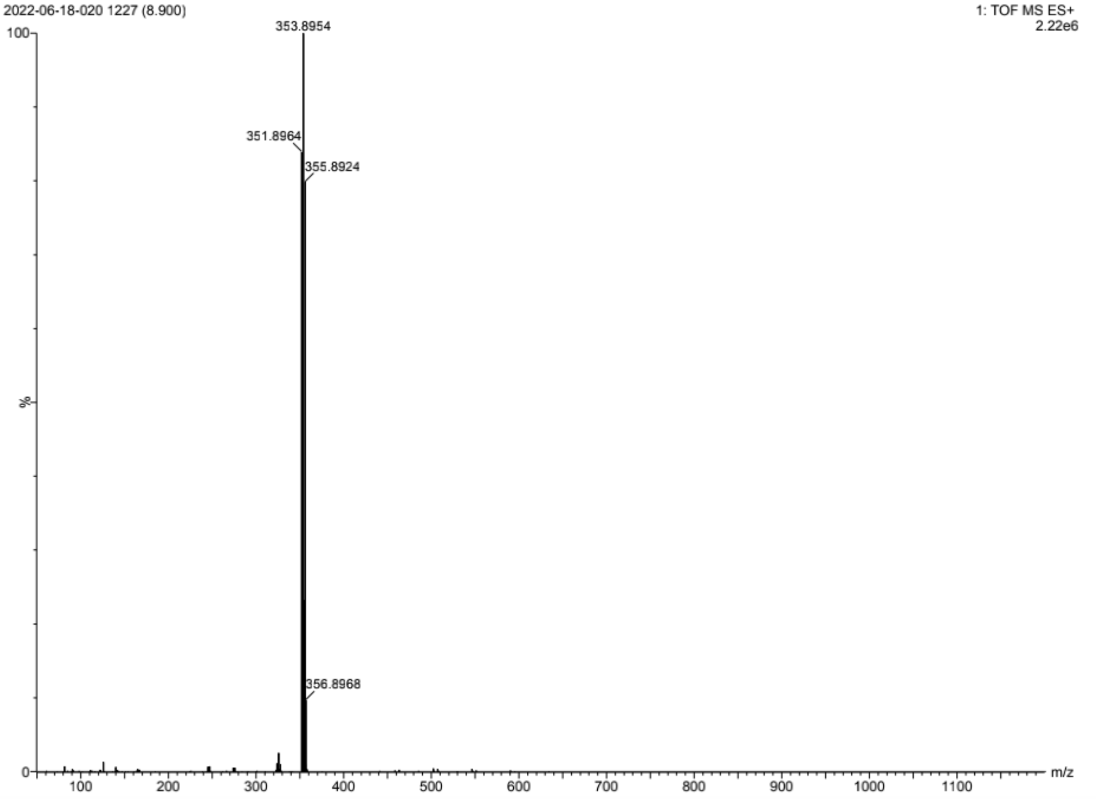

The HRMS of compound 3bj

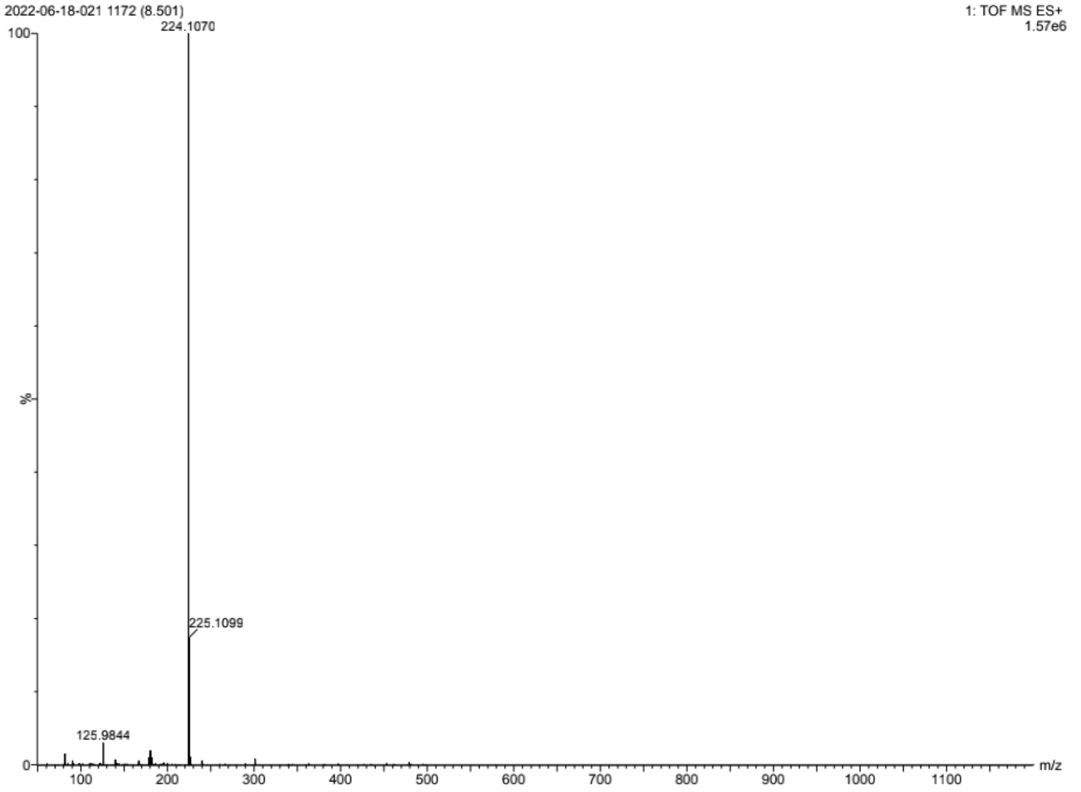

The HRMS of compound 3cj

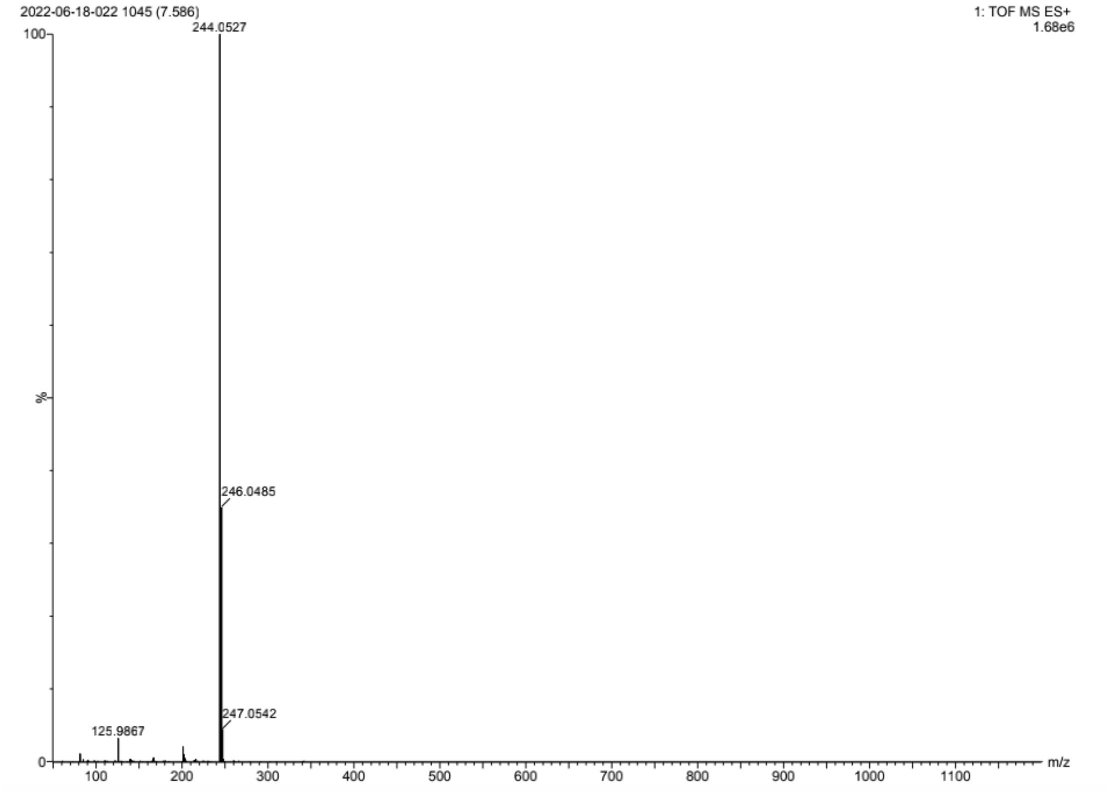

The HRMS of compound 3dj

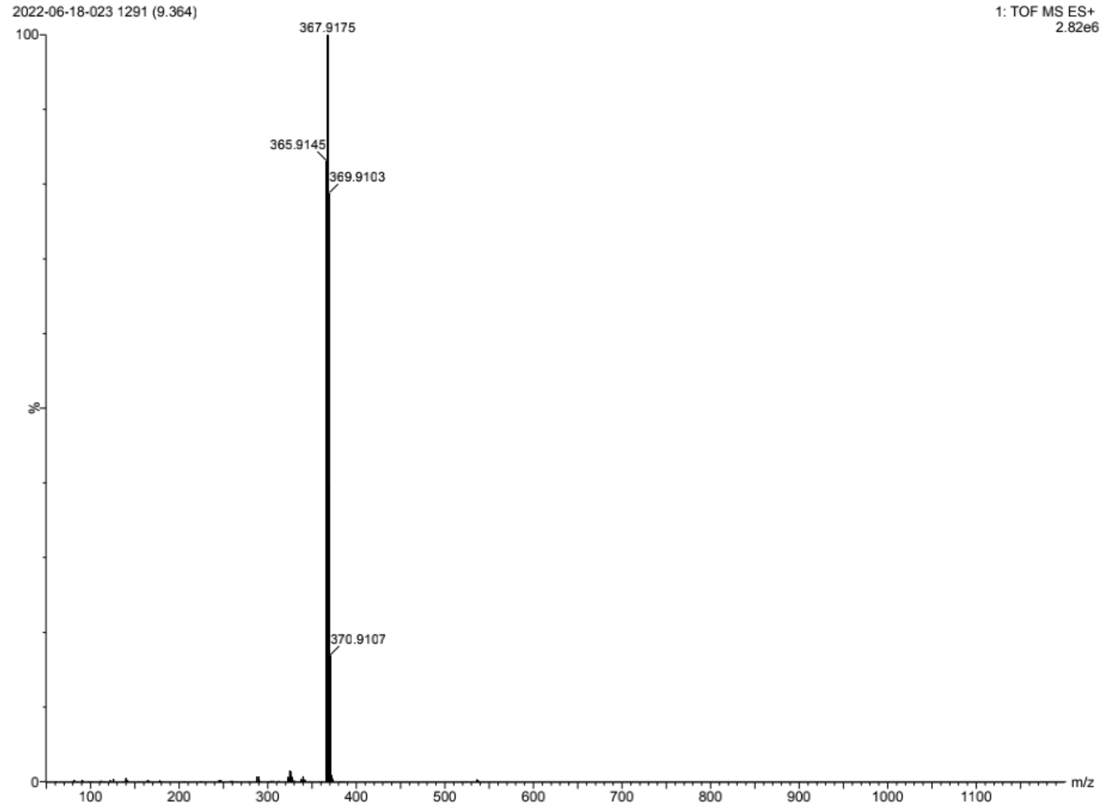

The HRMS of compound 3ak

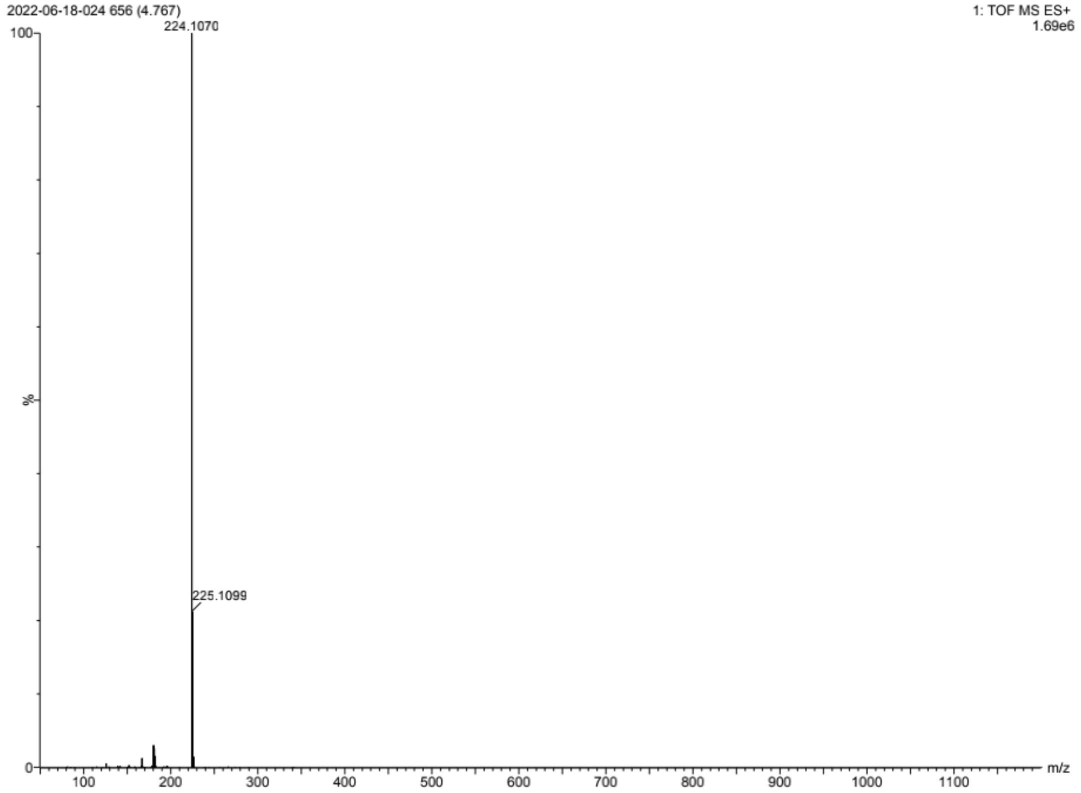

The HRMS of compound 3bj

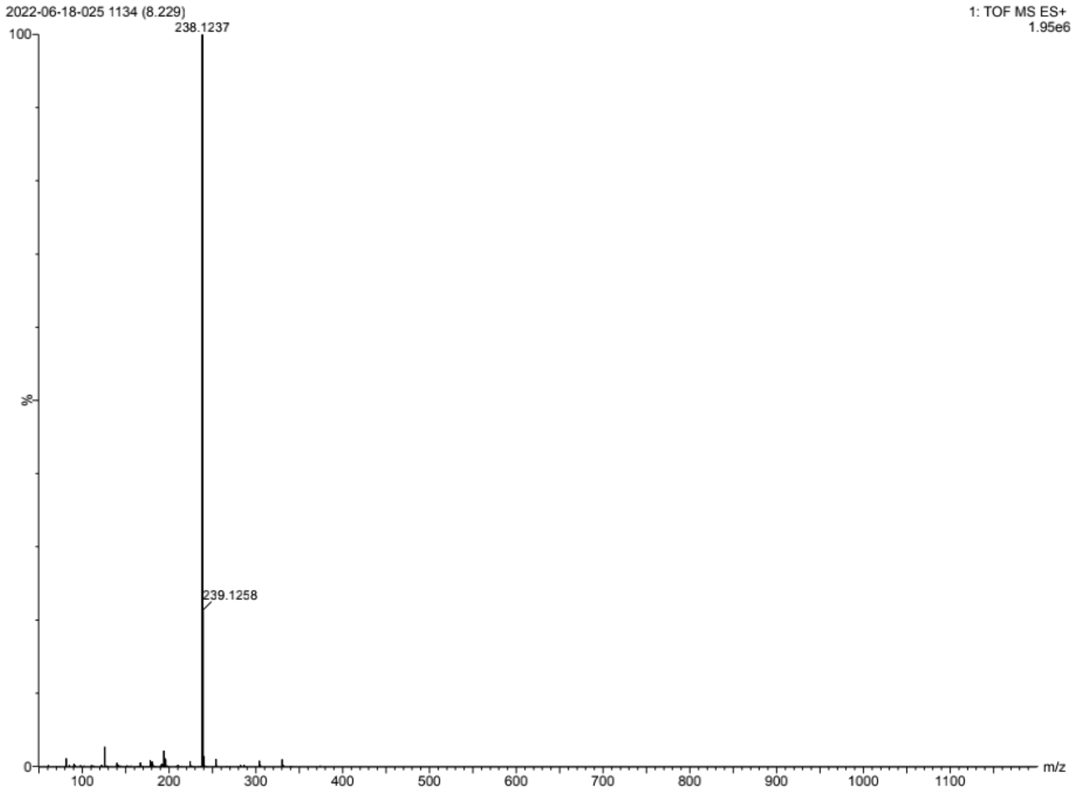

The HRMS of compound 3ck

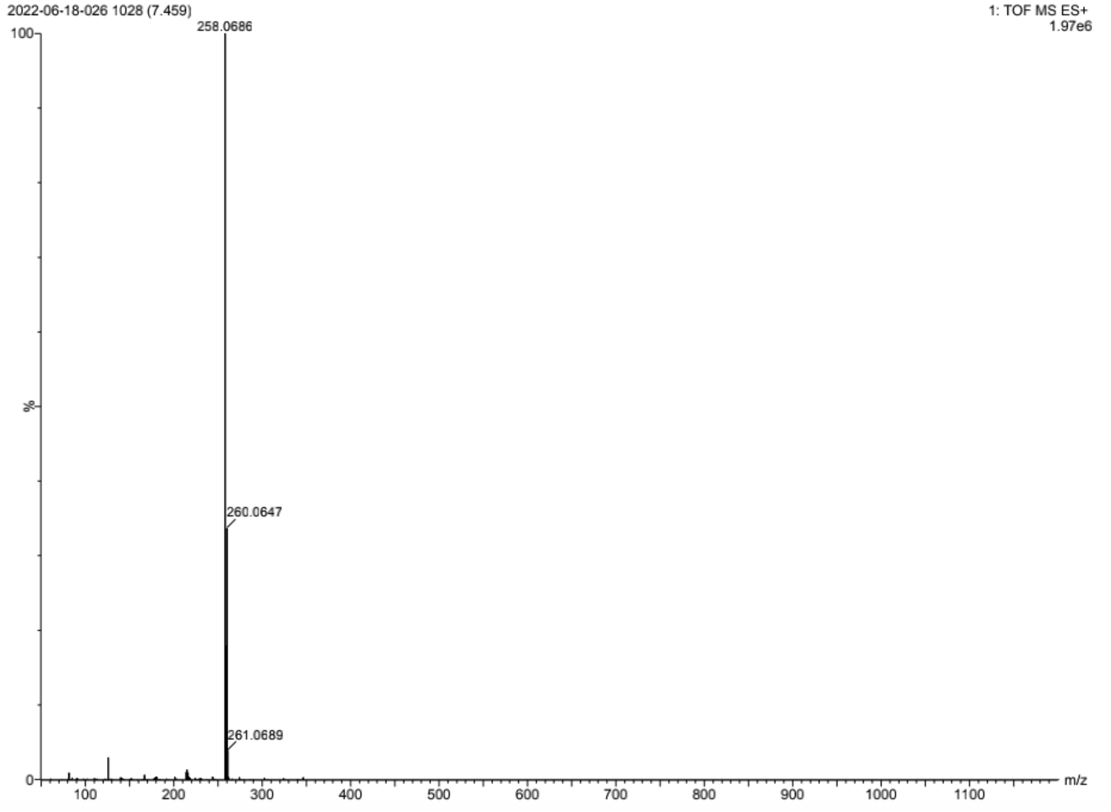

The HRMS of compound 3dk

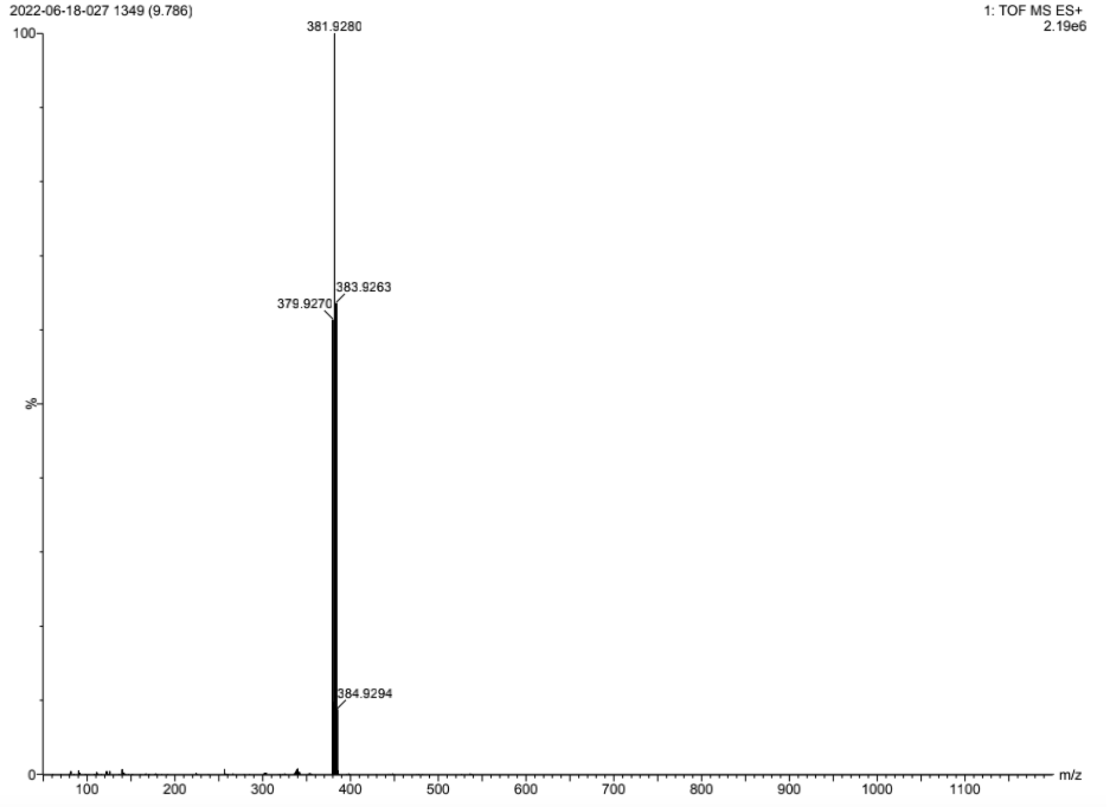

The HRMS of compound 3bl

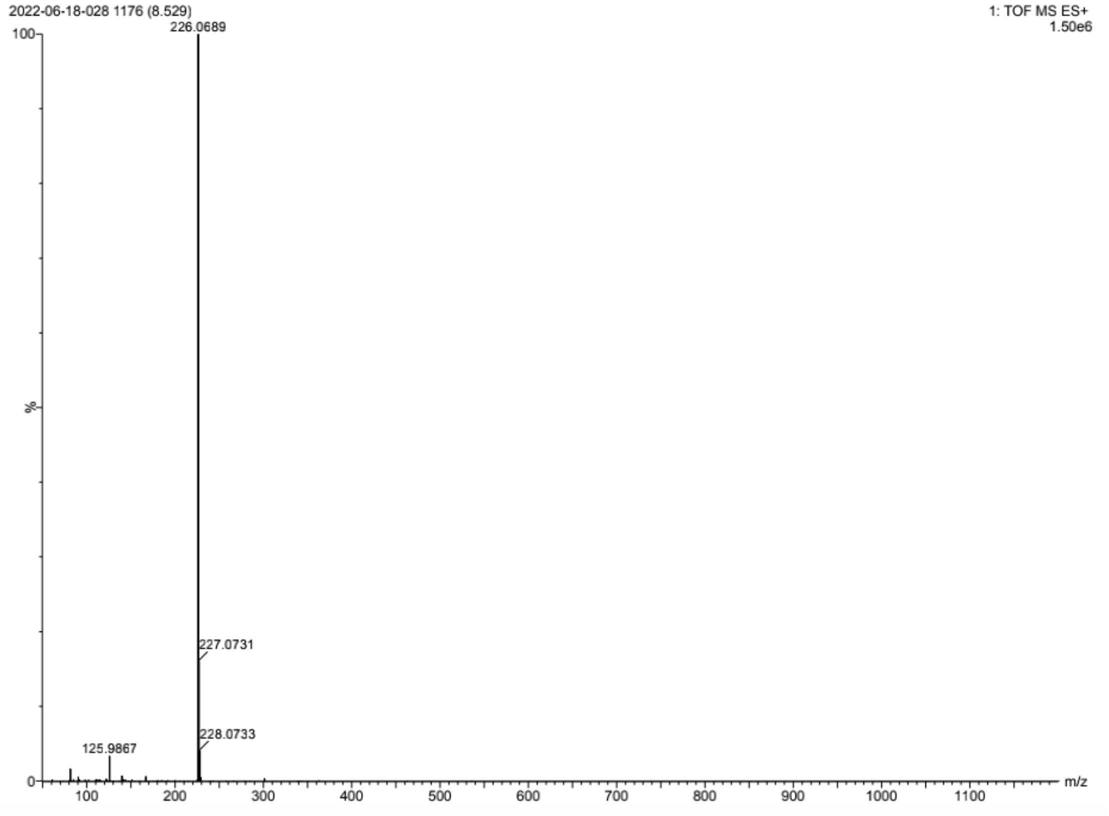

Supplement: File 1 — Experimental procedures, characterization data, copies of 1H and 13C NMR spectra, HRMS of new compounds. [file Beilstein_J_Org_Chem-18-1507-s001.pdf]
